# Solid-Phase Synthesis of Selectively Mono-Fluorobenz(o)ylated Polyamines as a Basis for the Development of ^18^F-Labeled Radiotracers

**DOI:** 10.3390/molecules26227012

**Published:** 2021-11-20

**Authors:** Robert Wodtke, Jens Pietzsch, Reik Löser

**Affiliations:** 1 Institute of Radiopharmaceutical Cancer Research, Helmholtz-Zentrum Dresden-Rossendorf, Bautzner Landstraße 400, 01328 Dresden, Germany; j.pietzsch@hzdr.de; 2 Faculty of Chemistry and Food Chemistry, School of Science, Technische University Dresden, Mommsenstraße 4, 01069 Dresden, Germany

**Keywords:** amide bond reduction, reductive alkylation, ^18^F-labeling, prosthetic groups, site-selective chemical modification, transglutaminases, polyamine transport system, substrate-based probes, PET tracers, tumor targeting

## Abstract

Polyamines are highly attractive vectors for tumor targeting, particularly with regards to the development of radiolabeled probes for imaging by positron emission (PET) and single-photon emission computed tomography (SPECT). However, the synthesis of selectively functionalized derivatives remains challenging due to the presence of multiple amino groups of similar reactivity. In this work, we established a synthetic methodology for the selective mono-fluorobenz(o)ylation of various biogenic diamines and polyamines as lead compounds for the perspective development of substrate-based radiotracers for targeting polyamine-specific membrane transporters and enzymes such as transglutaminases. For this purpose, the polyamine scaffold was constructed by solid-phase synthesis of the corresponding oxopolyamines and subsequent reduction with BH_3_/THF. Primary and secondary amino groups were selectively protected using Dde and Boc as protecting groups, respectively, in orientation to previously reported procedures, which enabled the selective introduction of the reporter groups. For example, *N*^1^-FBz-spermidine, *N*^4^-FBz-spermidine, *N*^8^-FBz-spermidine, and *N*^1^-FBz-spermine and *N*^4^-FBz-spermine (FBz = 4-fluorobenzoyl) were obtained in good yields by this approach. The advantages and disadvantages of this synthetic approach are discussed in detail and its suitability for radiolabeling was demonstrated for the solid-phase synthesis of *N*^1^-[^18^F]FBz-cadaverine.

## 1. Introduction

**Figure 1:**
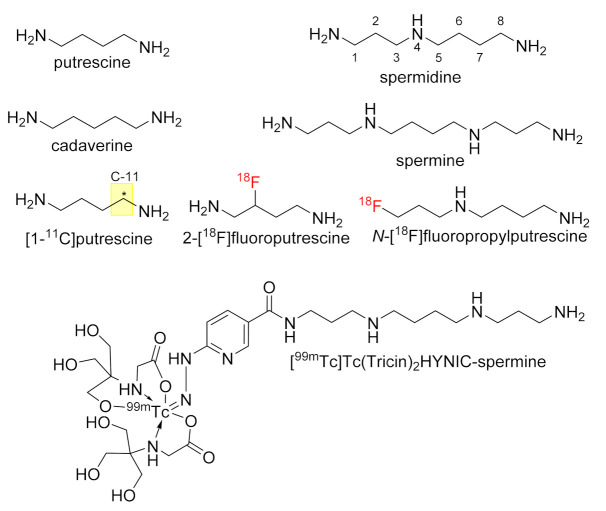
Common naturally occurring polyamines and polyamine-derived PET and SPECT tracers. The asterisk indicates the position of the ^11^C atom.

Polyamines in the more narrow sense represent biogenic amines biosynthetically derived from basic amino acids such as ornithine, lysine and arginine, which often contain additional aminoalkyl groups. In mammals, the most common polyamines are putrescine, spermidine and spermine [1], while plants and bacteria additionally contain the diamine cadaverine [2] (Figure 1). In addition to the simple polyamines, secondary metabolite-type natural products originate mostly from *N*-acylation of simple polyamines and subsequent further structural diversification, which leads to the polyamine alkaloids of plants, bacteria and fungi [3,4], and polyamine toxins, which are present in various species of insects and spiders [5].

Considering their rather simple structure, the physiological functions of polyamines are tremendous. They act as ubiquitous growth regulators in virtually all organisms covering eukaryotes, prokaryotes and archaea [6]. This function is exerted by multiple mechanisms at the transcriptional and translational level of gene expression [7], as DNA and RNA sequences can be stabilized by interaction with the positively charged polyamines [8,9]. Furthermore, spermidine serves as the biosynthetic aminobutyl donor during the posttranslational modification of lysine to hypusine, which uniquely and universally occurs in the eukaryotic initiation factor eIF5A [10,11]. Polyamines can further act as allosteric ligands at the membrane-bound receptors of certain neurotransmitters, as does spermine at ionotropic glutamate receptors of the NMDA subtype [12,13], in addition to at certain ion channels [14]. Further functions of polyamines include the electrostatic stabilization of phospholipidic membranes and their action as antioxidants. The latter function is mainly attributed to their chelating capability towards redox-active metal ions such as iron and copper [15] and their radical-scavenging activity [16]. Polyamines can undergo modifications and degradation by acylation and oxidative deamination, respectively. Acylative modifications occur mainly by terminal acetylation, particularly of spermine and spermidine [17] and, furthermore, by transamidation with protein-bound glutamine residues catalyzed by transglutaminases [18,19,20]. For example, tubulin has been shown to undergo transamidative modification at Gln residues, which was recognized as critical for microtubule function [21]. As implicated by their function as growth regulators, the polyamine content in tumor cells can be up to 4–6 times higher than in normal cells. This is realized both by increased biosynthesis and increased uptake via the polyamine transport system. The increased demand of polyamines can be exploited for both cancer therapy and imaging. For example, depletion of tumor cells from polyamines by inhibiting their synthesis and/or their transport appears a viable cancer therapy [22]. Targeting polyamine transport for tumor imaging by positron emission tomography (PET) and single photon emission computed tomography (SPECT) was pursued with ^11^C-labeled putrescine [23] and ^18^F-labeled analogues [24,25,26], and ^99m^Tc-HYNIC-conjugated spermine [27], respectively (Figure 1). At the cellular level, *N*^4^-fluorophor-conjugated spermidine derivatives were characterized as probes for microscopic imaging of polyamine uptake, which revealed that probe accumulations are significantly increased in MCF-7 breast cancer cells compared to the non-tumorigenic epithelial MCF-10A cell line [28]. Furthermore, due to their DNA-targeting capability, radiolabeled polyamines appear as attractive hydrophilic vectors for endoradionuclide therapy (ENRT), if suitable radionuclides such as Auger electron-emitters in combination with intercalating moieties are employed [29,30]. However, the applicability of polyamines for ENRT-related purposes still needs to be demonstrated.

Although polyamines are established promoters of tumor growth, somewhat contradictorily, these biomolecules, particularly spermidine, seem to act towards cancer chemoprevention [31] and increased polyamine levels in normal tissues have been correlated with longevity [32].

The overwhelming biological significance and potential biomedical applications of polyamines stimulated the development of methods for their chemical synthesis [33]. Regarding the synthesis of conjugates with reporter groups carrying fluorophores or radionuclides the regioselective *N*-functionalization of polyamines often requires a complex combination of protection groups [34]. In this respect, elegant polyamine syntheses in solution were developed [5], which involved the use of *N*^4^,*N*^8^-formaldehyde-protected spermidine [35]. A further approach used *N*^1^-Boc, *N*^4^-tosyl-protected putrescine as a building block, in which the tosyl group acts both as protecting and activating group towards *N*-alkylation with 3-aminoalkyl synthons [34]. A similar approach involving *N*-nosyl-protected diaminoalkane precursors was pursued in the synthesis of spermine, thermospermine and norspermine [36]. The drawback of handling the polar amino compounds in solution can be overcome by solid-phase synthesis, as it enables the separation of excessive educts by simple and fast filtration and washing, which has revealed to be a major breakthrough in the synthetic access to the polyamine skeletons [37]. Furthermore, combinatorial polyamine collections in the product scale <100 mg can be accessed in that manner [37,38,39,40]. Reactions required for the construction of the polyamine backbone, such as S_N_2 alkylations [41], Fukuyama alkylations (particularly under Mitsunobu conditions) [42] and reductions of amides with borane [43], in addition to reductive aminations [44], were demonstrated to be compatible with solid-phase synthesis. Hall et al. described an interesting route for the synthesis of selectively functionalized polyamines on solid phase [45]. In this approach, the oxopolyamine chain assembled stepwise by amide bond coupling is reduced with borane and the distinction between primary and secondary amino groups is achieved by subsequent introduction of the Dde protecting group, which allows selective protection of primary amino groups. Subsequent Boc protection of secondary amino groups, followed by cleavage of the Dde protecting group, derivatization of the released primary amino group and cleavage from the resin yields the corresponding terminally functionalized polyamines. Trityl chloride resin served as the polymeric support and the borane-amine adducts resulting from reduction need to be cleaved under mildly oxidative conditions, for which Hall’s group developed treatment with iodine in an HOAc/DIPEA buffer [46,47]. Using this route, Hall et al. succeeded in synthesizing the spider toxin *δ*-philanthotoxin (PhTX-4.3.3) of the solitary wasp *Philanthus triangulum* in 55%, among other synthetic targets [45]. In contrast to asymmetric polyamine backbones, as in the case of PhTX-4.3.3, symmetric polyamine backbones can be directly attached to the polymeric support and the amino groups subsequently differentially protected, as mentioned previously. This was demonstrated by Bycroft et al. for the direct attachment of spermine to the 2-ClTrtCl resin and subsequent protection of the primary and secondary amino groups with Dde and Boc, respectively, in the context of the PhTX-4.3.3 synthesis, even before Hall’s approach was published [48].

Applying the synthetic concepts developed by Hall et al. and Bycroft et al. for the synthesis of polyamine toxins to access fluorobenzoylated polyamines and oxopolyamines as potential PET tracers for imaging polyamine transport and transglutaminase activity was the aim of this study. The synthesis of various derivatives is described and discussed herein. The 4-fluorobenzoyl group was chosen as prosthetic group for potential labeling with fluorine-18, as the radiosynthesis of the corresponding reagent *N*-succinimidyl 4-[^18^F]fluorobenzoate ([^18^F]SFB) can be reliably achieved in an automated manner [49,50,51] and its use for ^18^F-fluorobenzoylation on solid phase has been demonstrated by us [52,53].

In support of the pharmacological suitability of the 4-fluorobenzoyl group, it should be emphasized that hydroxybenzoylated polyamines were identified as natural products, such as the fungal spermidine derivative pistillarine (*N*^1^,*N*^8^-bis-(3,4-dihydroxybenzoyl)spermidine) [54] and the bacterial siderophore *N*^1^,*N*^8^-bis-(2,3-dihydroxybenzoyl)spermidine [55], in addition to spermidine derivatives containing unsubstituted benzoyl groups that were occasionally isolated from plant sources [56,57]. *N*^1^,*N*^8^-bis-(2,3-dihydroxybenzoyl)spermidine was considered for iron chelation therapy and was demonstrated to undergo fast intestinal absorption in this context [58]. Worth of note, pistillarine shows antimalarial activity in *Plasmodium falciparum*-infected erythrocytes [59]. This suggests that substituted benzoyl groups are tolerated by the polyamine-transport system. Regarding the situation of its reduced analogue, the 4-fluorobenzyl group, 4-fluorobenzylated polyamines were shown to exhibit suitable transport characteristics in tumor cells [60]. Therefore, it can be concluded that both 4-fluorobenzoyl and 4-fluorobenzyl groups are appropriate reporter groups for the development of polyamine-derived imaging probes based on labeling with fluorine-18.

## 2. Results and Discussion

### 2.1. Solid-Phase Synthesis of Fluorobenzoylated Polyamines

**Scheme 1:**
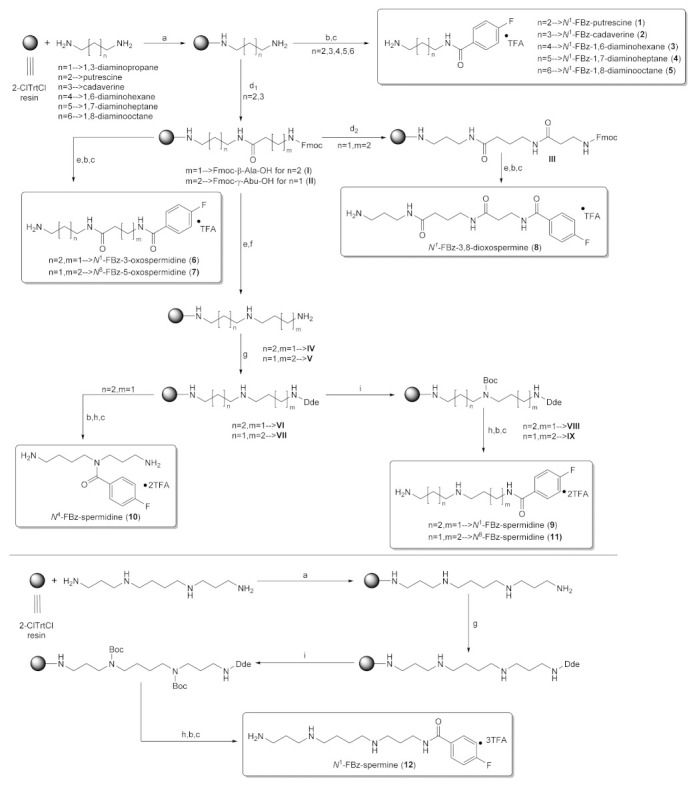
Routes to compounds **1**–**12**. Reagents and conditions: (a) 1. Diamine/polyamine, CH_2_Cl_2_, o/n, 2. CH_2_Cl_2_/CH_3_OH/DIPEA (17/1/2); (b) 4-FBz-Cl, TEA, DMF, 2 h; (c) TFA/TES/H_2_O (95/2.5/2.5), 2 h; (d_1_) Fmoc-amino acid, HBTU, DIPEA, DMF; (d_2_) 1. 20% piperidine/DMF, 2. Fmoc-*β*-Ala-OH, HBTU, DIPEA, DMF (e) 20% piperidine/DMF, 3 × 20 min, 1 × 45 min; (f) 1. 1 M BH_3_/THF, 65 °C, 40 h, Argon, 2. I_2_, THF/DIPEA/HOAc (7/1/2), 4 h; (g) Dde-OH, o/n; (h) 2% N_2_H_4_/DMF, 4–10 × 15 min; (i) Boc_2_O, DIPEA, CH_2_Cl_2_, o/n.

Using the solid-phase approaches outlined above, *N*^1^-FBz-spermidine (**9**), *N*^8^-FBz-spermidine (**11**) and *N*^1^-FBz-spermine (**12**) were envisaged to be synthesized. Furthermore, *N*^4^-FBz-spermidine (**10**) should also be accessible in this way, as spermidine contains only one secondary amino group, which can be fluorobenzoylated after protection with the Dde group. *N*-FBz-putrescine (**1**), *N*^1^-FBz-3-oxospermidine (**6**), *N*^8^-FBz-5-oxospermidine (**7**) and *N*^1^-FBz-3,8-dioxospermine (**8**), as further potential TGase 2 substrates, are directly accessible from the synthetic routes to **9**, **10** and **11**. The further fluorobenzoylated diamines (**2**–**5**) can be obtained analogously to **1** after attachment of the respective diamines to the resin and subsequent fluorobenzoylation. An overview of the synthetic routes to compounds **1**–**12** is given in Scheme 1.

The route reported by Hall et al. [45] was initially tested and optimized for the synthesis of *N*^1^-FBz-spermidine (**9**). In this context, the particular steps at solid-phase were monitored by mini-cleavage using 1–3 mg of resin. In the following paragraphs, the individual synthesis steps shown in Scheme 1 is discussed in detail. Subsequently, the results for compounds **1**–**12** are compared.

In line with Bycroft et al., the polystyrene-based 2-ClTrtCl resin was chosen as polymeric support for attachment of the symmetric polyamines. The functionalization degree with reactive chlorotrityl chloride linker was 1.55 mmol/g. For loading of the related trityl chloride resin (0.8 mmol/g), Hall et al. employed 50 equivalents of polyamine, which were added in four portions as solution in CH_2_Cl_2_ resulting in 0.73 mmol/g of resin-bound 1,3-diaminopropane. Considering the almost double initial loading of the employed 2-ClTrtCl resin, complete substitution with amine did not seem to be necessary. Therefore, four equivalents of putrescine were reacted with the 2-ClTrtCl resin and the reaction time extended to 12–14 h, according to the procedure of Egner et al. [61] During the loading reaction, the formation of a white precipitate was observed immediately after the addition of the symmetric polyamines, which is most likely due to the formation of the corresponding polyamine hydrochlorides resulting from released HCl. Even though this reduces the availability of primary amino groups as reactions partners, partial alkylammonium chloride formation should not significantly compromise the loading yield, because the polyamines are present in excess. Treatment with a solution of CH_2_Cl_2_/CH_3_OH/DIPEA (17/1/2), for quenching of unreacted chlorotrityl chloride groups concomitantly dissolved the precipitate. For loading of putrescine, cadaverine and spermine, the loading levels were determined gravimetrically using the equation in Section 3.2.1. Loading levels of 0.90, 1.22 and 1.05 mmol/g were achieved for putrescine, cadaverine and spermine, respectively, which are sufficiently high for the further reactions on the resin. No tendency towards better binding of certain polyamines was discernable, because the loading degrees vary to only small extent.

**Figure 2:**
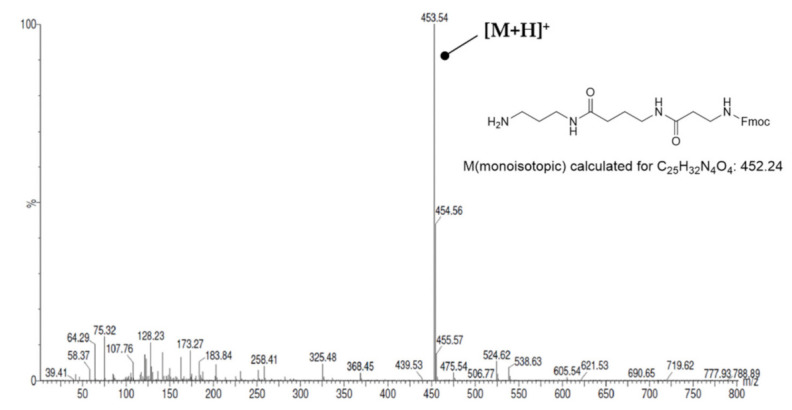
ESI(+) mass spectrum of *N*^1^-Fmoc-3,8-dioxospermine (**III**).

Starting from 1,3-diaminopropane and putrescine, three different resin-bound Fmoc-protected oxopolyamines (**I**–**III**, Scheme 1) were assembled. Using HBTU-mediated and MW-assisted amide bond coupling, Fmoc-*γ*-Abu-OH and Fmoc-*β*-Ala-OH were attached to resin-bound 1,3-diaminopropane and putrescine, respectively, to obtain the corresponding 5/3-oxospermidine (**II**/**I**) scaffold. For assembling *N*^1^-Fmoc-3,8-dioxospermine (**III**), Fmoc-*γ*-Abu-OH was coupled to 1,3-diaminopropane, followed by coupling of Fmoc-*β*-Ala-OH after removal of the Fmoc group with 20% piperidine/DMF under MW irradiation. The construction of the three different Fmoc-protected oxopolyamines succeeded without problems in numerous experiments, which was confirmed, first, by the weight increase in the resin and, second, by the ESI mass spectra of the mini-cleavages. For illustration, the ESI(+) mass spectrum after the assembly of *N*^1^-Fmoc-3,8-dioxospermine (**III**) is shown in Figure 2, which is characterized by the almost exclusive presence of the compound’s [M + H]^+^ signal at *m*/*z* = 453.5. The loading degree after assembling the oxopolyamines was determined using the method reported by Gude et al. [62], which allows for quantification of the Fmoc-protecting groups present on the resin by DBU-mediated cleavage and subsequent photometric detection of the released dibenzofulvene by extinction measurement at 294 nm. Loading levels in the range of 0.5–0.7 mmol/g were achieved for *N*^1^-Fmoc-3-oxospermidine (**I**), *N*^8^-Fmoc-5-oxospermidine (**II**) and *N*^1^-Fmoc-3,8-dioxosperimine (**III**) within numerous syntheses. Fmoc removal without MW irradiation was performed by repeated treatment with 20% piperidine/DMF (3 × 20 min and 1 × 45 min), after which no signals for the Fmoc-protected oxopolyamines were detectable in the mass spectra.

**Figure 3:**
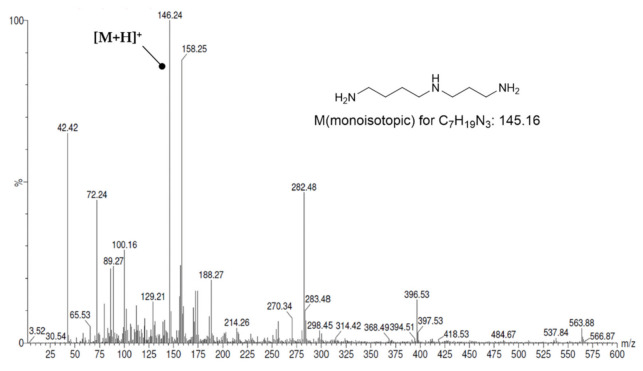
ESI(+) mass spectrum of spermidine obtained after reduction of resin-bound 3-oxospermidine (**IV**).

The reduction of primary, secondary and tertiary amides to the corresponding amines can be achieved using BH_3_ in complex with Lewis bases as electron-deficient hydride donor, which usually leads to good results even in the presence of other electrophilic functionalities such as nitro, halo, ester, sulfone and carbamate groups [63,64]. In the case of secondary amides, borane complexes of borylated amines are obtained [65]. Both N-B bonds need to be cleaved to deliver the desired secondary amine, while the borane-amine adduct (N-BH_3_) is much more stable than the aminoborane (N-BH_2_) moiety [66]. This can be achieved under strongly basic [67] or acidic conditions [68], which is not compatible with various acid- and base-labile protecting groups or linkers. Hall’s group tackled this challenge by developing a method for N-B bond cleavage under mildly oxidative conditions. Subjecting the resin-bound borane adducts to HOAc/DIPEA buffer (THF/DIPEA/HOAc 17/1/2) leads to release of the amine without compromising the integrity of the trityl amine linkage [46,47]. According to the investigations of Manku et al., amine release by this method involves the stepwise oxidative substitution of boron-bound hydrogen by iodine, each followed by nucleophilic attack of boron by acetate, which finally leads to the formation of triacetyl borate [46]. For the reduction of the two oxospermidine derivatives **I** and **II** to **IV** and **V** (Scheme 1), the conditions for treatment with BH_3_/THF as stated by Hall et al. were slightly modified. Thus, 2 × 20 equivalents of BH_3_/THF were applied per amide bond instead of 1 × 12 and the reaction time was reduced from 48 to 40 h. The workup procedure was adopted using twelve equivalents of iodine per borane-amine adduct in THF/DIPEA/HOAc (17/1/2; 10 mL/g resin) and a reaction time of 4 h. Confirmation of the successful reduction of the two oxospermidine derivatives was obtained from the ESI(+) mass spectra of the mini-cleavages, in which no signals for oxospermidine (monoisotopic mass for C_7_H_17_N_3_O: 159.23) were evident, as is obvious from Figure 3.

As mentioned previously, the reduction of amides can occur selectively in the presence of other reducible functional groups, e.g., carbamate groups [69]. Therefore, *N*^1^-Fmoc-3-oxospermidine (**I**) was subjected to reduction with borane and subsequent oxidative workup. A successful selective amide reduction would have had the consequence that a distinction between the primary and the secondary amino group after reduction would already have been given by the Fmoc-protecting group and the “detour” via the Dde protecting group (as described in the following paragraph) could have been avoided. However, the Fmoc-protecting group was found to be partially cleaved under conditions of the reduction. Dde protection of the putative *N*^1^-Fmoc-spermidine on the resin gave clear signals for Dde-protected spermidine in the ESI mass spectra of the sample cleavage (data not shown). Because the Dde protecting group reacts selectively with primary amino groups, cleavage of the Fmoc-protecting group must have occurred during reduction. The reason for this finding might be associated with the acidic 9*H* proton of the fluorenyl residue, which may react with BH_3_ analogously to the amide protons, which consequently causes the cleavage of the protecting group. In the literature, Fmoc cleavage by BH_3_/THF is rarely explicitly mentioned. However, Wen and Crews were able to observe formation of dibenzofulvene even at −78 °C in their experiments on the reduction of Fmoc amino acids into the corresponding Fmoc amino alcohols [70]. Furthermore, shortening of the synthetic pathway to *N*^4^-FBz-spermidine (**10**) was attempted. For this purpose, Boc-*β*-Ala-OH was coupled to the putrescine-loaded 2-ClTrtCl resin instead of Fmoc-*β*-Ala-OH. The subsequent successful selective reduction of the amide in the presence of the Boc-protecting group would leave only the secondary amine, thus allowing direct fluorobenzoylation. To characterize whether the Boc-protecting group remained stable during the reduction, mini-cleavage from the resin was performed with CH_2_Cl_2_/HFIP (4/1), which cleaves the amine from the resin but not the Boc-protecting group [71]. However, the corresponding ESI(+) mass spectrum (see Figure S1 in Supplementary Materials) did not exhibit signals that could be assigned to *N*^1^-Boc-spermidine (the [M + H]^+^ peak would appear at *m*/*z* = 246). Instead, a signal at *m*/*z* = 160.3 was observed, which was assigned to *N*^1^-Methyl-spermidine (monoisotopic mass for C_8_H_21_N_3_: 159.17). The ^1^H NMR spectrum of the sample obtained by mini-cleavage confirmed the presence of the methyl group (triplet at *δ* = 2.57 ppm), which arises from the reduction of the Boc carbonyl group. Reduction of *N*-Boc groups to *N*-Methyl by borane treatment is documented [72,73]. According to Amedio et al., Boc reduction can be attenuated to an acceptable level by careful optimization of reaction parameters, which, however, was not considered in this study.

**Figure 4:**
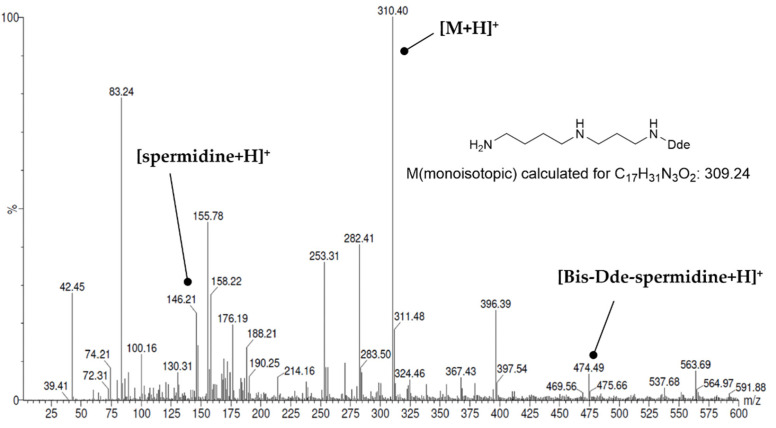
ESI(+) mass spectrum after protection of resin-bound spermidine as Dde-spermidine (**VI**).

Originally, Dde (*N*-(1(4,4-dimethyl-2,6-dioxohexylidene)ethyl)) was developed as a protecting group for peptide synthesis, which is completely orthogonal to the deprotection conditions for Boc and Fmoc protecting groups [74,75]. Later, it was recognized that it can be used for selective protection of primary amines in the presence of secondary amines [48]. This selectivity is probably due to the stabilization of the resulting secondary enamine by an intramolecular hydrogen bond, which cannot be formed in the case of secondary amino groups. Hall et al. used two equivalents of Dde-OH at a reaction time of 2 h for protection of the primary amino groups. The same stoichiometry was applied for Dde protection of the various polyamines reported herein, but the reaction time had to be extended to 12–14 h. This probably reflects the shorter length of the polyamine chains assembled herein compared to Hall’s study, which in turn result in greater steric hindrance by the polymeric support and thus slower kinetics for the reaction with Dde-OH. The ESI(+) mass spectrum recorded after Dde protection shows a main peak at *m*/*z* = 310.4, which corresponds to the [M + H]^+^ signal of *N*^1^-Dde-spermidine (**VI**) and thus indicates the success of the reaction (see Figure 4). In addition, however, signals for spermidine (monoisotopic mass for C_7_H_19_N_3_: 145.16) and for doubly Dde-protected spermidine (monoisotopic mass for C_27_H_43_N_3_O_4_: 473.33) can also be detected (see Figure 4). This shows that the protection of primary amino groups is not complete despite the long reaction time (mass signals arising from fragmentation can be excluded). In addition, however, there is no 100% selectivity for primary amino groups. This result is slightly contradictory to the observations reported by Hall et al., which suggest a quantitative Dde protection on the basis of a negative ninhydrin assay. However, taking into account the low abundance of bis-Dde-spermidine, the selected reaction conditions can be considered satisfactory and were therefore also applied for the Dde protections of the other polyamines. Nevertheless, side-products were detectable due to the incomplete and partially non-selective reaction with Dde-OH. In the case of the synthesis of *N*^4^-FBz-spermidine (**10**), reaction of Dde-protected, resin bound spermidine with 4-fluorobenzoyl chloride led to the formation of *N*^1^,*N*^4^-bis-FBz-spermidine, in addition to *N*^1^-Dde, *N*^4^-FBz-spermidine, as detected by mass spectrometry (see Figure S2 in Supplementary Materials). The formation of fluorobenzoylated side-products was less problematic for the synthesis of the terminally modified polyamine derivatives **9**, **11** and **12**, because free amino groups are instantaneously capped by treatment with Boc_2_O, which was primarily employed for protection of the secondary amino groups. Both kinds of side-products, bis-FBz-spermidine for the synthesis of **10**, and unmodified spermidine and spermine for the syntheses of **9**, **11**, and **12**, do not pose any problem in the purification of the crude products, because they are much more lipophilic and hydrophilic than the products, respectively, and thus can be well separated by RP-HPLC.

A disadvantage of the Dde protecting group is the transfer to other primary amino groups in the same molecule [38]. Therefore, mono-protection of molecules with multiple primary amino groups is not possible. However, the susceptibility of Dde to primary amino groups is exploited for its cleavage, for which treatment with 2% hydrazine in DMF has been established as a suitable method. The driving force in the cleavage represents the irreversible formation of 3,6,6-trimethyl-4-oxo-4,5,6,7-tetrahydro-1*H*-indazole, the formation of which can be followed at 300 nm (or also 270 or 290 nm [74,76]). For the cleavage of the Dde protecting group from the polyamine derivatives, the resin was repeatedly treated with 2% hydrazine in DMF for 15 min each, and the extinction at 300 nm was determined for all filtrates. Compared to spermidine, the Dde cleavage of spermine required fewer hydrazine treatments. This can be likely attributed to the longer chain length of spermine, which reduces the steric hindrance imparted by the polymeric support and thus makes the nucleophilic amino groups more accessible. In all cases, the Dde protecting group could be quantitatively removed.

As mentioned previously, Boc protection of the secondary amino groups after introduction of the Dde group (resin-bound compounds **VIII** and **IX**, Scheme 1) was carried out with di-*tert*-butyl carbonate (Boc_2_O). In contrast to the procedure of Hall et al. twelve equivalents of this reagent and six equivalents of Hünig’s base were employed per amino group, in order to ensure capping of the remaining unreacted primary amino groups. The success of Boc protection was confirmed by mini-cleavage from the resin with CH_2_Cl_2_/HFIP (4/1) followed by ESI-MS analysis.

The final attachment of the 4-fluorobenzoyl group was achieved by using 4-fluorobenzoyl chloride in the presence of stoichiometric amounts of TEA. Fluorobenzoylation always proceeded quantitatively for both the primary amino groups and the secondary amino group using 2.5 equivalents of 4-FBz-Cl at a reaction time of 2 h. The subsequent final cleavage from the resin, in addition to the intermediate mini-cleavages, were essentially as described by Hall et al., with the exception of using triethylsilane instead of triisopropylsilane as a nucleophilic scavenger for the resulting 2-chlorotritylium ions in addition to water. Release from resin under these conditions is accompanied by removal of the Boc group, resulting in the formation of the mono-fluorobenzoylated polyamines **1**–**12** (Scheme 1), which were purified by RP-HPLC to obtain the corresponding mono-, di- or tri- trifluoroacetate salts as oils or waxy solids. The absolute amounts and corresponding yields for the syntheses of compounds **1**–**12** are summarized in Table 1.

The high yields afforded for compounds **6**–**8** are in agreement with expectation because the synthesis steps carried out following the loading determination for the Fmoc-protected oxopolyamines (Fmoc cleavage, fluorobenzoylation and cleavage from the resin) were expected to proceed quantitatively. In this context, the yields for compounds **1**–**5** are unexpectedly low, as here only fluorobenzoylation and cleavage from the resin were performed after loading determination. The reason for this is most likely due to the less accurate gravimetric method for loading determination applied for the resin-bound diamines compared to quantification of the Fmoc protecting groups present on the resin. The loading levels appear to be slightly too high, which subsequently explains the somewhat low yields. The yields for spermidine derivatives **9**–**11** are significantly lower than those for the first five polyamine derivatives. This is probably due to the Dde protection step, which was—as mentioned previously—incomplete with respect to the primary amino groups, and also resulted in bis-Dde-protected polyamines. Both insufficiencies reduce the yield of the respective target polyamine. A further reason for the reduced yields may be the thermal instability of the amine-chlorotrityl linkage at the elevated temperature and the presence of borane as the Lewis base during amide bond reduction, because the chlorotrityl group can be potentially released by heterolytic thermal cleavage [77]. Nevertheless, the yields obtained after a total of six (in the case of **10**) or seven (in the case of **9** and **11**) synthesis steps (starting from Fmoc-protected oxopolyamines) can be regarded as highly satisfactory. For comparison, Hall et al. obtained the polyamine-based toxins PhTX-433 and HO-416b after analogous synthesis steps, in yields of 54 and 37%, respectively. Thus, the yields for the spermidine derivatives are close to these values. Moreover, the small range of variation proves the good reproducibility of the synthetic route adapted from the procedure published by Hall et al. [45].

The rather good yields for the monofluorobenzoylated spermidines **9**–**11** in respect to their complex synthesis encourages labeling with fluorine-18. Considering the physiological function of their naturally occurring acetyl-based analogues, *N*^1^- and *N*^8^-acetylspermidine, radiolabeled probes based on compounds **9** and **11** appear as highly interesting. *N*^1^-Acetylspermidine is formed in the cytosol and can undergo carrier-mediated transport across the plasma membrane [78]. In contrast, *N*^8^-acetylspermidine is formed in the nucleus and has been identified as the physiological substrate of HDAC10 [79,80,81], which has far-reaching oncopharmacological implications [82]. Hence, [^18^F]**11** represents a potential substrate-based radiotracer for imaging of HDAC10, as lysine-derived 4-fluorobenzamides were identified as suitable substrates for other HDAC isoenzymes [83]. In addition, **9**, **10** and **11** represent potential polyamine transport substrates [28,84]. In contrast, the monobasic oxopolyamines **6**, **7** and **8** should not be recognized by the polyamine transport system but act as substrates of transglutaminases, which should thus allow for the dissection of polyamine transport and transglutaminase-catalyzed conversion. The terminally fluorobenzoylated spermidines **9** and **11** should be capable of dual targeting as they represent both potential transport and transglutaminase substrates [85,86].

Even though amide-bond reduction on solid phase could be well established for the access to the various fluorobenzoyl-spermidines **9**, **10** and **11**, the symmetric structure and the moderate costs of spermine suggest loading to the 2-ClTrtCl resin and subsequent Dde/Boc protection as a more straight-forward approach for the synthesis of terminally functionalized derivatives such as *N*^1^-(4-fluorobenzoyl)-spermine (**12**). However, the yield for spermine derivative **12**, which is only 10%, strongly deviates from the average yield achieved for the spermidine derivatives. Similar to the simple diamines putrescine and cadaverine, spermine was bound directly to the resin and the loading level determined gravimetrically, which is associated with the inaccuracy discussed above. The ^18^F-labeled analogue of **12** represents a potential PET tracer for imaging of polyamine transport [28,84].

**Figure 5:**
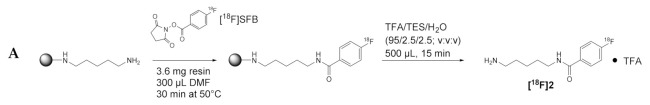

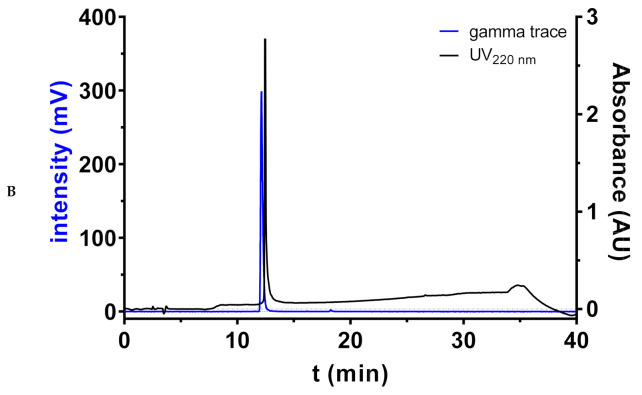
(**A**) Radiosynthetic route to compound [^18^F]**2**. (**B**) Radio-HPLC chromatogram of crude *N*^1^-[^18^F]FBz-cadaverine ([^18^F]**2**) as obtained after cleavage from solid support. A quantity of 3.6 mg (3.6 µmol) of resin-bound cadaverine (pre-swollen in DMF) was reacted with 776 MBq of [^18^F]SFB in DMF (300 µL) at 50 °C for 30 min. Subsequently, the resin was washed with DMF and 2 × CH_2_Cl_2_ (each 2 mL) and remaining radioactive resin (550 MBq) was treated with TFA/Et_3_SiH/H_2_O 95:2.5:2.5 (*v*/*v*/*v*; 500 µL) at 50 °C for 15 min. After this, the suspension was filtered and the resin washed with CH_2_Cl_2_ (500 µL). The combined filtrate was evaporated in an N_2_ stream at elevated temperature, upon which 344 MBq of crude [^18^F]**2** were obtained after a synthesis time of 75 min, which was subjected to radio-HPLC analysis. For comparison, the HPLC chromatogram of non-radioactive **2** from a separate run is shown. The following chromatographic conditions were applied: stationary phase: Phenomenex Luna C18 5 µm column (250 × 4.6 mm); mobile phase 0–3 min isocratic 5% CH_3_CN/H_2_O, 3–25 min gradient to 95% CH_3_CN/H_2_O, 25–30 min isocratic 95% CH_3_CN/H_2_O, 30–35 min gradient back to 5% CH_3_CN/H_2_O, 35–40 min isocratic 5% CH_3_CN/H_2_O, eluent contained 0.1% TFA, flow rate 1 mL/min.

Regarding the radiosynthetic access to ^18^F-labeled diamines and polyamines, we performed pilot experiment towards *N*^1^-[^18^F]FBz-cadaverine ([^18^F]**2**) by reacting cadaverine-loaded resin with *N*-succinimidyl 4-[^18^F]fluorobenzoate ([^18^F]SFB [49,50,51], Figure 5A). This was done in orientation to the procedures for site-selective labeling of peptides on resin with [^18^F]SFB, which were established by us in the past and proved to be efficient for various peptides [52,53]. After cleavage from the resin, HPLC analysis revealed the formation of the desired radiolabeled product [^18^F]**2** in high radiochemical purity of 99%, as indicated by the radio-chromatogram shown in Figure 5B. A total of 344 MBq of chemically crude [^18^F]**2** was obtained starting from 776 MBq of [^18^F]SFB within 75 min of synthesis time, which corresponds to a radiochemical yield (decay-corrected) of 71%. Therefore, this preliminary result also demonstrates the potential of this procedure for application towards the ^18^F-labeling of spermidine and spermine derivatives. However, the procedure still needs to be optimized regarding reaction conditions and purification of the radiolabeled amines. Although ^18^F-acylation of unprotected symmetric polyamines such as spermine is also possible in solution due to sub-stoichiometric amounts of [^18^F]SFB [87], the solid-phase approach appears much more favorable for the radiosynthesis of the spermidine derivatives [^18^F]**9**–**11**.

### 2.2. Solid-Phase Synthesis of Fluorobenzylated Polyamines (Diamines)

**Scheme 2:**
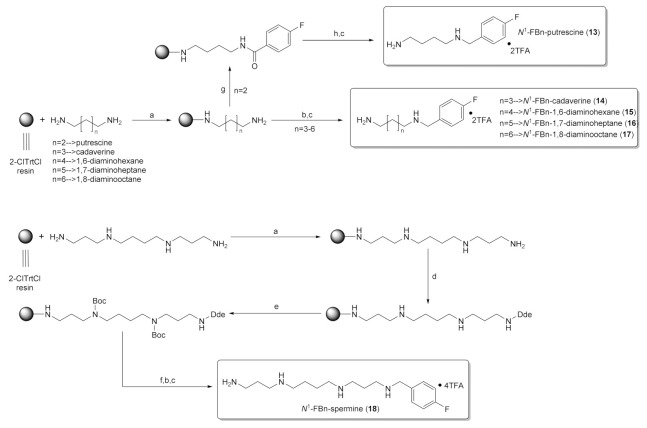
Routes to compounds **13**–**18**. Reagents and conditions: (a) 1. Diamine/polyamine, CH_2_Cl_2_, o/n, 2. CH_2_Cl_2_/CH_3_OH/DIPEA (17/1/2); (b) 1. 4-fluorobenzaldehyde, THF, 3.5 h, 2. Sodium triacetoxyborohydride, THF, 3 h; (c) TFA/TES/H_2_O (95/2.5/2.5), 2 h; (d) Dde-OH, o/n; (e) Boc_2_O, DIPEA, CH_2_Cl_2_, o/n; (f) 2% N_2_H_4_/DMF, 4–10 × 15 min; (g) 4-FBz-Cl, TEA, DMF, 2 h; (h) 1. 1 M BH_3_/THF, 65 °C, 40 h, Argon, 2. I_2_, THF/DIPEA/HOAc (7/1/2).

For the synthesis of fluorobenzylated polyamines, a similar synthetic strategy was followed as for the amide analogs, which differed in the last step by employing on-resin reductive alkylation with 4-fluorobenzaldehyde using sodium triacetoxyborohydride (NaBH(OAc)_3_) as reducing agent (Scheme 2) instead of 4-fluorobenzoylation. Initially, 1.5 equivalents of 4-fluorobenzaldehyde were employed in the presence of 2 equivalents of NaBH(OAc)_3_, which led to the formation of the desired product accompanied by the formation of dialkylated side-product (see Figure S3 in Supplementary Material). Thereupon, the reaction conditions were adjusted and the most favorable conditions were identified as follows: imine formation with 1 equivalent of 4-fluorobenzaldehyde followed by a washing step to remove unreacted aldehyde and then, imine reduction by the addition of NaBH(OAc)_3_ (1.5 equivalents). Notably, the dialkylated side-product was still observed, however, to a much lesser extent compared to the one-step procedure. The fluorobenzylated diamines **14**–**17** and *N*^1^-FBn-spermine (**18**) were obtained in moderate yields (14–36%) by this approach (Table 1).

For the synthetic access to *N*^1^-FBn-putrescine, the amide bond in resin-bound FBz-putrescin was reduced with BH_3_/THF according to the synthesis of the spermidine scaffold described in Section 2.1. This procedure was also successful and the yield was slightly higher compared to the reductive alkylation (40%, Table 1) at the cost of a more complex reaction handling (60 °C, argon).

For all compounds in this study, the respective purity, structure and identity was unambiguously confirmed by ESI mass spectrometry and NMR spectroscopy (^1^H, ^13^C, ^19^F; see Figures S4–S39 for ^1^H and ^13^C NMR spectra).

Although the radiosynthesis of ^18^F-fluorobenzylated polyamines was not tested in the present study, a reliable access to such derivatives, similar to the synthesis of compounds **14**–**18**, might be possible by reductive alkylation of the respective resin-bound polyamines with the well-known prosthetic labelling agent 4-[^18^F]fluorobenzaldehyde and NaBH(OAc)_3_ or NaBH_3_CN [88,89,90]. Furthermore, ^18^F-fluorobenzamides can be transformed into the corresponding ^18^F-fluorobenzylamines by treatment with BH_3_, as demonstrated by Kügler et al. [91]. Therefore, on-resin amide bond reduction of the respective ^18^F-fluorobenzoylated polyamines with BH_3_/THF should be possible, which, moreover, may represent a strategy for the sequential access to ^18^F-fluorobenz(o)ylated polyamine pairs by using [^18^F]SFB as prosthetic agent.

## 3. Materials and Methods

### 3.1. General

Educts, reagents and solvents were purchased from Acros Organics (Geel, Belgium), Alfa Aesar (Kandel, Germany), Bachem (Bubendorf, Switzerland), Fluka (Buchs, Switzerland), Fischer Scientific (Schwerte, Germany), Merck (Darmstadt, Germany), Multisyntech (Witten, Germany), Roth (Karlsruhe, Germany), Sigma-Aldrich (Buchs, Switzerland) and VWR (Darmstadt, Germany) and used without further purification. Chlorotrityl chloride resin (1.55 mmol/g, 75–150 µm) was obtained from Iris Biotech (Marktredwitz, Germany). Solutions of hydrazine and DBU in DMF each for removal of Dde and Fmoc (only in the case of loading level determination), respectively, were freshly prepared. For 2% (*v*/*v*) DBU/DMF, 200 µL of DBU was mixed with 9.8 mL of DMF. A quantity of 900 µL of hydrazine monohydrate was added to 30 mL of DMF to obtain 2% (*v*/*v*) N_2_H_4_/DMF.

Mass spectra (ESI) were recorded with a Micromass “Quattro LC” instrument (Waters Corporation, Milford, MA, USA) or a Waters Xevo TQ-S mass spectrometer Biotech (Waters Corporation, Milford, MA, USA), each driven by the Mass Lynx software Biotech (Version 4.1, Waters Corporation, Milford, MA, USA).

NMR spectroscopy was carried out using the “UNITY INOVA 400” instrument from Varian (Palo Alto, CA, USA). The ^1^H NMR spectra were recorded at 400 MHz, the ^13^C NMR spectra at 101 MHz and the ^19^F NMR spectra at 376 MHz, and 25 °C in each case. In the case of the tertiary amide **7**, the ^1^H NMR spectrum was additionally recorded at 40, 60 and 70 °C. All compounds were dissolved in appropriate deuterated solvents as specified below and the resulting spectra, except for the ^19^F NMR spectra, were calibrated to the appropriate solvent shifts. The software “MestReNova” (Version: 6.1.1–6384, Santiago de Compostela, Spain) was used to evaluate the spectra.

Purification of compounds **1**–**18** was performed on a semi-preparative HPLC sys-tem (AlphaCrom, Rheinfelden, Switzerland) consisting of the components listed in Table 2. Gradient-mode elution was applied according to Table 3.

### 3.2. Synthetic Methods

The synthetic procedures in the following subsections are described using the example of the synthetic pathway to *N*^1^-FBz-spermidine, and respectively apply to the other polyamine and oxopolyamine derivatives.

#### 3.2.1. Loading of Symmetric Polyamines to the 2-ClTrtCl Resin

Resin attachment of the various diamines and polyamines was carried out in orientation to procedures published in [45,61].

A solution of putrescine (353 mg, 4.00 mmol, 4 eq.) in CH_2_Cl_2_ (4 mL) was added to the 2-ClTrtCl resin (1.55 mmol/g; 645 mg, 1.00 mmol, 1 eq.) preswollen in CH_2_Cl_2_ (5 mL, 30 min) in a filter syringe. A white precipitate formed after addition of the amine solution. The filter syringe was capped and shaken for 16 h at room temperature. Subsequently, the resin was washed with DMF (4 mL, 4 × 1 min) and CH_2_Cl_2_ (4 mL, 4 × 1 min) and treated with a solution of CH_2_Cl_2_/CH_3_OH/DIPEA (17/1/2, 5 mL, 4 × 2 min) for quenching of unreacted chlorotrityl chloride groups. During this treatment, the precipitate mentioned above dissolved. After washing with CH_3_OH, TEA/DMF (1/4), CH_3_OH and CH_2_Cl_2_ (5 mL and 3 × 1 min each), the resin was dried overnight in a desiccator under vacuum.

#### 3.2.2. Assembly of Fmoc-Protected Oxopolyamines

The solid-phase synthesis to construct the Fmoc-protected oxopolyamines was carried out on a CEM automated peptide synthesizer with an integrated microwave reactor (Liberty—12-Channel Automated Peptide Synthesizer, Kamp-Lintfort, Germany). In all cases, the amount of material used for synthesis was 0.5 mmol (1 eq.) of the respective Fmoc-protected oxopolyamine. The preloaded polyamine resin was weighed in based on the maximum possible resin loading (1.55 mmol/g).

The putrescine-loaded resin (323 mg) was transferred into the reaction vessel and swollen in CH_2_Cl_2_/DMF (1/1, 20 mL) for 15 min. Activation and coupling of Fmoc-*β*-Ala-OH was realized by combining 0.45 M HBTU/DMF (4 mL) and 2 M DIPEA/NMP (2 mL). The amino acid to be coupled was present as a 0.2 M solution in DMF, and 10 mL (4 eq.) of this solution was used for the reaction (300 s at 25 W and 75 °C). Subsequently, the *N*^1^-Fmoc-3-oxospermidine-loaded resin was washed with DMF (60 mL in total) and, after completion of the synthesis, repeatedly with ethanol and CH_2_Cl_2_ and dried overnight in a desiccator under vacuum.

In the case of the synthesis of *N*^1^-Fmoc-3,8-dioxospermine, Fmoc cleavage was carried out after coupling of Fmoc-*γ*-Abu-OH by two-fold treatment with a solution of piperidine in DMF (20% *v*/*v*) containing 0.1 M HOBt (15 mL each, 35 W, 30 s and 35 W, 180 s, both at 75 °C). After each treatment, the resin was washed with DMF (5 mL and 85 mL, respectively).

#### 3.2.3. Determination of Resin Loading Levels

Determination of resin loading for *N*^1^-Fmoc-3-oxospermidine, *N*^1^-Fmoc-5-oxospermidine, and *N*^1^-Fmoc-3,8-dioxospermine was performed according to the procedure published in [62]. A determination in duplicate was performed each.

The loaded resin (approximately 5 mg, exact value was noted) was weighed into a filter syringe and preswollen in DMF (2 mL) for 30 min. Subsequently, 2 mL of a 2% DBU solution in DMF was measured using an Eppendorf pipette and soaked into the filter syringe containing the resin. The resulting mixture was shaken for 30 min. The solution was then filtered into a 10 mL graduated flask, the resin was washed several times with CH_3_CN (3 × 1 mL), and all filtrates combined in the graduated flask. After complementing the volume to 10 mL with CH_3_CN in the graduated flask, 2 mL of this solution was measured using an Eppendorf pipette (Hamburg, Germany) and transferred into a 25 mL graduated flask and the volume complemented to 25 mL with CH_3_CN. A reference solution without the resin was prepared accordingly. The extinction of the sample solutions were measured at *λ* = 294 nm using a UV spectrometer (Helios α, Thermo Electron Corporation, Waltham, MA, USA) against the reference solution as blank. The resin loading was calculated by the following Equation (1):

Loading degree (mmol/g)_294 nm_ = (E_294_ × 14.214 μmol)/m_resin_ (mg) (1)

where E_294_ is the measured value of the extinction at 294 nm and m_resin_ is the mass of the resin sample.

The mean value was calculated from the results of the duplicate determination. The loading levels obtained for *N*^1^-Fmoc-3-oxospermidine, *N*^1^-Fmoc-5-oxospermidine, and *N*^1^-Fmoc-3,8-dioxospermine were 0.50–0.70 mmol/g after several syntheses.

Loading levels for the binding of putrescine, cadaverine, and spermine were determined gravimetrically using Equation (2):

Loading degree (mol/g) = (m_2_ − m_1_)/[(MW − 36.461) × m_2_] (2)

where m_1_ = mass (g) of unloaded resin, m_2_ = mass (g) of loaded resin, MW = molar mass (g/mol) of the respective polyamine.

The loading degree obtained for putrescine was 0.90 mmol/g, for cadaverine 1.22 mmol/g, and for spermine 1.05 mmol/g.

#### 3.2.4. Removal of the Fmoc Group

Fmoc cleavage was performed following the procedures published in [45,92]. The *N*^1^-Fmoc-3-oxospermidine-loaded resin (307 mg, 0.20 mmol, 0.64 mmol/g) preswollen in DMF (5 mL, 1 h) was treated with a 20% (*v*/*v*) piperidine solution in DMF (5 mL, 3 × 20 min, 1 × 45 min) and subsequently washed with DMF (5 mL, 4 × 1 min), 5% DIPEA solution in DMF (5 mL, 4 × 1 min), DMF (5 mL, 2 × 1 min), and CH_2_Cl_2_ (5 mL, 4 × 1 min). The 3-oxospermidine resin was dried in a desiccator under vacuum overnight.

#### 3.2.5. Amide Bond Reduction

Reduction with BH_3_/THF was performed according to the procedure published in [45,46]. The 3-oxospermidine-loaded resin (250 mg, 0.20 mmol, Equation (1)) was weighed into a Schlenk vessel and placed under an inert atmosphere by evacuating and purging the vessel with Ar several times. Next, a solution of BH_3_ in THF (1 M, 4.0 mL, 4.00 mmol, 20 eq. per amide bond) was added through a septum to the 3-oxospermidine resin. The mixture was heated to moderate boiling at 65 °C for 40 h under addition of the identical volume of the BH_3_ solution after half of the time. Strong bubble formation occurred during the reaction and the resin rapidly turned white. After the mixture cooled to room temperature, the suspended resin was quickly transferred to a filter syringe using a Pasteur pipette and washed several times with THF. Subsequently, a solution of THF/DIPEA/HOAc (17/1/2, 2.5 mL, 10 mL/g resin) was added to the resin. After brief shaking of the suspension, iodine (609 mg, 2.40 mmol, 12 eq.) was added as a suspension in THF (1 mL) and the resulting reaction mixture was shaken for 4 h. The mixture was then mixed with a solution of DIPEA/HOAc (17/1/2, 2.5 mL, 10 mL/g resin). Subsequently, the resin was washed with THF, TEA/DMF 1/3 CH_3_OH, and CH_2_Cl_2_ (5 mL and 4 × 1 min each), upon which it turned yellow again, and dried overnight in a desiccator under vacuum.

#### 3.2.6. Dde Protection

Dde protection was performed according to [45]. A solution of Dde-OH (73 mg, 0.40 mmol, Equation (2)) in DMF (3.5 mL) was added to the spermidine-loaded resin (0.20 mmol, 1 eq.) preswollen in DMF (5 mL, 30 min). The resulting reaction mixture was shaken overnight at room temperature. Subsequently, the resin was washed with DMF, CH_3_OH, and CH_2_Cl_2_ (5 mL and 4 × 1 min each) and dried overnight in a desiccator under vacuum.

#### 3.2.7. Boc Protection

Boc protection was carried out in orientation to [45]. The *N*^1^-Dde-spermidine loaded resin (0.20 mmol, 1 eq.) was preswollen in CH_2_Cl_2_ (4 mL, 30 min) and a solution of DIPEA (209 µL, 1.20 mmol, 6 eq. per secondary amine) in CH_2_Cl_2_ (2 mL) was added. The suspension was shaken for 1 min and a solution of Boc_2_O (524 mg, 2.4 mmol, 12 eq. per secondary amine) in CH_2_Cl_2_ (2 mL) was subsequently added. The resulting mixture was shaken overnight at room temperature. Subsequently, the resin was washed with DMF, CH_3_OH, and CH_2_Cl_2_ (5 mL and 4 × 1 min each) and dried overnight in a desiccator under vacuum.

#### 3.2.8. Removal of the Dde Group

Dde cleavage was performed according to the procedures published in [45,74]. After preswelling in DMF (4 mL, 1 h), the *N*^4^-Boc-*N*^1^-Dde-spermidine-loaded resin (0.20 mmol) was mixed several times with N_2_H_4_ solution (2% in DMF; 5 mL, 10 × 15 min). The filtrates were each measured at *λ* = 300 nm on the UV spectrometer against the N_2_H_4_ solution as blank until no significant absorbance was detectable anymore. Subsequently, the resin was washed with DMF, CH_3_OH and CH_2_Cl_2_ (5 mL and 4 × 1 min each) and dried overnight in a desiccator under vacuum.

#### 3.2.9. Fluorobenzoylation of Amino Groups

The *N*^4^-Boc-spermidine-loaded resin (0.20 mmol, 1 eq.) was preswollen in CH_2_Cl_2_ (5 mL, 30 min), and then TEA (70 μL, 0.50 mmol, 2.5 eq.) and 4-fluorobenzoyl chloride (59 μL, 0.50 mmol, 2.5 eq.) were added to this suspension. The mixture was shaken for 2 h at room temperature. Subsequently, the resin was washed with DMF, CH_3_OH, and CH_2_Cl_2_ (5 mL each and 4 × 1 min each) and dried overnight in a desiccator under vacuum.

#### 3.2.10. Fluorobenzylation of Amino Groups

Reductive alkylation was performed according to the procedure published in [93]. The polyamine-loaded resin (0.17 mmol, 1 eq.) was preswollen in THF (4 mL, 30 min), and then 4-fluorobenzaldehyde (54 μL, 0.51 mmol, 3 eq.) was added to this suspension. The mixture was shaken for 3.5 h at room temperature. Subsequently, the resin was filtrated and washed with THF (1 × 4 mL) and a solution of sodium triacetoxyborohydride (72 mg, 0.34 mmol, 2 eq.) in THF (3 mL) was added. This mixture was shaken for 3 h at room temperature. Subsequently, the resin was washed with DMF, CH_3_OH, and CH_2_Cl_2_ (5 mL each and 4 × 1 min each) and dried overnight in a desiccator under vacuum.

#### 3.2.11. Cleavage of Polyamines/Oxopolyamines from the Resin

The cleavage was performed following the peptide cleavage procedure reported in [45]. To the dry *N*^1^-FBz-spermidine-loaded resin (0.15 mmol, ¾ of the total amount) was added a cleavage solution of TFA/TES/H_2_O (95/2.5/2.5, 5 mL) and shaken for 2 h. Subsequently, the resin was washed with TFA (2 × 5 mL) and the combined filtrates were evaporated in a nitrogen stream nitrogen until only an oily residue was remaining. The crude product was purified by HPLC (see Section 3.1).

### 3.3. Yields and Analytical Data for Compounds **1**–**18**

Yields were calculated based on the loading levels determined as described in Section 3.2.3.

#### 3.3.1. *N*^1^-(4-Fluorobenzoyl)-putrescine × TFA (**1**)

**^1^H NMR** (DMSO-*d_6_*): *δ*(ppm) = 8.54 (t, ^3^*J* = 5,6 Hz, 1H, NH), 7.91 (dd, ^3^*J*_H,H_ = 8.8 Hz, ^4^*J*_H,F_ = 5.5 Hz, 2H, H–2,6 FBz), 7.69 (broad s, 3H, NH_3_^+^), 7.30 (t, ^3^*J*_H,H_ = ^3^*J*_H,F_ = 8.8 Hz, 2H, H–3,5 FBz), 3.33–3.21 (m, 2H, C*H*_2_NH), 2.87–2.74 (m, 2H, C*H*_2_NH_3_^+^), 1.62–1.49 (m, 4H, 2 CH_2_)

**^13^C NMR** (DMSO-*d_6_*): *δ*(ppm) = 165.13 (CO FBz), 163.79 (d, ^1^*J*_C,F_ = 248.3 Hz, C–4 FBz), 158.09 (q, ^2^*J*_C,F_ = 34.7 Hz, CO TFA anion), 130.99 (d, ^4^*J*_C,F_ = 2.9 Hz, C–1 FBz), 129.71 (d, ^3^*J* = 9,0 Hz, C–2,6 FBz), 115.16 (d, ^2^*J*_C,F_ = 21.7 Hz, C–3,5 FBz), 38.67, 38.48, 26.12, 24.58

**^19^F NMR** (DMSO-*d_6_*): δ(ppm) = −74.84 (s, CF_3_ TFA anion), −110.10– −110.20 (m, FBz)


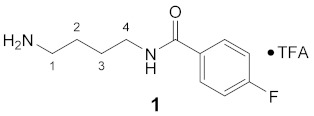



**Yield:**
28 mg (63%), white solid
**MS (ESI+):**
*m/z* = 211.3 ([M + H]^+^), *m/z* = 432.6 ([2M + H]^+^) M (monoisotopic) calculated for C_11_H_15_FN_2_O: 210.12 g/mol

#### 3.3.2. N^1^-(4-Fluorobenzoyl)-cadaverine × TFA (**2**)

**^1^H NMR** (DMSO-*d_6_*): *δ*(ppm) = 8.48 (t, ^3^*J* = 5.5 Hz, 1H, NH), 7.90 (dd, ^3^*J*_H,H_ = 8.9 Hz, ^4^*J*_H,F_ = 5.5 Hz, 2H, H–2,6 FBz), 7.71 (broad s, 3H, NH_3_^+^), 7.29 (t, ^3^*J*_H,H_ = ^3^*J*_H,F_ = 8.9 Hz, 2H, H–3,5 FBz), 3.30–3.21 (m, 2H, H–5), 2.84–2.73 (m, 2H, H–1), 1.61–1.48 (m, 4H, H–2,4), 1.40–1.28 (m, 2H, H–3)

**^13^C NMR** (DMSO-*d_6_*): *δ*(ppm) = 165.05 (CO FBz), 163.75 (d, ^1^*J*_C,F_ = 248.1 Hz, C–4 FBz), 157.97 (q, ^2^*J_C,F_* = 31.8 Hz, CO TFA-Anion), 131.08 (d, ^4^*J* = 2.9 Hz, C–1 FBz), 129.72 (d, ^3^*J*_C,F_ = 8.9 Hz, C–2,6 FBz), 115.12 (d, ^2^*J*_C,F_ = 21.7 Hz, C–3,5 FBz), 38.90, 38.77, 28.56, 26.73, 23.26 (C–3)

**^19^F NMR** (DMSO-d_6_): *δ*(ppm) = −74.21 (s, CF_3_ TFA-Anion), −110.27 (tt, ^3^*J*_F,H_ = 8.9 Hz, ^4^*J*_F,H_ = 5.6 Hz FBz)


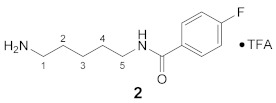



**Yield:**
53 mg (65%), white waxy solid
**MS (ESI+):**
*m*/*z* = 225.4 ([M + H]^+^) M (monoisotopic) calculated for C_12_H_17_FN_2_O: 224.13 g/mol

#### 3.3.3. N^1^-(4-Fluorobenzoyl)-1,6-diaminohexane × TFA (**3**)

**^1^H NMR** (DMSO-*d_6_*): *δ*(ppm) = 8.47 (t, ^3^*J* = 5.4 Hz, 1H, NH), 7.90 (dd, ^3^*J*_H,H_ = 8.8, ^4^*J*_H,F_ = 5.5 Hz, 2H, H–2,6 FBz), 7.69 (broad s, 3H, NH_3_^+^), 7.28 (t, ^3^*J*_H,H_ = ^3^*J*_H,F_ = 8.9 Hz, 2H, H–3,5 FBz), 3.28–3.21 (m, 2H, H–6), 2.82–2.72 (m, 2H, H–1), 1.57–1.47 (m, 4H, H–2,5), 1.37–1.26 (m, 4H, H–2,6)

**^13^C NMR** (DMSO-*d_6_*): *δ*(ppm) = 165.01 (CO FBz), 163.74 (d, ^1^*J*_C,F_ = 248.1 Hz, C–4 FBz), 157.94 (d, ^2^*J* = 32.7 Hz, CO TFA), 131.11 (d, ^4^*J*_C,F_ = 3.0 Hz, C–1 FBz), 129.70 (d, ^3^*J*_C,F_ = 4.5 Hz, C–2,6 FBz), 115.10 (d, ^2^*J*_C,F_ = 21.6 Hz, C–3,5 FBz), 38.78, 28.92, 26.96, 25.96, 25.51

**^19^F NMR** (DMSO-d_6_): *δ*(ppm) = −74.40 (s, CF_3_), −110.31 (tt, ^3^*J*_F,H_ = 8.9 Hz, ^4^*J*_F,H_ = 5.5 Hz, FBz)


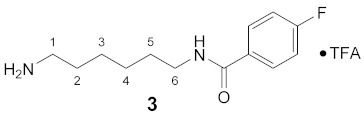



**Yield:**
44 mg (84%), yellow waxy solid
**MS (ESI+):**
*m*/*z* = 238.92 ([M + H]^+^) M (monoisotopic) calculated for C_13_H_19_FN_2_O: 238.15 g/mol

#### 3.3.4. *N*^1^-(4-Fluorobenzoyl)-1,7-diaminoheptane × TFA (**4**)

**^1^H NMR** (DMSO-*d_6_*): *δ*(ppm) = 8.47 (t, ^3^*J* =5.3 Hz, 1H, NH), 7.90 (dd, ^3^*J*_H,H_ = 8.8, ^4^*J*_H,F_ = 5.6 Hz, 2H, H–10), 7.68 (broad s, 3H, NH_3_^+^), 7.28 (t, ^3^*J*_H,H_ = ^3^*J*_H,F_ = 8.9 Hz, 2H, H–11), 3.28–3.20 (m, 2H, H–7), 2.82–2.71 (m, 2H, H–1), 1.56–1.46 (m, 4H, H–2,6), 1.34–1.22 (m, 6H, H–3,4,5)

**^13^C NMR** (DMSO-*d_6_*): *δ*(ppm) = 165.26 (CO FBz), 163.88 (d, ^1^*J*_C,F_ = 248.1 Hz, C–4 FBz), 158.14 (d, ^2^*J* = 30.8 Hz, CO TFA), 131.20 (d, ^4^*J*_C,F_ = 3.0 Hz, C–1 FBz), 129.83 (d, ^3^*J*_C,F_ = 9.0 Hz, C–2,6 FBz), 115.28 (d, ^2^*J*_C,F_ = 21.7 Hz, C–3,5 FBz), 38.98, 29.10, 28.35, 27.06, 26.38, 25.86, Signal for 1×CH_2_ is not visible.

**^19^F NMR** (DMSO-d_6_): *δ*(ppm) = −74.09 (s, CF_3_), −110.33 (tt, ^3^*J*_F,H_ = 8.9 Hz, ^4^*J*_F,H_ = 5.6 Hz, FBz)


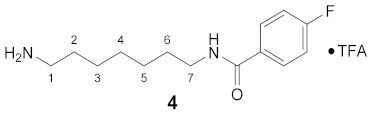



**Yield:**
37 mg (64%), white solid
**MS (ESI+):**
*m*/*z* = 252.82 ([M + H]^+^) M (monoisotopic) calculated for C_14_H_21_FN_2_O: 252.16 g/mol

#### 3.3.5. *N*^1^-(4-Fluorobenzoyl)-1,8-diaminooctane × TFA (**5**)

**^1^H NMR** (DMSO-*d_6_*): *δ*(ppm) = 8.46 (t, ^3^*J* = 5.4 Hz, 1H, NH), 7.90 (dd, ^3^*J*_H,H_ = 8.9, ^4^*J*_H,F_ = 5.5 Hz, 2H, H–11), 7.68 (broad s, 3H, NH_3_^+^), 7.28 (t, ^3^*J*_H,H_ = ^3^*J*_H,F_ = 8.9 Hz, 2H, H–12), 3.27–3.19 (m, 2H, H–8), 2.81–2.71 (m, 2H, H–1), 1.56–1.46 (m, 4H, H–2,7), 1.35–1.21 (m, 8H, H–3,4,5,6)

**^13^C NMR** (DMSO-*d_6_*): *δ*(ppm) = 165.09 (C–9), 163.79 (d, ^1^*J*_C,F_ = 248.1 Hz, C–4 FBz), 157.96 (d, ^2^*J* = 30,.5 Hz, CO TFA), 131.16 (d, ^4^*J*_C,F_ = 2.9 Hz, C–1 FBz), 129.75 (d, ^3^*J*_C,F_ = 9.0 Hz, C–2,6 FBz), 115.18 (d, ^2^*J*_C,F_ = 21.7 Hz, C–3,5 FBz), 39.25, 29.10, 28.59, 28.50, 27.01, 26.40, 25.78, Signal for 1 × CH_2_ is not visible.

**^19^F NMR** (DMSO-d_6_): *δ*(ppm) = −73.99 (s, CF_3_), −110.34 (tt, ^3^*J*_F,H_ = 8.9 Hz, ^4^*J*_F,H_ = 5.5 Hz, FBz)


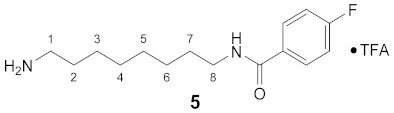



**Yield:**
35 mg (64%), white solid
**MS (ESI+):**
*m*/*z* = 266.83 ([M + H]^+^) M (monoisotopic) calculated for C_15_H_23_FN_2_O: 266.16 g/mol

#### 3.3.6. *N*^1^-(4-Fluorobenzoyl)-3-oxospermidine × TFA (**6**)

**^1^H NMR** (DMSO-*d_6_*): *δ*(ppm) = 8.54 (t, ^3^*J* = 5.5 Hz, 1H, H–9), 7.94 (t, ^3^*J* = 5,6 Hz, 1H, H–5), 7.89 (dd, ^3^*J*_H,H_ = 8.9 Hz, ^4^*J*_H,F_ = 5.5 Hz, 2H, H–2,6 FBz), 7.67 (broad s, 3H, NH_3_^+^), 7.29 (t, ^3^*J*_H,H_ = ^3^*J*_H,F_ = 8.9 Hz, 2H, H–3,5 FBz), 3.48–3.39 (m, 2H, H–8), 3.10–3.01 (m, 2H, H–4), 2.82–2.71 (m, 2H, H–1), 2.36 (t, ^3^*J* = 7,3 Hz, 2H, H–7), 1.55–1.37 (m, 4H, H–2,3)

**^13^C NMR** (DMSO-*d_6_*): *δ*(ppm) = 170.23 (C–6), 165.10 (C–10), 163.78 (d, ^1^*J*_C,F_ = 248.2 Hz, C–4 FBz), 130.90 (d, ^4^*J*_C,F_ = 2.9 Hz, C–1 FBz), 129.71 (d, ^3^*J*_C,F_ = 9.0 Hz, C–2,6 FBz), 115.15 (d, ^2^*J*_C,F_ = 21,7 Hz, C–3,5 FBz), 38.57, 37.70, 36.22, 35.37, 26.11, 24.47

**^19^F NMR** (DMSO-d_6_): *δ*(ppm) = −74.30 (s, CF_3_ TFA-Anion), −110.11 (tt, ^3^*J*_F,H_ = 8.9 Hz, ^4^*J*_F,H_ = 5.6 Hz FBz)


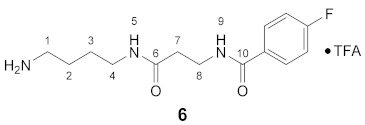



**Yield:**
26 mg (88%), white waxy solid
**MS (ESI+):**
*m*/*z* = 282.5 ([M + H]^+^), *m*/*z* = 304.5 ([M + Na]^+^), *m*/*z* = 563.7 ([2M + H]^+^)M (monoisotopic) calculated for C_14_H_20_FN_3_O_2_: 281.15 g/mol

#### 3.3.7. *N*^8^-(4-Fluorobenzoyl)-5-oxospermidine × TFA (**7**)

**^1^H NMR** (DMSO-*d_6_*): *δ*(ppm) = 8.50 (t, ^3^*J* = 5,4 Hz, 1H, H–9), 7.98 (t, ^3^*J* = 5.8 Hz, 1H, H–4), 7.91 (dd, ^3^*J*_H,H_ = 8.9 Hz, ^4^*J*_H,F_ = 5.5 Hz, 2H, H–2,6 FBz), 7.69 (broad s, 3H, NH_3_^+^), 7.29 (t, ^3^*J*_H,H_ = ^3^*J*_H,F_ = 8.9 Hz, 2H, H–3,5 FBz), 3.28–3.21 (m, 2H, C*H*_2_NH), 3.14–3.06 (m, 2H, C*H*_2_NH), 2.82–2.72 (m, 2H, H–1), 2.14 (t, ^3^*J* = 7.5 Hz, 2H, H–6), 1.79–1.70 (m, 2H, CH_2_), 1.70–1.61 (m, 2H, CH_2_)

**^13^C NMR** (DMSO-*d_6_*): *δ*(ppm) = 172.32 (C–5), 165.08 (C–10), 163.76 (d, ^1^*J*_C,F_ = 248.1 Hz, C–4 FBz), 157.98 (q, ^2^*J*_C,F_ = 33.3 Hz, CO TFA-Anion), 131.02 (d, ^4^*J*_C,F_ = 3.0 Hz, C–1 FBz), 129.73 (d, ^3^*J*_C,F_ = 8.9 Hz, C–2,6 FBz), 115.12 (d, ^2^*J*_C,F_ = 21.7 Hz, C–3,5 FBz), 38.90, 36.80, 35.47, 32.86, 27.51, 25.32

**^19^F NMR** (DMSO-d_6_): *δ*(ppm) = −74.49 (s, CF_3_ TFA-Anion), −110.22 (tt, ^3^*J*_F,H_ = 8.9 Hz, ^4^*J*_F,H_ = 5.5 Hz FBz)


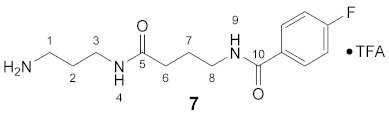



**Yield:**
39 mg (quantitative containing residual TFA), colorless viscous oil
**MS (ESI+):**
*m*/*z* = 282.4 ([M + H]^+^), *m*/*z* = 304.4 ([M + Na]^+^), *m*/*z* = 563.7 ([2M + H]^+^) M (monoisotopic) calculated for C_14_H_20_FN_3_O_2_: 281.15 g/mol

#### 3.3.8. *N*^1^-(4-Fluorobenzoyl)-3,8-dioxospermine × TFA (**8**)

**^1^H NMR** (DMSO-*d_6_*): *δ*(ppm) = 8.54 (t, *J* = 5.6 Hz, 1H, H–10), 7.97–7.85 (m, 4H, H–2,6 FBz and H–1,6), 7.68 (broad s, 3H, NH_3_^+^), 7,28 (t, ^3^*J*_H,H_ = ^3^*J*_H,F_ = 8.9 Hz, 2H, H–3,5 FBz), 3.48–3.40 (m, 2H, H–9), 3.14–3.07 (m, 2H, C*H*_2_NH), 3.06–2.98 (m, 2H, C*H*_2_NH), 2.82–2.72 (m, 2H, H–14), 2,35 (t, ^3^*J* = 7.3 Hz, 2H, H–8), 2.07 (t, ^3^*J* = 7.5 Hz, 2H, H–3), 1.70–1.56 (m, 4H, H–4,13)

**^13^C NMR** (DMSO-*d_6_*): *δ*(ppm) = 172.28 (CO), 170.22 (CO), 165.13 (C–11), 163.78 (d, ^1^*J*_C,F_ = 248.2 Hz, C–4 FBz), 158.05 (q, ^2^*J*_C,F_ = 34.3 Hz, CO TFA-Anion), 130.94 (d, ^4^*J*_C,F_ = 2.9 Hz, C–1 FBz), 129.72 (d, ^3^*J*_C,F_ = 9.0 Hz, C–2,6 FBz), 115.15 (d, ^2^*J*_C,F_ = 21.7 Hz, C–3,5 FBz), 38.07, 36.79, 36.21, 35.46, 35.38, 32.77, 27.52, 25.41

**^19^F NMR** (DMSO-d_6_): *δ*(ppm) = −74.74 (s, CF_3_ TFA-Anion), −110.13 (tt, ^3^*J*_F,H_ = 8.9 Hz, ^4^*J*_F,H_ = 5.5 Hz FBz)


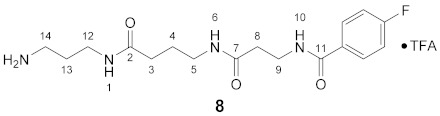



**Yield:**
69 mg (89%), colorless viscous oil
**MS (ESI+):**
*m*/*z* = 353.5 ([M + H]^+^) M (monoisotopic) calculated for C_17_H_25_FN_4_O_3_: 352.19 g/mol

#### 3.3.9. *N*^1^-(4-Fluorobenzoyl)-spermidine × 2TFA (**9**)

**^1^H NMR** (DMSO-*d_6_*): *δ*(ppm) = 8.68 (t, ^3^*J* = 5.8 Hz, 1H, NH), 8.52 (broad s, 2H, NH_2_^+^), 7,92 (dd, ^3^*J*_H,H_ = 8.7 Hz, ^4^*J*_H,F_ = 5.5 Hz, 2H, H–2,6 FBz), 7.80 (broad s, 3H, NH_3_^+^), 7.31 (t, ^3^*J*_H,H_ = ^3^*J*_H,F_ = 8.8 Hz, 2H, H–3,5 FBz), 3.38–3.28 (m, 2H, H–7), 2.99–2.87 (m, 4H, 2 CH_2_), 2.86–2.75 (m, 2H, CH_2_), 1.90–1.79 (m, 2H, H–6), 1.66–1.51 (m, 4H, H–2,3)

**^13^C NMR** (DMSO-*d_6_*): *δ*(ppm) = 165.55 (CO FBz), 163.89 (d, ^1^*J*_C,F_ = 250.5 Hz, C–4 FBz), 158.17 (q, ^2^*J*_C,F_ = 35.4 Hz, CO TFA anion), 130.67 (d, ^4^*J*_C,F_ = 3.0 Hz, C–1 FBz), 129.80 (d, ^3^*J*_C,F_ = 9.0 Hz, C–2,6 FBz), 115.22 (d, ^2^*J*_C,F_ = 21.8 Hz, C–3,5 FBz), 46.10, 44.77, 38.23, 36.41, 26.05, 24.15, 22.62

**^19^F NMR** (DMSO-d_6_): *δ*(ppm) = −74.75 (s, CF_3_ TFA anion), −109.79–−109.88 (m, FBz)


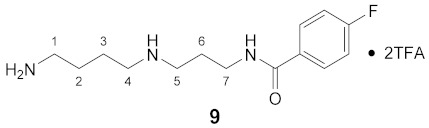



**Yield:**
28 mg (38%), pale yellow viscous oil
**MS (ESI+):**
*m*/*z* = 123.3 ([FBz]^+^), *m*/*z* = 146.4 ([M – FBz + 2H]^+^), *m*/*z* =268.5 ([M + H]^+^) M (monoisotopic) calculated for C_14_H_22_FN_3_O: 267.17 g/mol

#### 3.3.10. *N*^4^-(4-Fluorobenzoyl)-spermidine × 2TFA (**10**)

**^1^H NMR** (DMSO-*d_6_* 25 °C): *δ*(ppm) = 7.79 (broad s, 6H, 2 NH_3_^+^), 7.43 (broad s, 2H, H–2,6 FBz), 7.28 (t, ^3^*J*_H,H_ = ^3^*J*_H,F_ = 8.9 Hz, 2H, H–3,5 FBz), 3.17 (broad s, 2H, CH_2_N), 2.85 (broad s, 2H, C*H*_2_NH_3_^+^), 2.64 (broad s, 2H, C*H*_2_NH_3_^+^), 1.94–1.21 (m, 6H, H–2,5,6)

**^1^H-NMR** (DMSO-*d_6_* 70 °C): *δ*(ppm) = 7.74 (s, 6H, 2 NH_3_^+^), 7.43 (dd, ^3^*J*_H,H_ = 8.7 Hz, ^4^*J*_H,F_ = 5.5 Hz, 2H, H–2,6 FBz), 7.26 (t, ^3^*J*_H,H_ = ^3^*J*_H,F_ = 8.9 Hz, 2H, H–3,5 FBz), 3.40 (broad s, 2H, C*H*_2_NH_3_^+^), 2,69 (broad s, 4H, H–3,4), 1.91–1.79 (m, 2H, CH_2_), 1.64–1.52 (m, 2H, CH_2_), 1.45 (s, 2H, CH_2_)

**^13^C-NMR** (DMSO-*d_6_*): *δ*(ppm) = 170.15 (CO FBz), 162.28 (d, ^1^*J*_C,F_ = 246.3 Hz, C–4 FBz), 158,02 (q, ^2^*J*_C,F_ = 31.3 Hz, CO TFA anion), 133,08 (broad s, C–1 FBz), 128.81 (broad s, C–2,6 FBz), 115.44 (d, ^2^*J* = 21.6 Hz, C–3,5 FBz), 48.19, 41.28, 38.39, 36.76, 25.45, 24.93, 24.13

**^19^F-NMR** (DMSO-d_6_): *δ*(ppm) = −74.10 (s, CF_3_ TFA anion), −111.87– −112.17 (m, FBz)


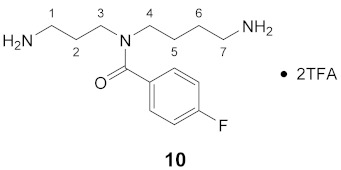



**Yield:**
21 mg (35%), colorless viscous oil
**MS (ESI+):**
*m*/*z* = 146.7 ([M – FBz + 2H]^+^), *m*/*z* = 268.4 ([M + H]^+^), *m*/*z* = 535.7 ([2M + H]^+^) M (monoisotopic) calculated for C_14_H_22_FN_3_O: 267.17 g/mol

#### 3.3.11. *N*^8^-(4-Fluorobenzoyl)-spermidine × 2TFA (**11**)

**^1^H NMR** (DMSO-*d_6_*): *δ*(ppm) = 8.66–8.45 (m, 3H, NH and NH_2_^+^), 7.95–7.80 (m, 5H, H–2,6 FBz and NH_3_^+^), 7.30 (t, ^3^*J*_H,H_ = ^3^*J*_H,F_ = 8.9 Hz, 2H, H–3,5 FBz), 3.36–3.23 (m, 2H, H–7), 3.02–2.79 (m, 6H, H–1,3,4), 1.93–1.75 (m, 2H, H–2), 1.68–1.50 (m, 4H, H–5,6)

**^13^C NMR** (DMSO-*d_6_*): *δ*(ppm) = 165.16 (CO FBz), 163.79 (d, ^1^*J*_C,F_ = 248.2 Hz, C–4 FBz), 158,11 (q, ^2^*J*_C,F_ = 32.2 Hz, CO TFA anion), 130.95 (d, ^4^*J*_C,F_ = 2.9 Hz, C–1 FBz), 129.71 (d, ^3^*J*_C,F_ = 9.0 Hz, C–2,6 FBz), 115.16 (d, ^2^*J*_C,F_ = 21.7 Hz, C–3,5 FBz), 46.63, 43.92, 38.47, 36.19, 26.22, 23.79, 23.08

**^19^F NMR** (DMSO-d_6_): *δ*(ppm) = −74.19 (s, CF_3_ TFA anion), −110.12 (tt, ^3^*J*_F,H_ = 8.8 Hz, ^4^*J*_F,H_ = 5.5 Hz FBz)


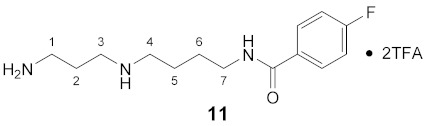



**Yield:**
14 mg (29%), pale yellow waxy solid
**MS (ESI+):**
*m*/*z* = 123.3 ([FBz]^+^), *m*/*z* = 146.7 ([M – FBz + 2H]^+^), *m*/*z* = 268.5 ([M + H]^+^) M (monoisotopic) calculated for C_14_H_22_FN_3_O: 267.17 g/mol

#### 3.3.12. *N*^1^-(4-Fluorobenzoyl)-spermine × 3TFA (**12**)

**^1^H NMR** (DMSO-*d_6_*): *δ*(ppm) = 8.80 (broad s, 2H, NH_2_^+^), 8.68 (t, ^3^*J* = 5.8 Hz, 1H, NH), 8,63 (broad s, 2H, NH_2_^+^), 8.01–7.88 (m, 5H, H–2,6 FBz and NH_3_^+^), 7,31 (t, ^3^*J*_H,H_ *=* ^3^*J*_H,F_ = 8.9 Hz, 2H, H–3,5 FBz), 3.37–3.29 (m, 2H, H–7), 3.02–2.83 (m, 10H, H–1,4,5,8,10), 1.94–1.80 (m, 4H, H–6,9), 1.68–1.57 (m, 4H, H–2,3)

**^13^C NMR** (DMSO-d_6_): *δ*(ppm) = 165.54 (CO), 163.89 (d, ^1^*J*_C,F_ = 249.5 Hz, C–4 FBz), 158.32 (q, ^2^*J*_C,F_ = 31.6 Hz, CO TFA anion), 130.68 (d, ^4^*J*_C,F_ = 2.9 Hz, C–1 FBz), 129.80 (d, ^3^J_C,F_ = 9.0 Hz, C–2,6 FBz), 115.21 (d, ^2^*J*_C,F_ = 21.8 Hz, C–3,5 FBz), 46.12, 46.07, 44.76, 43.88, 36.41, 36.19, 26.04, 23.78, 22.70, 22.64


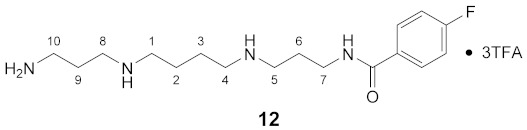



**Yield:**
16 mg (10%), white solid
**MS (ESI+):**
*m*/*z* = 102.3 ([M – FBz + 3H]^2^^+^), *m/z* = 123.1 ([FBz]^+^), *m*/*z* = 163.4 ([M + 2H]^2^^+^), *m/z* = 203.4 ([M – FBz + 2H]^+^), *m*/*z* = 325.5 ([M + H]^+^) M (monoisotopic) calculated for C_17_H_29_FN_4_O: 324.23 g/mol

#### 3.3.13. *N*^1^-(4-Fluorobenzyl)-putrescine × 2TFA (**13**)

**^1^H NMR** (DMSO-*d_6_*): *δ*(ppm) = 8.98 (broad s, 2H, NH_2_^+^), 7.83 (broad s, 3H, NH_3_^+^), 7.54 (dd, ^3^*J*_H,H_ = 8.6, ^4^*J*_H,F_ = 5.5 Hz, 2H, H–2,6 FBn), 7.29 (t, ^3^*J*_H,H_ = ^3^*J*_H,F_ = 8.9 Hz, 2H, H–3,5 FBn), 4.14 (s, 2H, H–5), 2.93 (broad s, 2H), 2.80 (broad s, 2H), 1.69–1.52 (m, 4H, H–2,3)

**^13^C NMR** (DMSO-*d_6_*): *δ*(ppm) = 162.48 (d, ^1^*J*_C,F_ = 245.5 Hz, C–4 FBn), 158.23 (q, ^2^*J* = 31.2 Hz, CO TFA), 132.39 (d, ^3^*J*_C,F_ = 8.6 Hz, C–2,6 FBn), 128.27 (d, ^4^*J*_C,F_ = 3.1 Hz, C–1 FBn), 115.67 (d, ^2^*J*_C,F_ = 21.6 Hz, C–3,5 FBn), 49.27, 45.91, 38.32, 24.25, 22.54

**^19^F NMR** (DMSO-d_6_): *δ*(ppm) = −74.05 (s, CF_3_), −113.11– −113.20 (m, FBn)

^1^H- and ^13^C-NMR data are in agreement to those reported in literature [60].


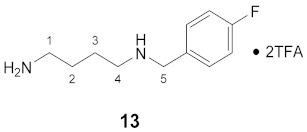



**Yield:**
55 mg (40%), white solid
**MS (ESI+):**
*m*/*z* = 196.70 ([M + H]^+^) M (monoisotopic) calculated for C_11_H_17_FN_2_: 196.14 g/mol

#### 3.3.14. *N*^1^-(4-Fluorobenzyl)-cadaverine × 2TFA (**14**)

**^1^H NMR** (DMSO-*d_6_*): *δ*(ppm) = 8.96 (broad s, 2H, NH_2_^+^), 7.80 (broad s, 3H, NH_3_^+^), 7.54 (dd, ^3^*J*_H,H_ = 8.7, ^4^*J*_H,F_ = 5.5 Hz, 2H, H–2,6 FBn), 7.29 (t, ^3^*J*_H,H_ = ^3^*J*_H,F_ = 8.9 Hz, 2H, H–3,5 FBn), 4.15 (t, ^3^*J*_H,H_ = 5.4 Hz, 2H, H–6), 2.93–2.83 (m, 2H), 2.81–2.72 (m, 2H), 1.66–1.47 (m, 4H), 1.38–1.28 (m, 2H)

**^13^C NMR** (DMSO-*d_6_*): *δ*(ppm) = 162.39 (d, ^1^*J*_C,F_ = 245.4 Hz, C–4 FBn), 158.09 (q, ^2^*J* = 31.1 Hz, CO TFA), 132.29 (d, ^3^*J*_C,F_ = 8.5 Hz, C–2,6 FBn), 128.31 (d, ^4^*J*_C,F_ = 3.1 Hz, C–1 FBn), 115.58 (d, ^2^*J*_C,F_ = 21.5 Hz, C–3,5 FBn), 49.13, 46.13, 38.50, 26.43, 24.81, 22.82

**^19^F NMR** (DMSO-d_6_): *δ*(ppm) = −73.74 (s, CF_3_), −112.80 (tt, ^3^*J*_F,H_ = 9.0 Hz, ^4^*J*_F,H_ = 5.4 Hz, FBn)


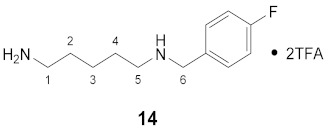



**Yield:**
27 mg (36%), colorless solid
**MS (ESI+):**
*m*/*z* = 210.81 ([M + H]^+^) M (monoisotopic) calculated for C_12_H_19_FN_2_: 210.15 g/mol

#### 3.3.15. *N*^1^-(4-Fluorobenzyl)-1,6-diaminohexane × 2TFA (**15**)

**^1^H NMR** (DMSO-*d_6_*): *δ*(ppm) = 8.91 (broad s, 2H, NH_2_^+^), 7.77 (broad s, 3H, NH_3_^+^), 7.54 (dd, ^3^*J*_H,H_ = 8.6, ^4^*J*_H,F_ = 5.5 Hz, 2H, H–2,6 FBn), 7.29 (t, ^3^*J*_H,H_ = ^3^*J*_H,F_ = 8.9 Hz, 2H, H–3,5 FBn), 4.14 (s, 2H, H–7), 2.88 (broad s, 2H), 2.82–2.71 (m, 2H), 1.64–1.46 (m, 4H, H–2,5), 1.34–1.23 (m, 4H, H–3,4)

**^13^C NMR** (DMSO-*d_6_*): *δ*(ppm) = 162.44 (d, ^1^*J*_C,F_ = 245.5 Hz, C–4 FBn), 158.10 (q, ^2^*J* = 31.8 Hz, CO TFA), 132.35 (d, ^3^*J*_C,F_ = 8.6 Hz, C–2,6 FBn), 128.32 (d, ^4^*J*_C,F_ = 3.1 Hz, C–1 FBn), 115.63 (d, ^2^*J*_C,F_ = 21.6 Hz, C–3,5 FBn), 49.26, 46.44, 38.73, 26.86, 25.55, 25.39, 25.30

**^19^F NMR** (DMSO-d_6_): *δ*(ppm) = −74.07 (s, CF_3_), −113.15– −113.24 (m, FBn)


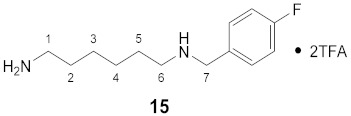



**Yield:**
15 mg (20%), white, waxy solid
**MS (ESI+):**
*m*/*z* = 224.91 ([M + H]^+^) M (monoisotopic) calculated for C_13_H_21_FN_2_: 224.17 g/mol

#### 3.3.16. *N*^1^-(4-Fluorobenzyl)-1,7-diaminoheptane × 2TFA (**16**)

**^1^H NMR** (DMSO-*d_6_*): *δ*(ppm) = 8.89 (broad s, 2H, NH_2_^+^), 7.76 (broad s, 3H, NH_3_^+^), 7.54 (dd, ^3^*J*_H,H_ = 8.3, ^4^*J*_H,F_ = 5.7 Hz, 2H, H–2,6 FBn), 7.29 (t, ^3^*J*_H,H_ = ^3^*J*_H,F_ = 8.8 Hz, 2H, H–3,5 FBn), 4.14 (t, ^3^*J*_H,H_ = 5.2 Hz, 2H, H–8), 2.94–2.84 (m, 2H), 2.81–2.71 (m, 2H), 1.64–1.47 (m, 4H), 1.27 (broad s, 6H)

**^13^C NMR** (DMSO-*d_6_*): *δ*(ppm) = 132.29 (d, ^3^*J*_C,F_ = 8.6 Hz, C–2,6 FBn), 128.31 (d, ^4^*J*_C,F_ = 3.1 Hz, C–1 FBn), 115.57 (d, ^2^*J*_C,F_ = 21.6 Hz, C–3,5 FBn), 49.16, 46.39, 38.74, 28.01, 26.89, 25.79, 25.60, 25.26. Signals for C-4 FBn and CO TFA are not visible.

**^19^F NMR** (DMSO-d_6_): *δ*(ppm) = −73.71 (s, CF_3_), −112.80 (m, FBn)


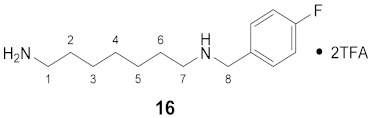



**Yield:**
11 mg (14%), colorless oil
**MS (ESI+):**
*m*/*z* = 238.92 ([M + H]^+^) M (monoisotopic) calculated for C_14_H_23_FN_2_: 238.18 g/mol

#### 3.3.17. *N*^1^-(4-Fluorobenzyl)-1,7-diaminooctane × 2TFA (**17**)

**^1^H NMR** (DMSO-*d_6_*): *δ*(ppm) = 8.90 (broad s, 2H, NH_2_^+^), 7.75 (broad s, 3H, NH_3_^+^), 7.54 (dd, ^3^*J*_H,H_ = 8.6, ^4^*J*_H,F_ = 5.5 Hz, 2H, H–2,6 FBn), 7.29 (t, ^3^*J*_H,H_ = ^3^*J*_H,F_ = 8.8 Hz, 2H, H–3,5 FBn), 4.14 (t, ^3^*J*_H,H_ = 5.6 Hz, 2H, H–9), 2.93–2.83 (m, 2H), 2.81–2.71 (m, 2H), 1.64–1.46 (m, 4H, H–2,7), 1.34–1.23 (broad s, 8H, H–3,4,5,6)

**^13^C NMR** (DMSO-*d_6_*): *δ*(ppm) = 162.38 (d, ^1^*J*_C,F_ = 245.4 Hz, C–4 FBn), 158.04 (q, ^2^*J* = 31.7 Hz, CO TFA), 132.29 (d, ^3^*J*_C,F_ = 8.5 Hz, C–2,6 FBn), 128.32 (d, ^4^*J*_C,F_ = 3.2 Hz, C–1 FBn), 115.56 (d, ^2^*J*_C,F_ = 21.6 Hz, C–3,5 FBn), 49.15, 46.43, 38.78, 28,.30, 28.28, 26.96, 25.83, 25.67, 25.29

**^19^F NMR** (DMSO-d_6_): *δ*(ppm) = −73.75 (s, CF_3_), −112.81 (tt, ^3^*J*_F,H_ = 9.0 Hz, ^4^*J*_F,H_ = 5.5 Hz, FBn)


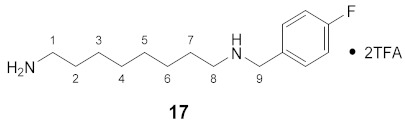



**Yield:**
22 mg (28%), white, waxy solid
**MS (ESI+):**
*m*/*z* = 252.92 ([M + H]^+^) M (monoisotopic) calculated for C_15_H_25_FN_2_: 252.20 g/mol

#### 3.3.18. *N*^1^-(4-Fluorobenzyl)-spermine × 4TFA (**18**)

**^1^H NMR** (DMSO-*d_6_*): *δ*(ppm) = 7.58–7.50 (m, 2H, H-2,6 FBn), 7.29 (t, ^3^*J*_H,H_ = 8.8 Hz, 2H, H-3,5 FBn), 4.14 (s, 2H, H-11), 3.06–2.83 (m, 12H, H-1,4,5,7,8,10), 2.04–1.83 (m, 4H, H-6,9), 1.62 (broad s, 4H, H-2,3). Signals for primary and secondary amino groups are not clearly visible.

**^13^C NMR** (DMSO-d_6_): *δ*(ppm) = 163.59, 158.26 (d, ^2^*J*_C,F_ = 31.5 Hz, CO TFA anion), 132.24 (d, ^3^*J*_C,F_ = 8.2 Hz, C-2,6 FBn), 115.56 (q, ^2^*J*_C,F_ = 21.4 Hz, C-3,5 FBn), 49.34, 46.13, 43.96, 43.88, 43.74, 43.70, 36.19, 23.80, 22.65, 22.49, 22.20.


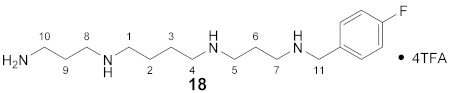



**Yield:**
11 mg (14%), white solid
**MS (ESI+):**
*m*/*z* = 311.06 ([M + H]^+^) M (monoisotopic) calculated for C_17_H_31_FN_4_: 310.25 g/mol

## 4. Conclusions

Within this work, the solid-phase synthetic access to mono-fluorobenzoylated diamines and polyamines was established. The site-selective functionalization of the polyamines spermidine and spermine was achieved by employing Dde for selective protection of the primary amino group. The isomeric mono-*N*-(4-fluorobenzoyl)-spermidines were synthesized by assembling the triamine skeleton by amide bond formation and subsequent reduction with borane in combination with the mentioned protecting group strategy. The compatibility of this approach with radiolabeling by ^18^F-fluorobenzoylation was demonstrated for the case of *N*^1^-[^18^F]FBz-cadaverine within a preliminary pilot experiment. Solid-phase synthesis of 4-fluorobenzylated derivatives was performed for selected diamines and spermine. Based on the results of this study, the radiosynthesis of ^18^F-fluorobenzoylated and ^18^F-fluorobenzylated diamines and polyamines can be established, which in turn will enable their radiopharmacological evaluation in small-animal PET imaging experiments.

## Figures and Tables

**Table 1:** Amounts and yields of the purified polyamine derivatives **1**–**18**.

Compound	Name	Mass (mg)	Yield (%) ^1^
4-Fluorobenzoylated (FBz) Diamines and Polyamines			
**1**	*N*^1^-FBz-putrescine × TFA	28	63
**2**	*N*^1^-FBz-cadaverine × TFA	53	65
**3**	*N*^1^-FBz-1,6-diaminohexane × TFA	44	84
**4**	*N*^1^-FBz-1,7-diaminoheptane × TFA	37	64
**5**	*N*^1^-FBz-1,8-diaminooctane × TFA	35	64
**6**	*N*^1^-FBz-3-oxospermidine × TFA	26	88
**7**	*N*^8^-FBz-5-oxospermidine × TFA	39	>100 ^2^
**8**	*N*^1^-FBz-3,8-dioxospermine × TFA	69	89
**9**	*N*^1^-FBz-spermidine × 2TFA	28	38
**10**	*N*^4^-FBz-spermidine × 2TFA	21	35
**11**	*N*^8^-FBz-spermidine × 2TFA	14	29
**12**	*N*^1^-FBz-spermine × 3TFA	16	10
4-Fluorobenzylated (FBn) Diamines and Polyamines			
**13**	*N*^1^-FBn-putrescine × 2TFA	55	40
**14**	*N*^1^-FBn-cadaverine × 2TFA	27	36
**15**	*N*^1^-FBn-1,6-diaminohexane × 2TFA	15	20
**16**	*N*^1^-FBn-1,7-diaminoheptane × 2TFA	11	14
**17**	*N*^1^-FBn-1,8-diaminooctane × 2TFA	22	28
**18**	*N*^1^-FBn-spermine × 4TFA	11	14

^1^ Calculated based on loading levels of the diamines, spermine, *N*^1^-Fmoc-3-oxospermidine, *N*^8^-Fmoc-5-oxospermidine, and *N*^1^-Fmoc-3,8-dioxospermine. ^2^ Contains excessive TFA.

**Table 2:** Components and parameters of the semi-preparative HPLC system.

Pump (2x):	Varian, PrepStar 218 Solvent Delivery Module
UV/VIS detector:	Varian, ProStar 325
wave length:	254 nm
Software:	AlphaCrom, Star
stationary phase:	Varian, Dynamax 250 × 21.4 mm, Microsorb 60-8 C18
eluent:	A: 0.1% TFA in Wasser B: 0.1% TFA in CH_3_CN
elution mode:	Gradient (Table 3)
flow rate:	10 mL/min

**Table 3:** Elution gradients for HPLC purification of compounds **1–18**.

t (min)	A (%)	B (%)
0–7	90	10
7–22	20	90
22–30	20	90
30–31	90	10
31–35	90	10

## Data Availability

The data presented in this study are available in Supplementary Material.

## Supplementary Materials

Figure S1: ESI(+) mass spectrum after reduction of *N*^1^-Boc-3-oxospermidine, Figure S2: ESI(+) mass spectrum after fluorobenzoylation to *N*^1^-Dde-*N*^4^-FBz-spermidine, Figure S3: ^1^H NMR spectrum of *N*^1^,*N*^1^-di(4-fluorobenzoyl)-1,6-diaminohexane×TFA, Figures S4–S39: ^1^H and ^13^C NMR spectra of all final products.

## Sample Availability

Samples of the final compounds are available upon request from the authors, depending on the temporal availability.
