# Supplementary material for: Solid-Phase Synthesis of Selectively Mono-Fluorobenz(o)ylated Polyamines as a Basis for the Development of 18F-Labeled Radiotracers
Source: Molecules. 2021 Nov 20;26(22):7012. doi: 10.3390/molecules26227012 (PMC8625420; doi:10.3390/molecules26227012)

Supplementary Information

Solid-phase synthesis of selectively mono-fluorobenz(o)ylated polyamines towards the development of radiotracers for tumor imaging

Robert Wodtke [1],* Jens Pietzsch [2] and Reik Löser [2,*]

Institute of Radiopharmaceutical Cancer Research, Bautzner Landstraße 400, Dresden, Germany

Table of Content

Figure S1: ESI(+) mass spectrum after reduction of N¹-Boc-3-oxospermidine 3
Figure S2: ESI(+) mass spectrum after fluorobenzoylation to N¹-Dde-N⁴-FBz-spermidine 3
Figure S3: ¹H NMR spectrum of *N*¹,*N*¹-di(4-fluorobenzoyl)-1,6-diaminohexane×TFA 4
Figure S4: ¹H NMR spectrum of *N*-(4-fluorobenzoyl)-putrescine×TFA (**1**) 5
Figure S5: ¹³C NMR spectrum of *N*-(4-fluorobenzoyl)-putrescine×TFA (**1**) 5
Figure S6: ¹H NMR spectrum of *N*-(4-fluorobenzoyl)-cadaverine×TFA (**2**) 6
Figure S7: ¹³C NMR spectrum of *N*-(4-fluorobenzoyl)-cadaverine×TFA (**2**)................................ 6
Figure S8: ¹H NMR spectrum of *N*¹-(4-fluorobenzoyl)-1,6-diaminohexane×TFA (**3**)................. 7
Figure S9: ¹³C NMR spectrum of *N*¹-(4-fluorobenzoyl)-1,6-diaminohexane×TFA (**3**) 7
Figure S10: ¹H NMR spectrum of *N*¹-(4-fluorobenzoyl)-1,7-diaminoheptane×TFA (**4**) 8
Figure S11: ¹³C NMR spectrum of *N*¹-(4-fluorobenzoyl)-1,7-diaminoheptane×TFA (**4**) 8
Figure S12: ¹H NMR spectrum of *N*¹-(4-fluorobenzoyl)-1,8-diaminooctane×TFA (**5**)................ 8
Figure S13: ¹³C NMR spectrum of *N*¹-(4-fluorobenzoyl)-1,8-diaminooctane×TFA (**5**) 9
Figure S14: ¹H NMR spectrum of *N*¹-(4-fluorobenzoyl)-3-oxospermidine×TFA (**6**)................. 10
Figure S15: ¹³C NMR spectrum of *N*¹-(4-fluorobenzoyl)-3-oxospermidine×TFA (**6**)................ 10
Figure S16: ¹H NMR spectrum of *N*⁸-(4-fluorobenzoyl)-5-oxospermidine×TFA (**7**)................. 11
Figure S17: ¹³C NMR spectrum of *N*⁸-(4-fluorobenzoyl)-5-oxospermidine×TFA (**7**)................ 11
Figure S18: ¹H NMR spectrum of *N*¹-(4-fluorobenzoyl)-3,8-dioxospermidine×TFA (**8**).......... 12
Figure S19: ¹³C NMR spectrum of *N*¹-(4-fluorobenzoyl)-3,8-dioxospermidine×TFA (**8**) 12
Figure S20: ¹H NMR spectrum of *N*¹-(4-fluorobenzoyl)-spermidine×2TFA (**9**) 13
Figure S21: ¹³C NMR spectrum of *N*¹-(4-fluorobenzoyl)-spermidine×2TFA (**9**)........................ 13
Figure S22: ¹H NMR spectrum of *N*⁴-(4-fluorobenzoyl)-spermidine×2TFA (**10**) 14
Figure S23: ¹³C NMR spectrum of *N*⁴-(4-fluorobenzoyl)-spermidine×2TFA (**10**)...................... 14
Figure S24: ¹H NMR spectrum of *N*⁸-(4-fluorobenzoyl)-spermidine×2TFA (**11**) 15
Figure S25: ¹³C NMR spectrum of *N*⁸-(4-fluorobenzoyl)-spermidine×2TFA (**11**)...................... 15
Figure S26: ¹H NMR spectrum of *N*¹-(4-fluorobenzoyl)-spermine×3TFA (**12**).......................... 16
Figure S27: ¹³C NMR spectrum of *N*¹-(4-fluorobenzoyl)-spermine×3TFA (**12**) 16
Figure S28: ¹H NMR spectrum of *N*¹-(4-fluorobenzyl)-putrescine×2TFA (**13**) 17
Figure S29: ¹³C NMR spectrum of *N*¹-(4-fluorobenzyl)-putrescine×2TFA (**13**) 17
Figure S30: ¹H NMR spectrum of *N*¹-(4-fluorobenzyl)-cadaverine×2TFA (**14**) 18
Figure S31: ¹³C NMR spectrum of *N*¹-(4-fluorobenzyl)-cadaverine×2TFA (**14**)......................... 18
Figure S32: ¹H NMR spectrum of *N*¹-(4-fluorobenzyl)-1,6-diaminohexane×2TFA (**15**) 19
Figure S33: ¹³C NMR spectrum of *N*¹-(4-fluorobenzyl)-1,6-diaminohexane×2TFA (**15**) 19
Figure S34: ¹H NMR spectrum of *N*¹-(4-fluorobenzyl)-1,7-diaminoheptane×2TFA (**16**) 20
Figure S35: ¹³C NMR spectrum of *N*¹-(4-fluorobenzyl)-1,7-diaminoheptane×2TFA (**16**)......... 20
Figure S36: ¹H NMR spectrum of *N*¹-(4-fluorobenzyl)-1,8-diaminooctane×2TFA (**17**)............ 21
Figure S37: ¹³C NMR spectrum of *N*¹-(4-fluorobenzyl)-1,8-diaminooctane×2TFA (**17**) 21
Figure S38: ¹H NMR spectrum of *N*¹-(4-fluorobenzyl)-spermine×4TFA (**18**)........................... 22
Figure S39: ¹³C NMR spectrum of *N*¹-(4-fluorobenzyl)-spermine×4TFA (**18**) 22

Figure S1: ESI(+) mass spectrum after reduction of *N*[1]-Boc-3-oxospermidine

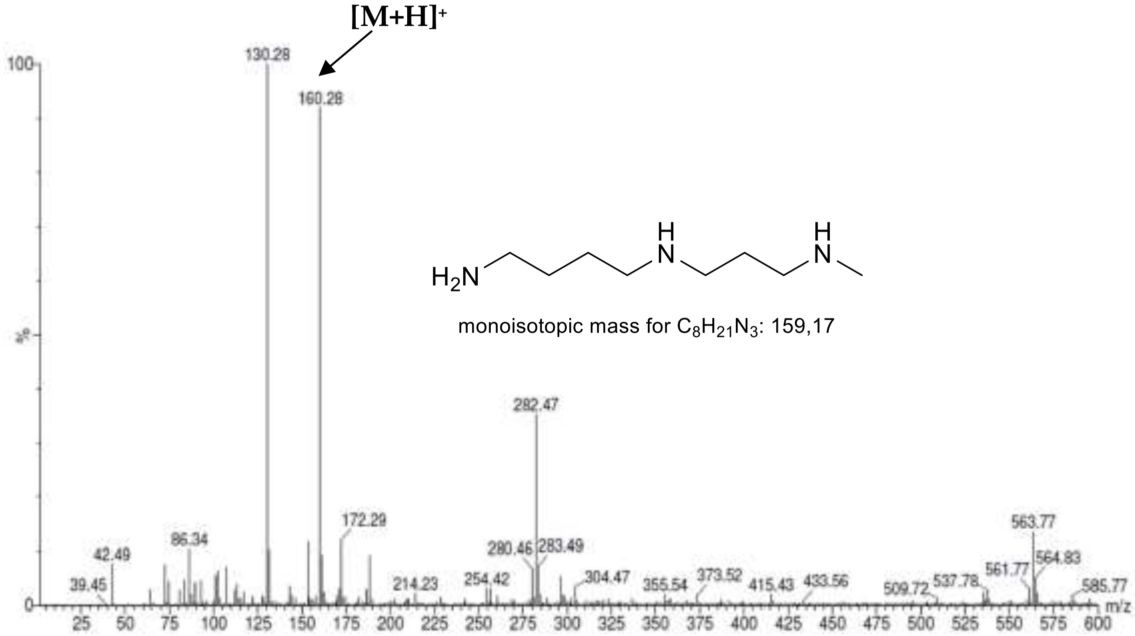

Figure S2: ESI(+) mass spectrum after fluorobenzoylation to *N*[1]-Dde-*N*[4]-FBz-spermidine

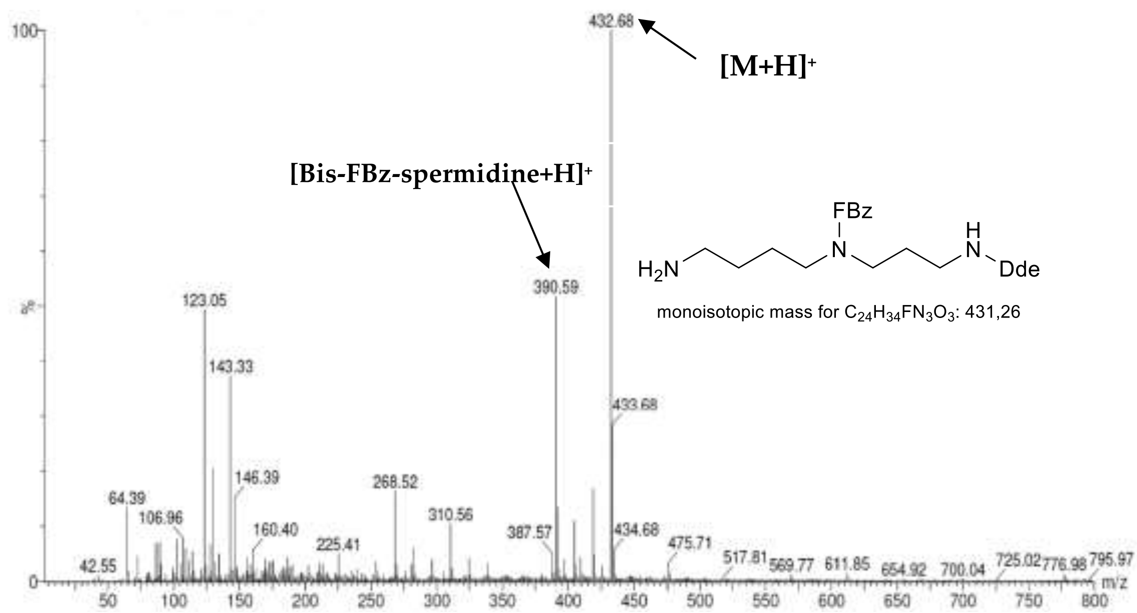

Figure S3: ^{1}H NMR spectrum of *N*^{1},*N*^{1}-di(4-fluorobenzoyl)-1,6-diaminohexane×TFA (side product formed during reductive fluorobenzylation)

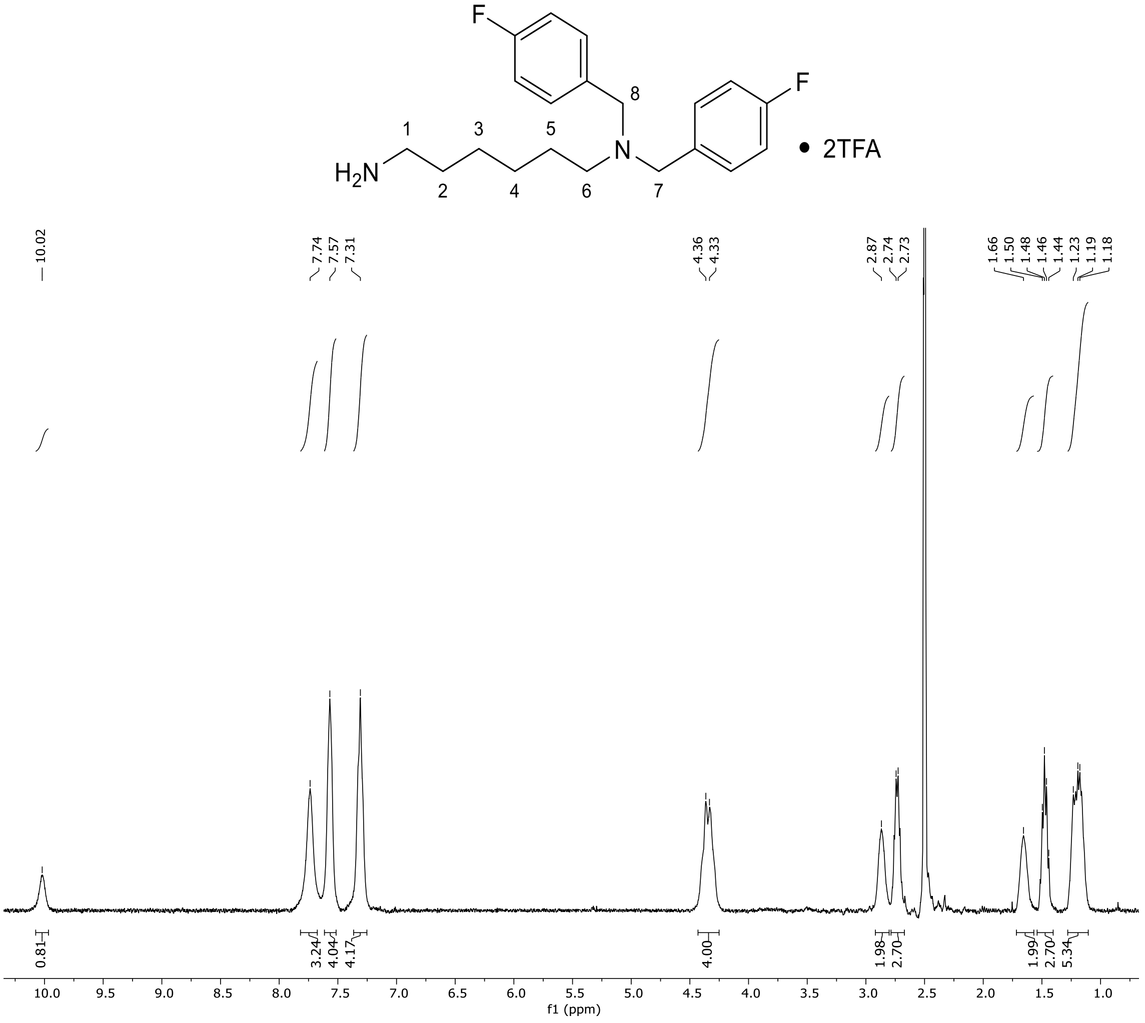

yield: 19 mg colorless oil

MS (ESI+): m/z=332.97 ([M+H]$^{+}$)

M (monoisotopic) calculated for $C_{20}H_{26}F_2N_2$: 332.20

^{1}H NMR (400 MHz, DMSO-*d*$_6$) δ=10.02 (s, 1H, NH^{+}), 7.74 (s, 3H, NH_3^{+}), 7.57 (s, 4H, H-2,6 FBn), 7.31 (s, 4H, H-3,5 FBn), 4.35 (d, ^{3}J = 12.3 Hz, 4H, H-7/8), 2.87 (s, 2H), 2.73 (d, ^{3}J = 6.9 Hz, 2H), 1.66 (s, 2H), 1.53 – 1.42 (m, 2H), 1.28 – 1.10 (m, 4H).

Figure S4: ^{1}H NMR spectrum of *N*-(4-fluorobenzoyl)-putrescine×TFA (**1**)

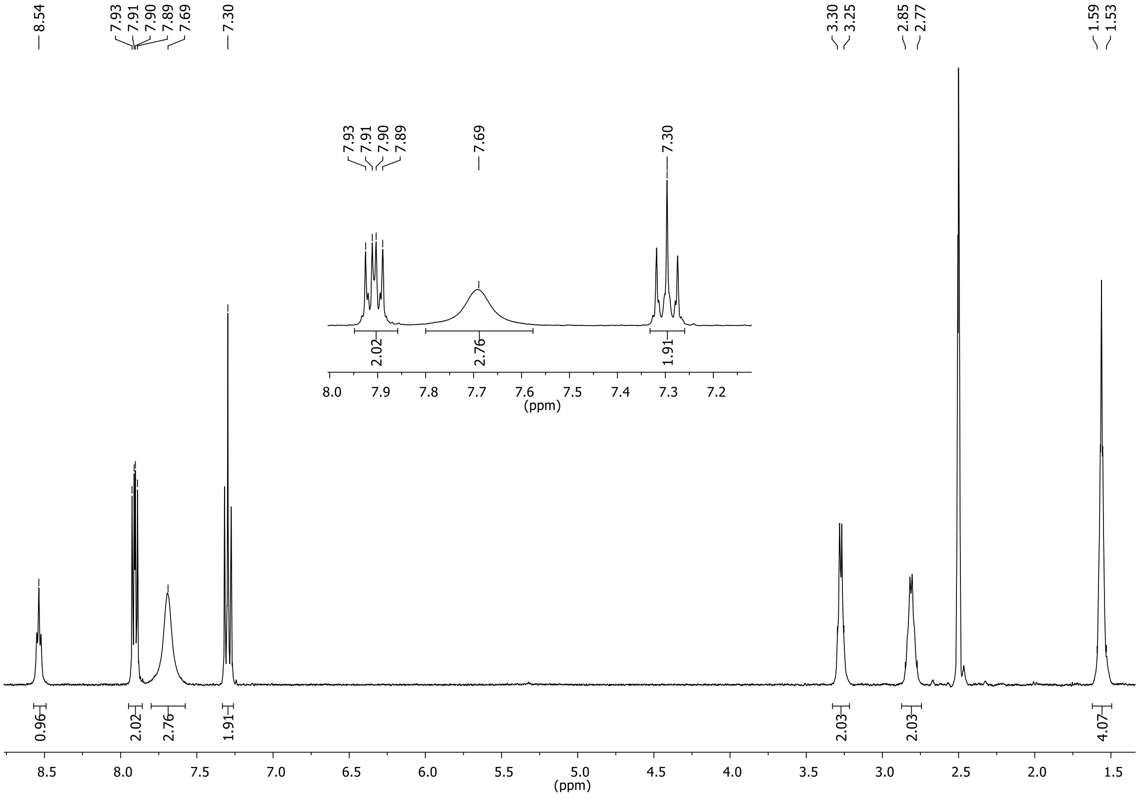

Figure S5: ^{13}C NMR spectrum of *N*-(4-fluorobenzoyl)-putrescine×TFA (**1**)

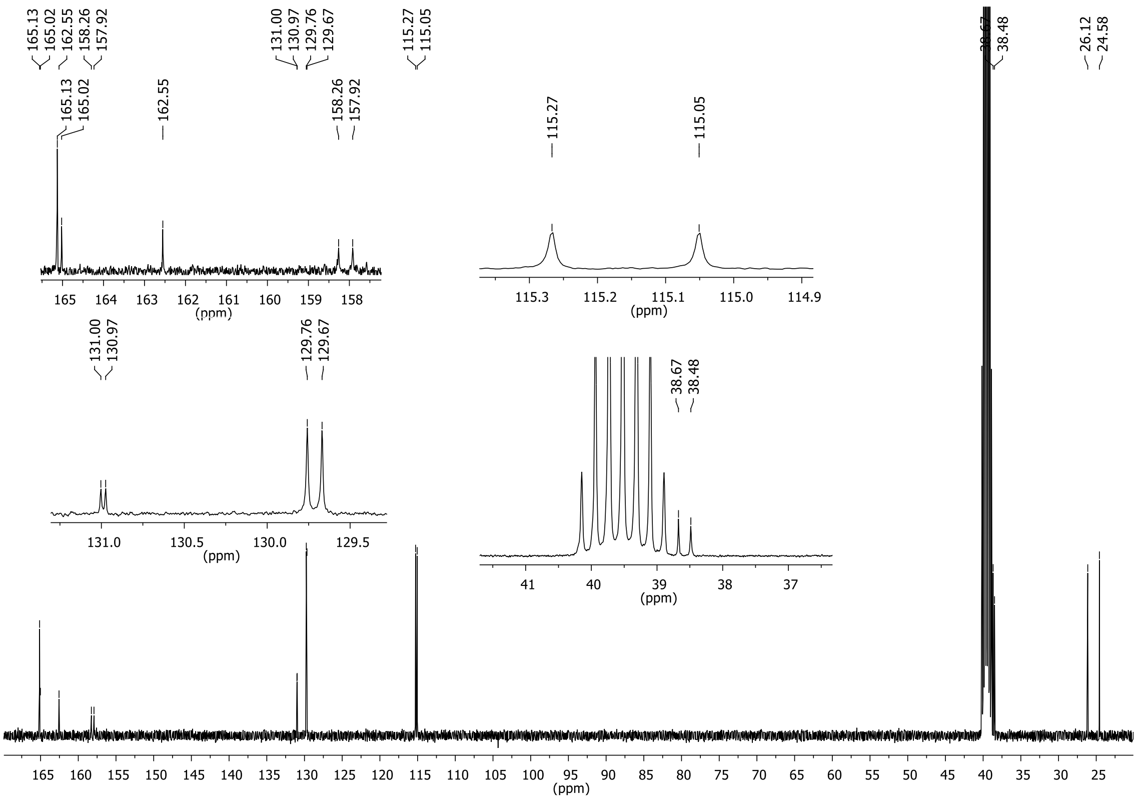

Figure S6: ^{1}H NMR spectrum of *N*-(4-fluorobenzoyl)-cadaverine×TFA (**2**)

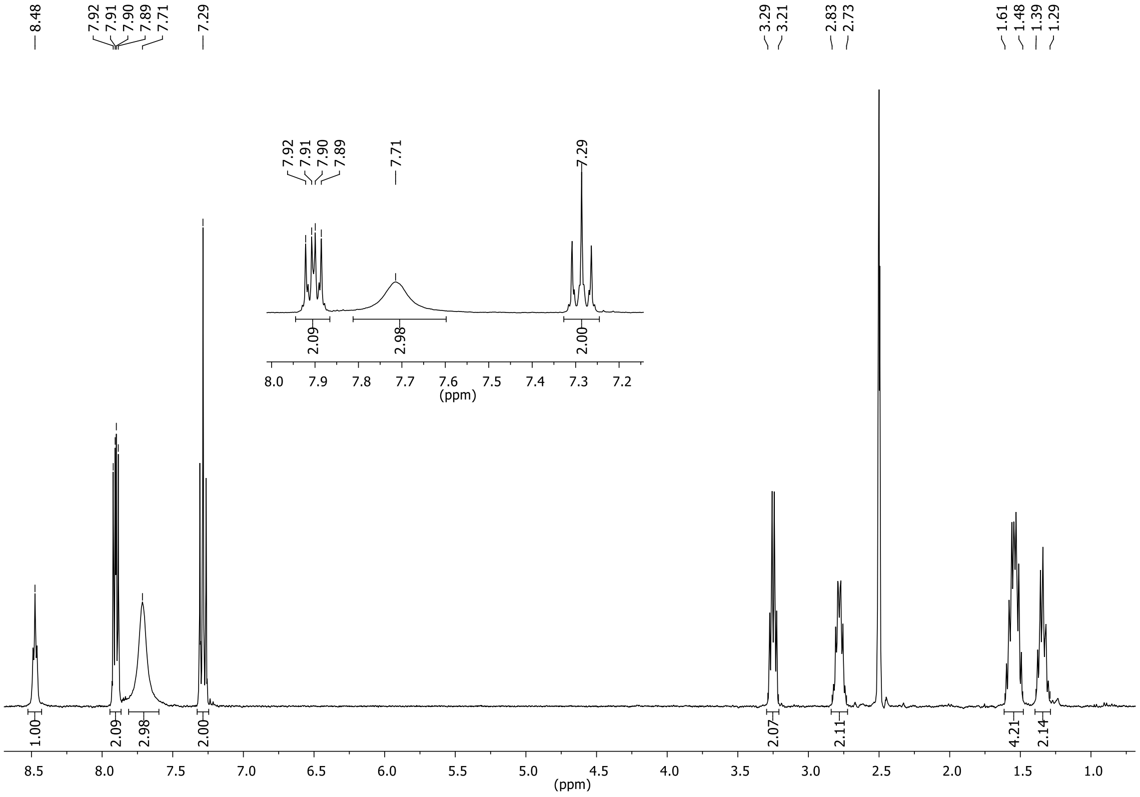

Figure S7: ^{13}C NMR spectrum of *N*-(4-fluorobenzoyl)-cadaverine×TFA (**2**)

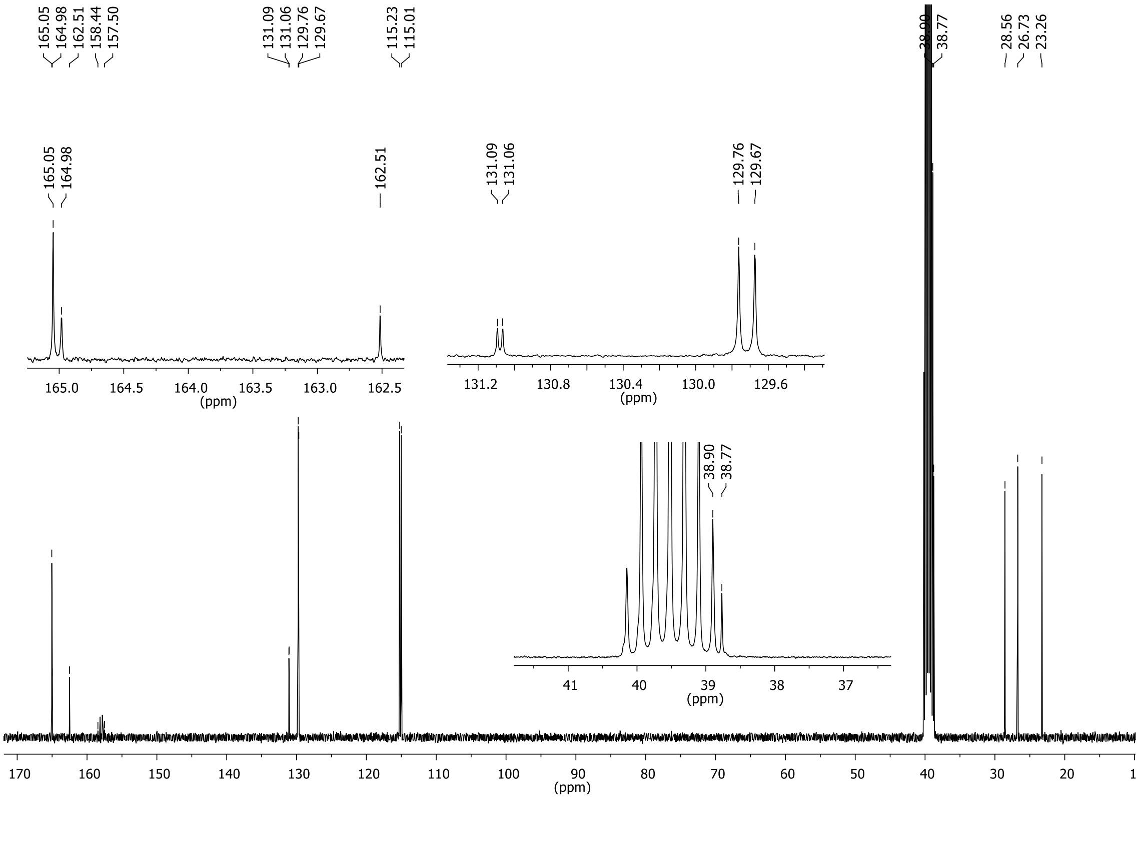

Figure S8: ^{1}H NMR spectrum of *N*^{1}-(4-fluorobenzoyl)-1,6-diaminohexane×TFA (**3**)

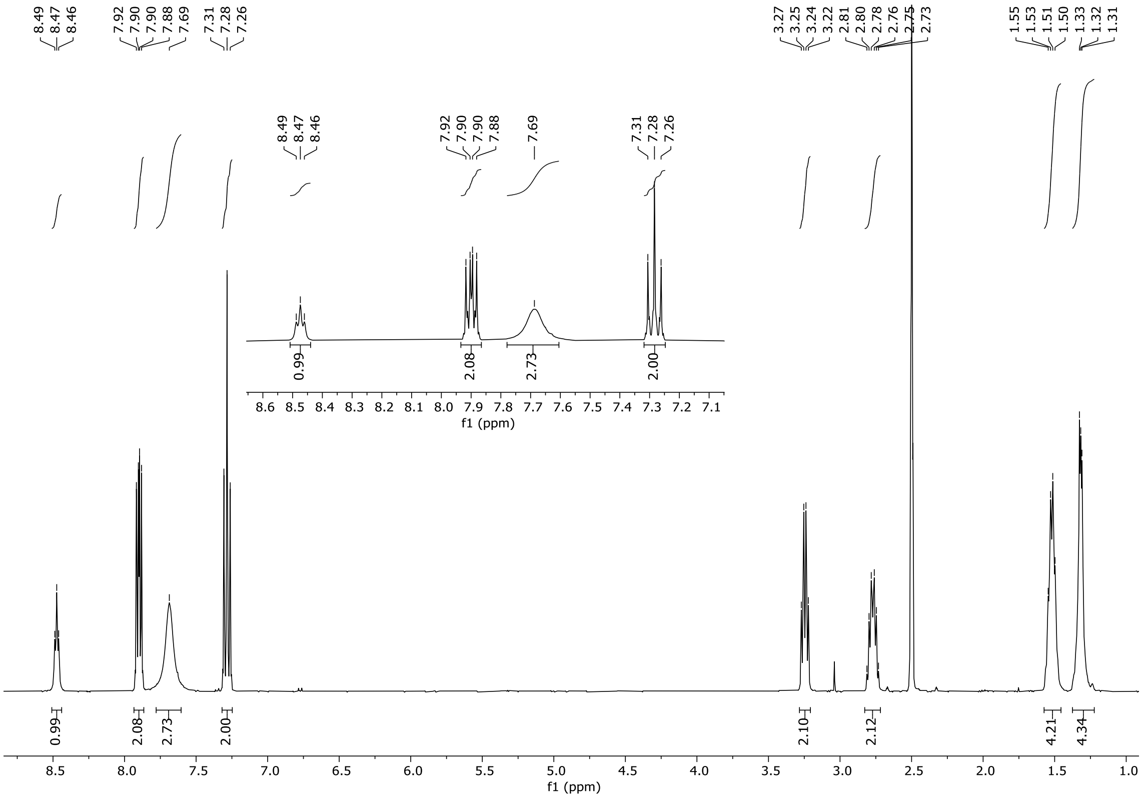

Figure S9: ^{13}C NMR spectrum of *N*^{1}-(4-fluorobenzoyl)-1,6-diaminohexane×TFA (**3**)

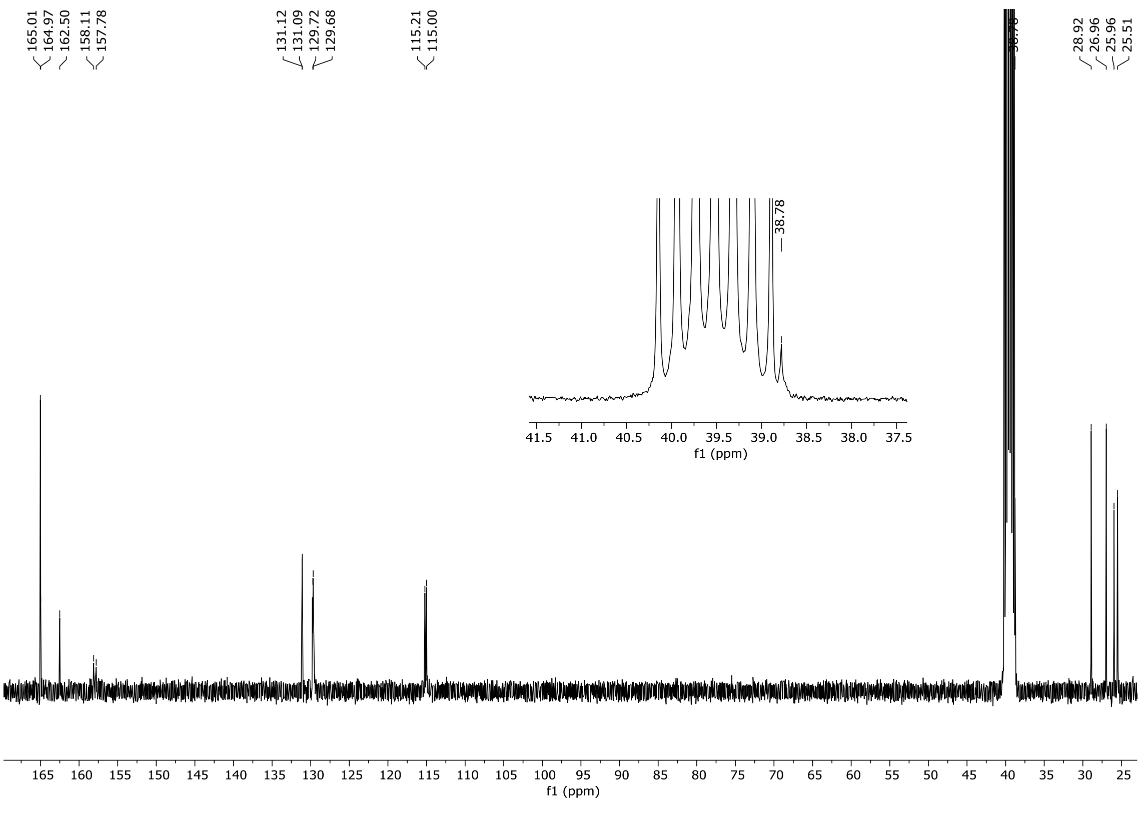

Figure S10: ^{1}H NMR spectrum of *N*^{1}-(4-fluorobenzoyl)-1,7-diaminoheptane×TFA (**4**)

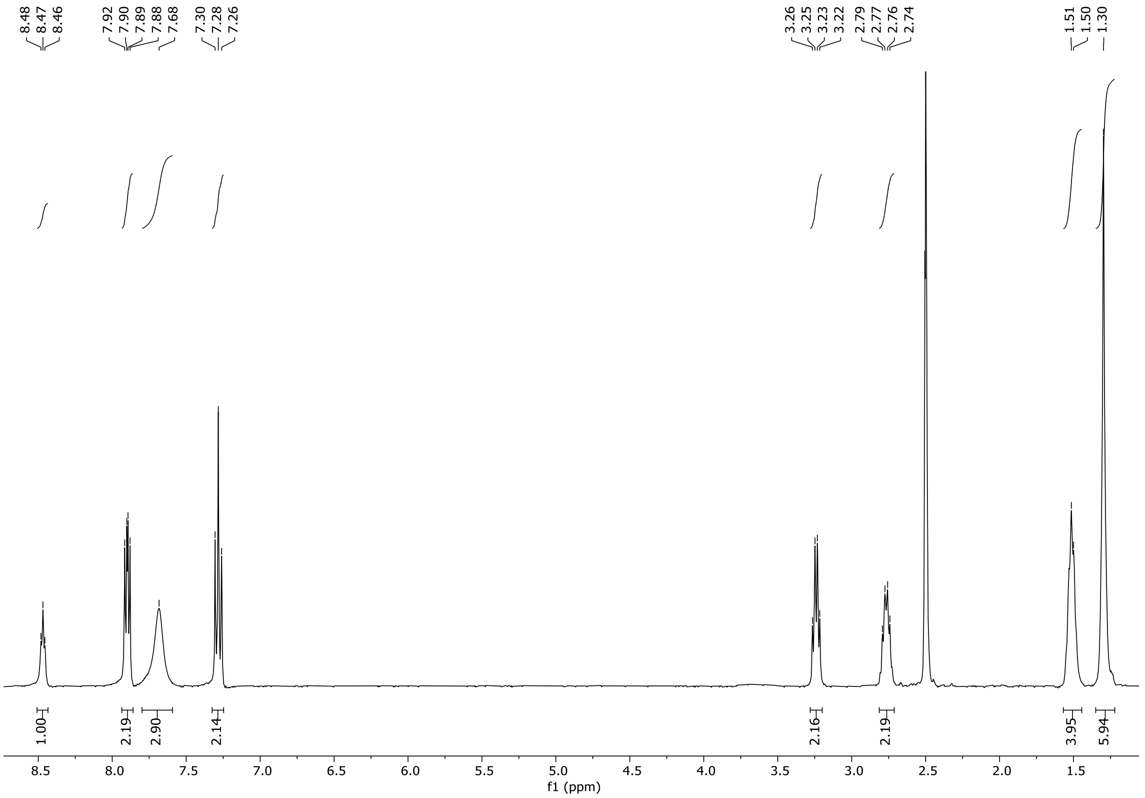

Figure S11: ^{13}C NMR spectrum of *N*^{1}-(4-fluorobenzoyl)-1,7-diaminoheptane×TFA (**4**)

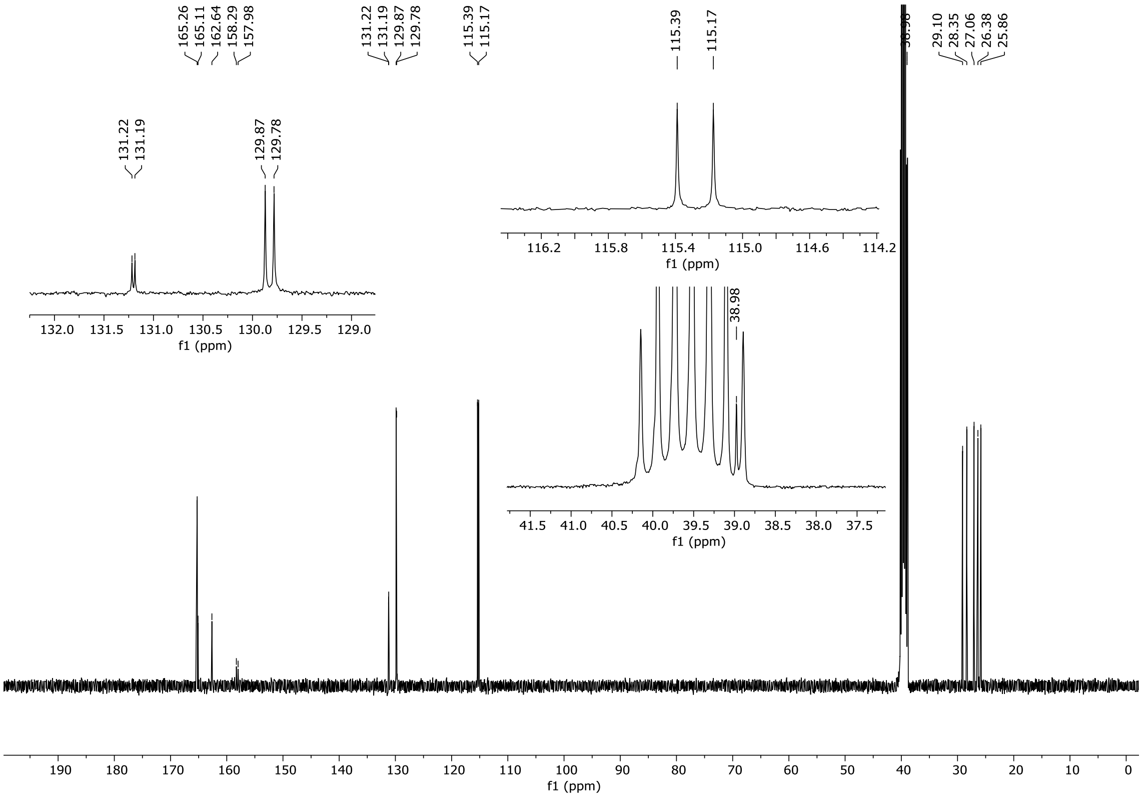

Figure S12: ^{1}H NMR spectrum of *N*^{1}-(4-fluorobenzoyl)-1,8-diaminooctane×TFA (**5**)

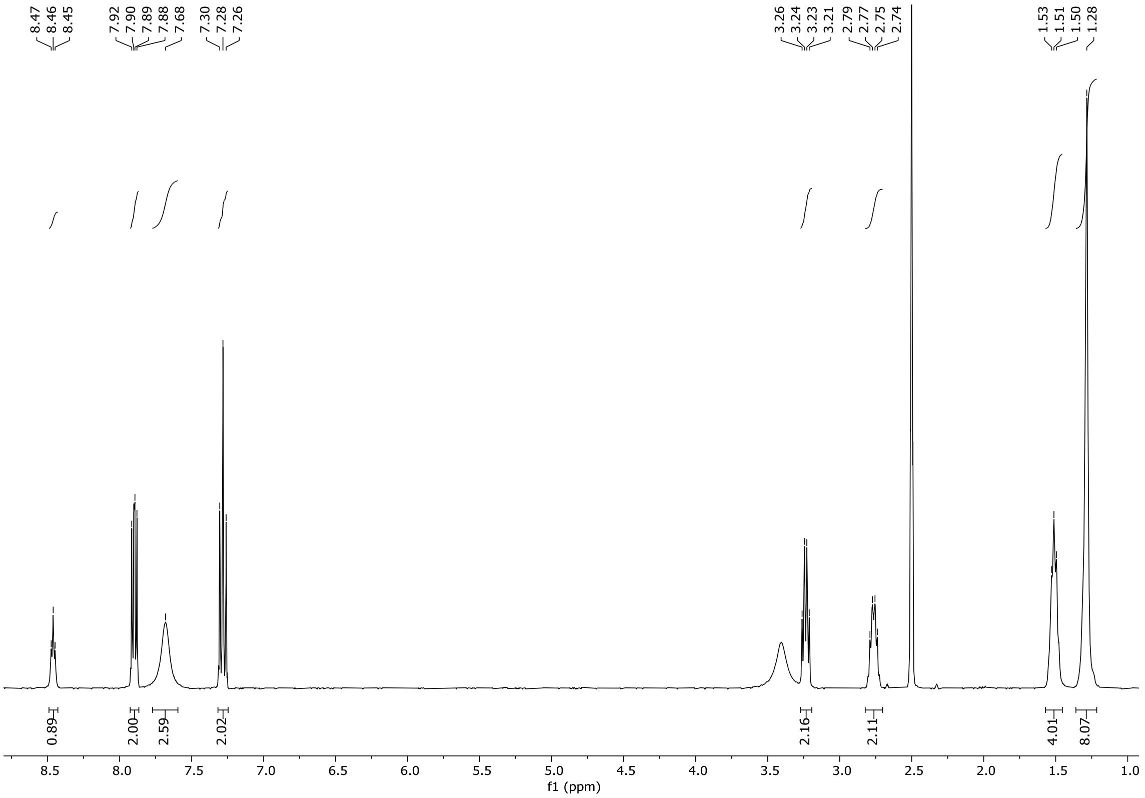

Figure S13: ^{13}C NMR spectrum of *N*[1]-(4-fluorobenzoyl)-1,8-diaminooctane×TFA (**5**)

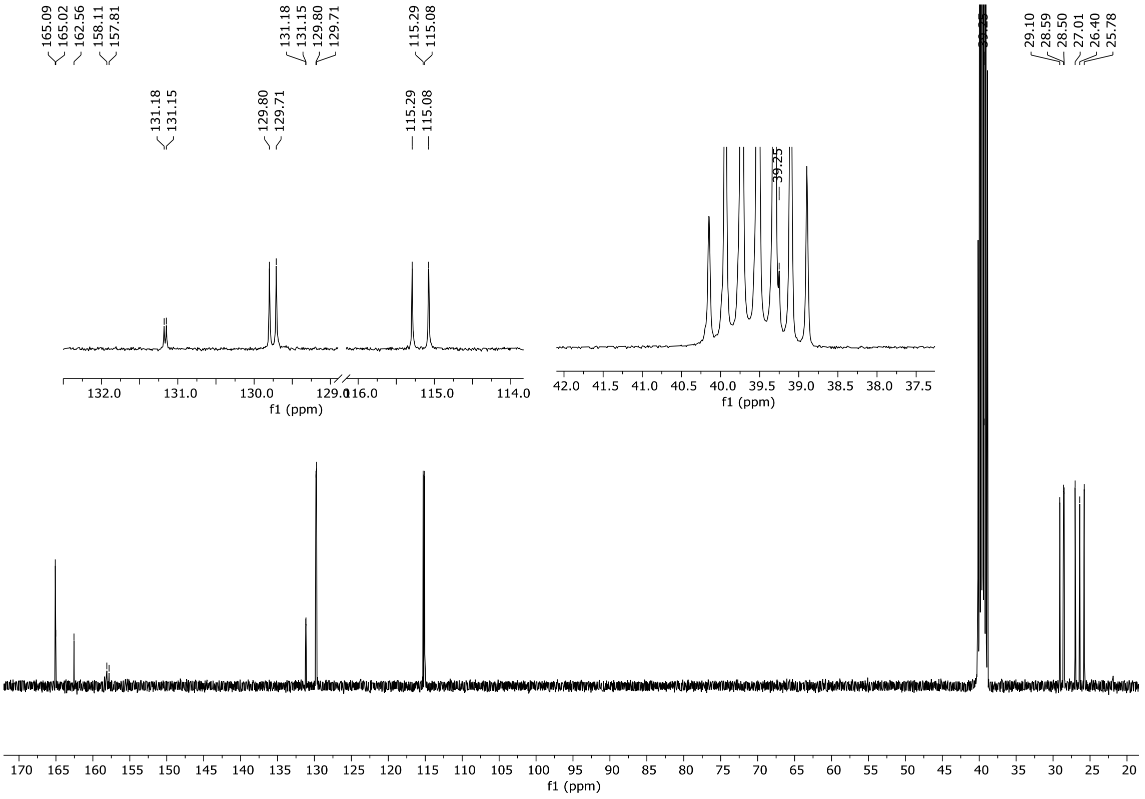

Figure S14: ^{1}H NMR spectrum of *N*[1]-(4-fluorobenzoyl)-3-oxospermidine×TFA (**6**)

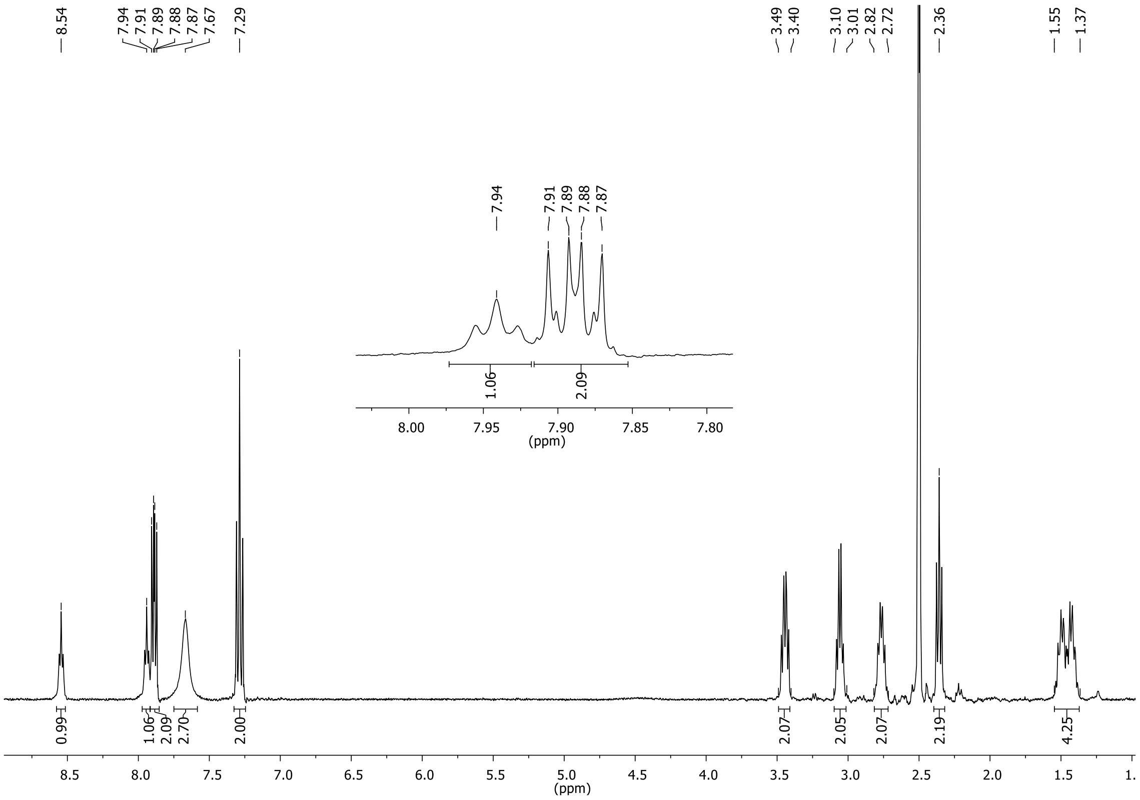

Figure S15: ^{13}C NMR spectrum of *N*[1]-(4-fluorobenzoyl)-3-oxospermidine×TFA (**6**)

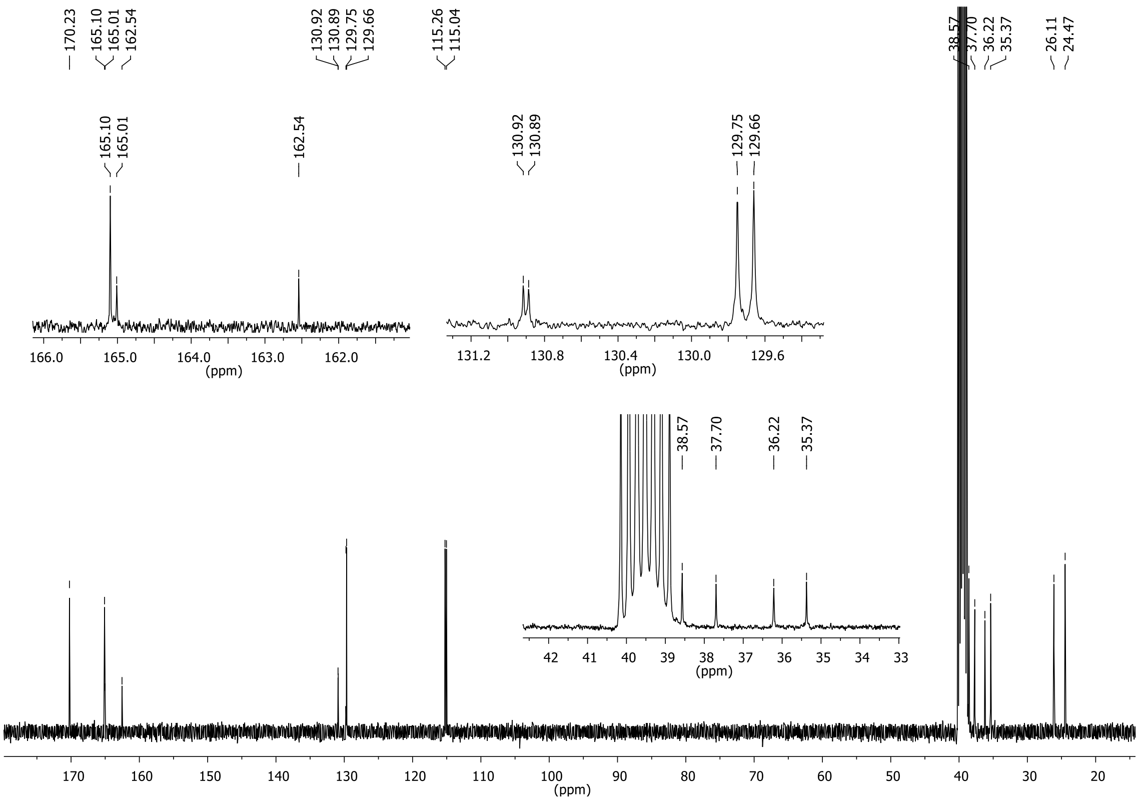

Figure S16: ^{1}H NMR spectrum of *N*^{8}-(4-fluorobenzoyl)-5-oxospermidine×TFA (**7**)

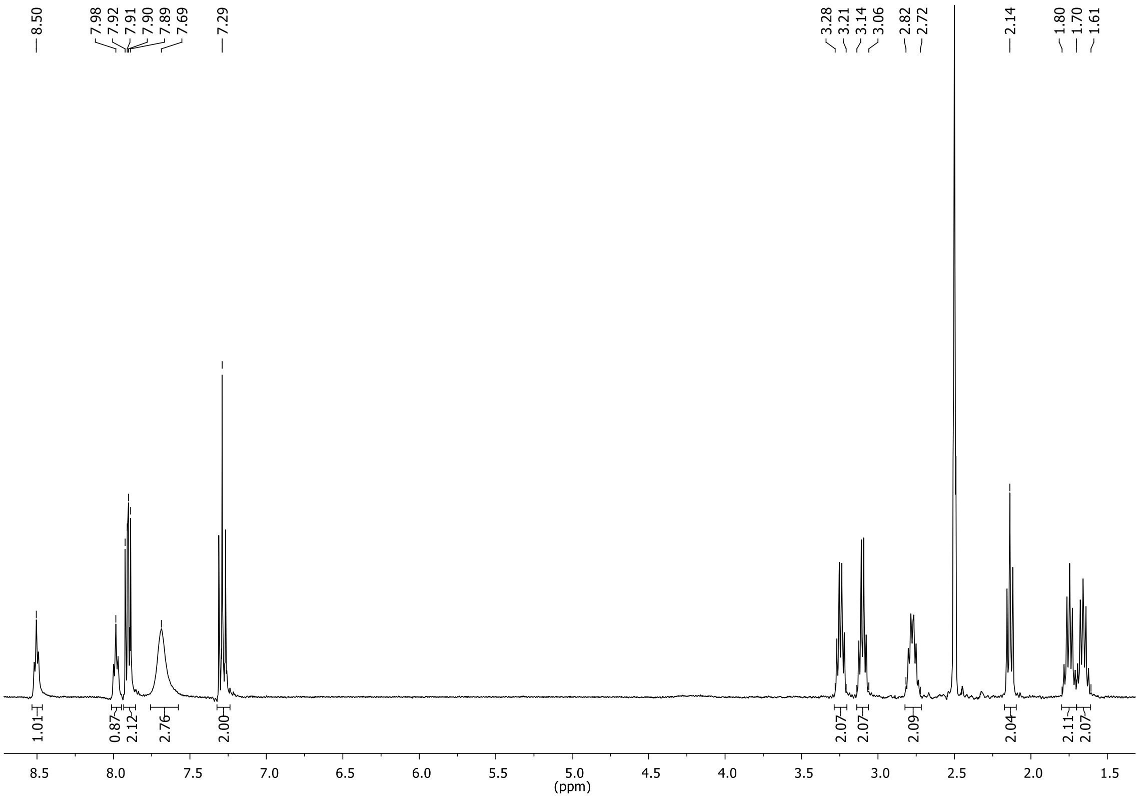

Figure S17: ^{13}C NMR spectrum of *N*^{8}-(4-fluorobenzoyl)-5-oxospermidine×TFA (**7**)

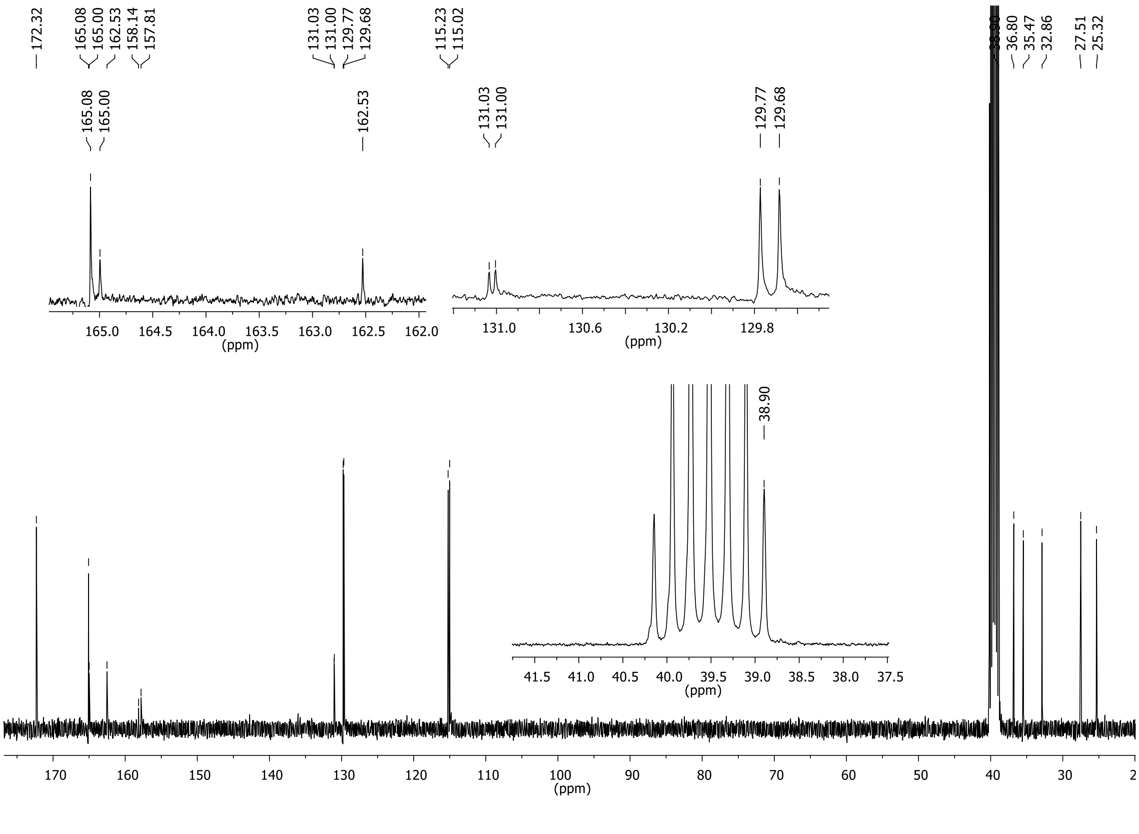

Figure S18: ^{1}H NMR spectrum of *N*^{1}-(4-fluorobenzoyl)-3,8-dioxospermidine×TFA (**8**)

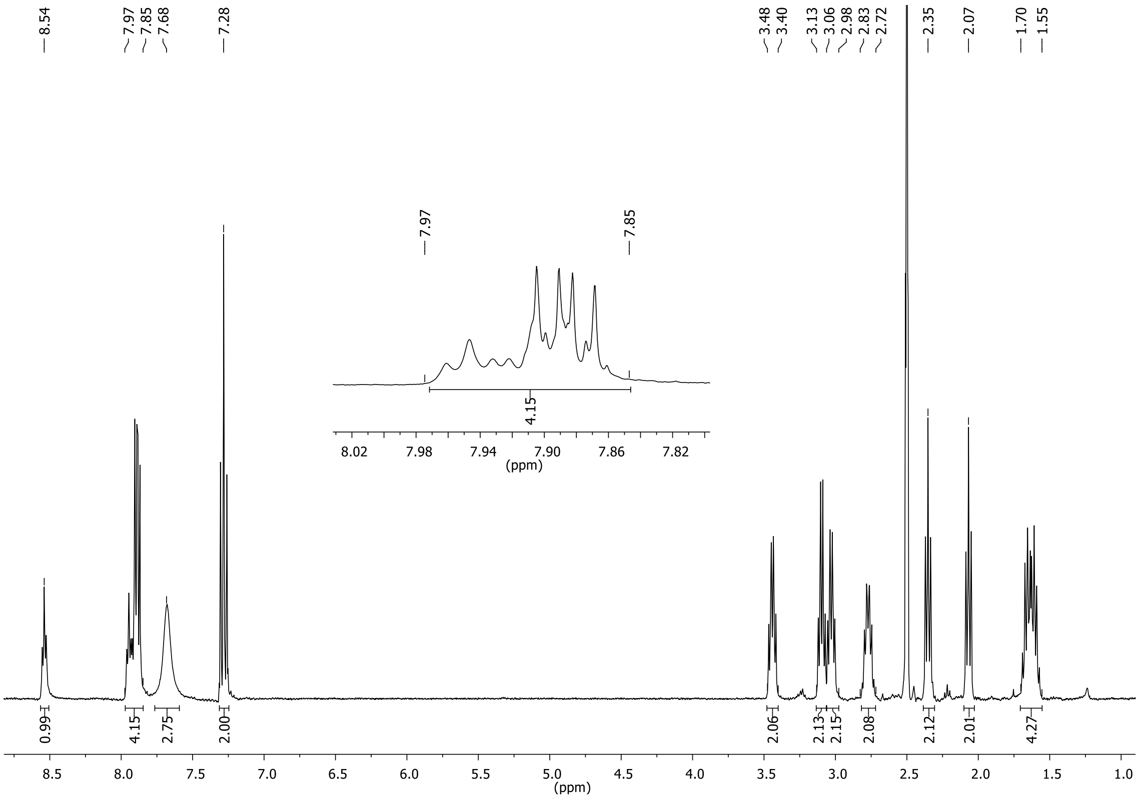

Figure S19: ^{13}C NMR spectrum of *N*^{1}-(4-fluorobenzoyl)-3,8-dioxospermidine×TFA (**8**)

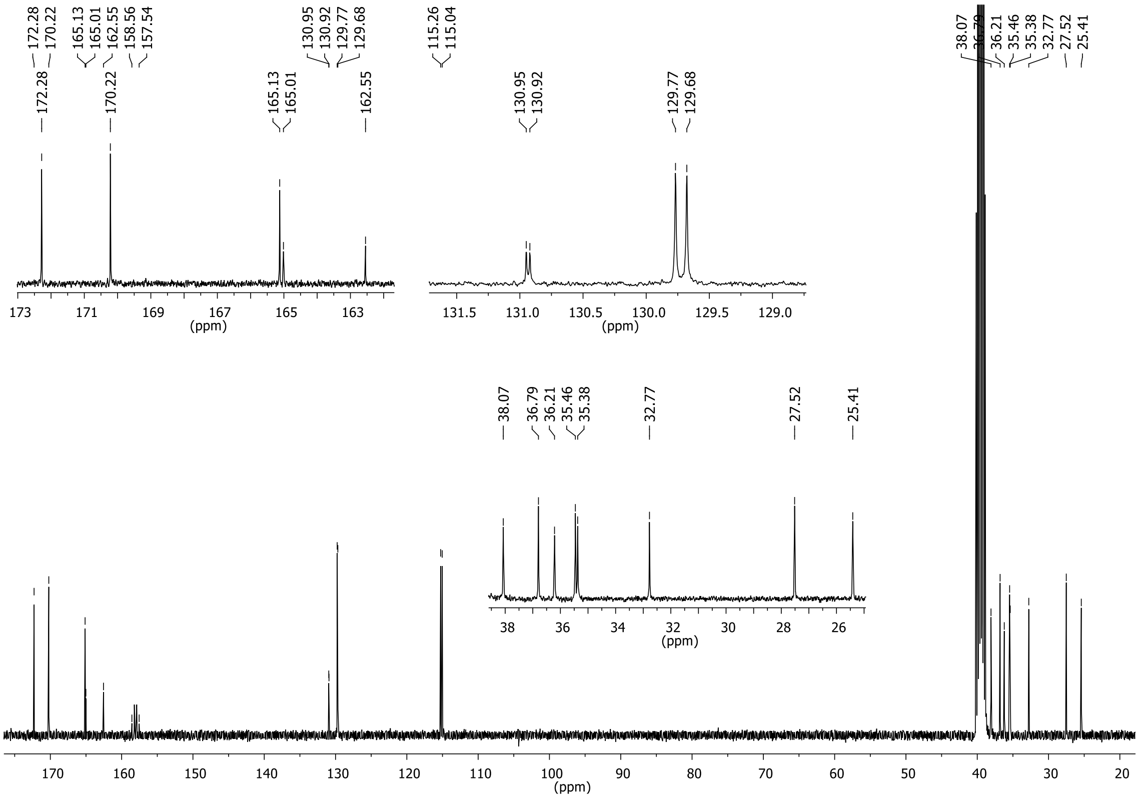

Figure S20: ^{1}H NMR spectrum of *N*[1]-(4-fluorobenzoyl)-spermidine×2TFA (**9**)

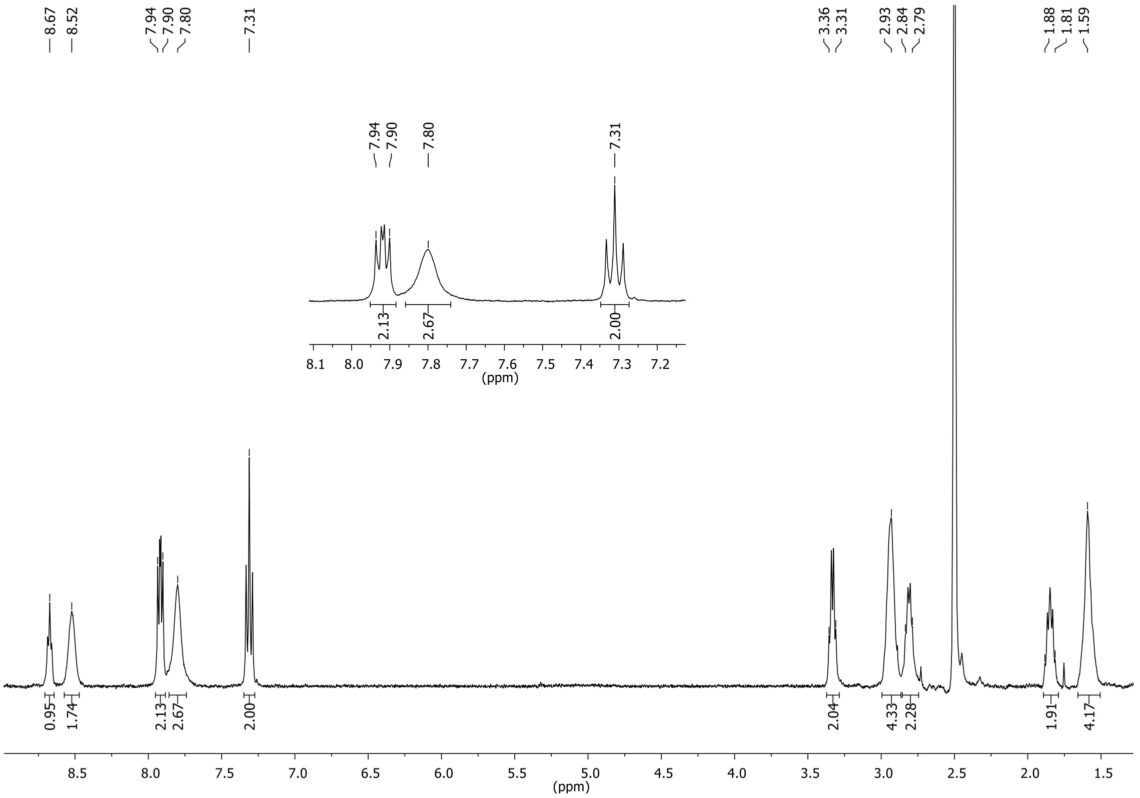

Figure S21: ^{13}C NMR spectrum of *N*[1]-(4-fluorobenzoyl)-spermidine×2TFA (**9**)

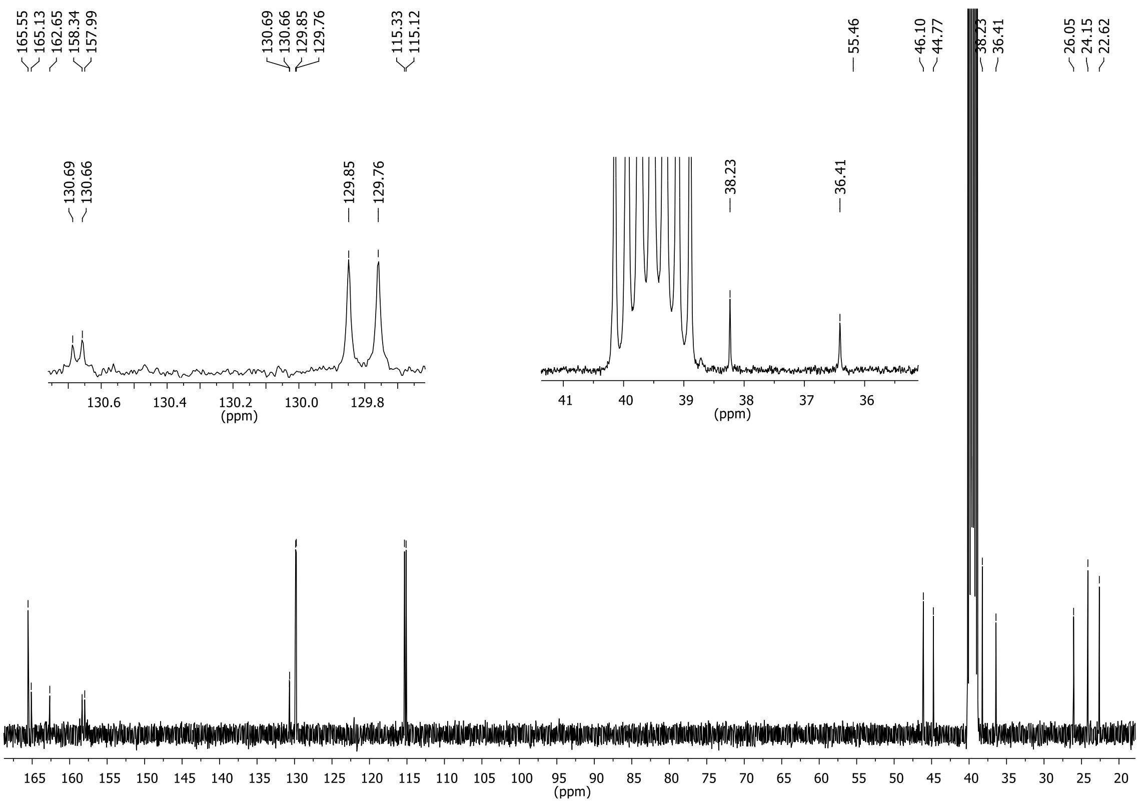

Figure S22: ^{1}H NMR spectrum of *N*^{4}-(4-fluorobenzoyl)-spermidine×2TFA (**10**)

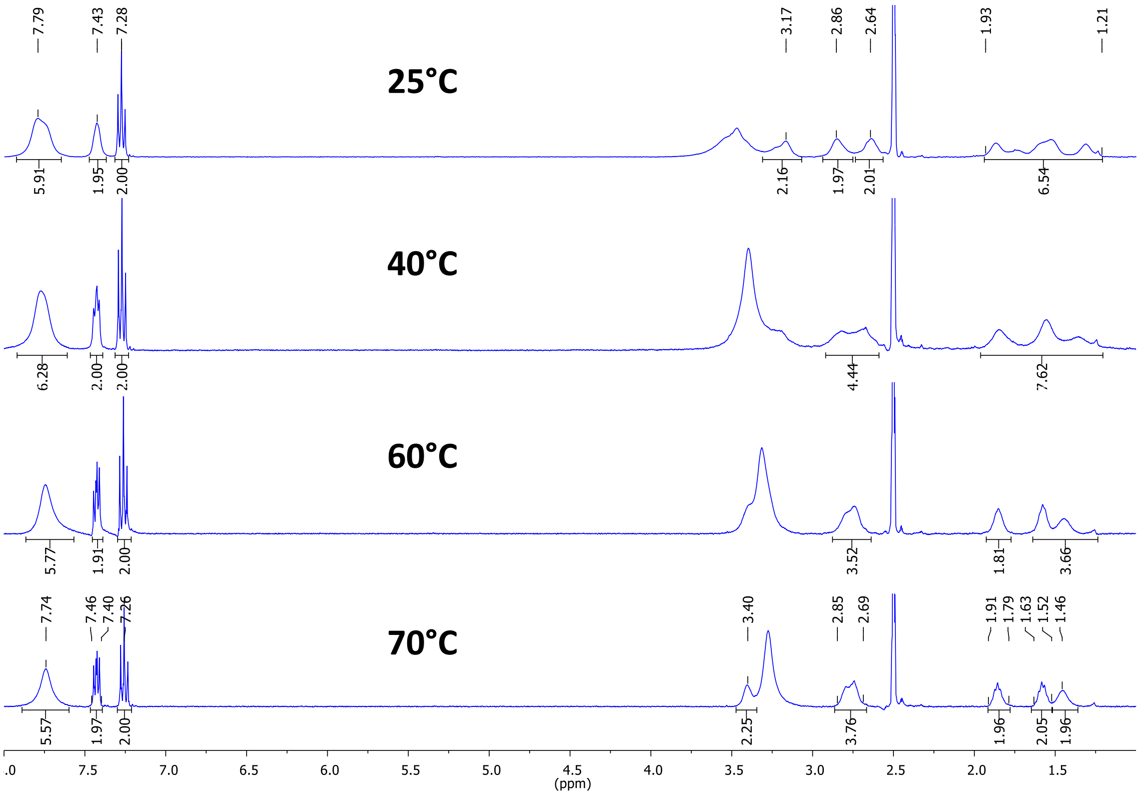

Figure S23: ^{13}C NMR spectrum of *N*^{4}-(4-fluorobenzoyl)-spermidine×2TFA (**10**)

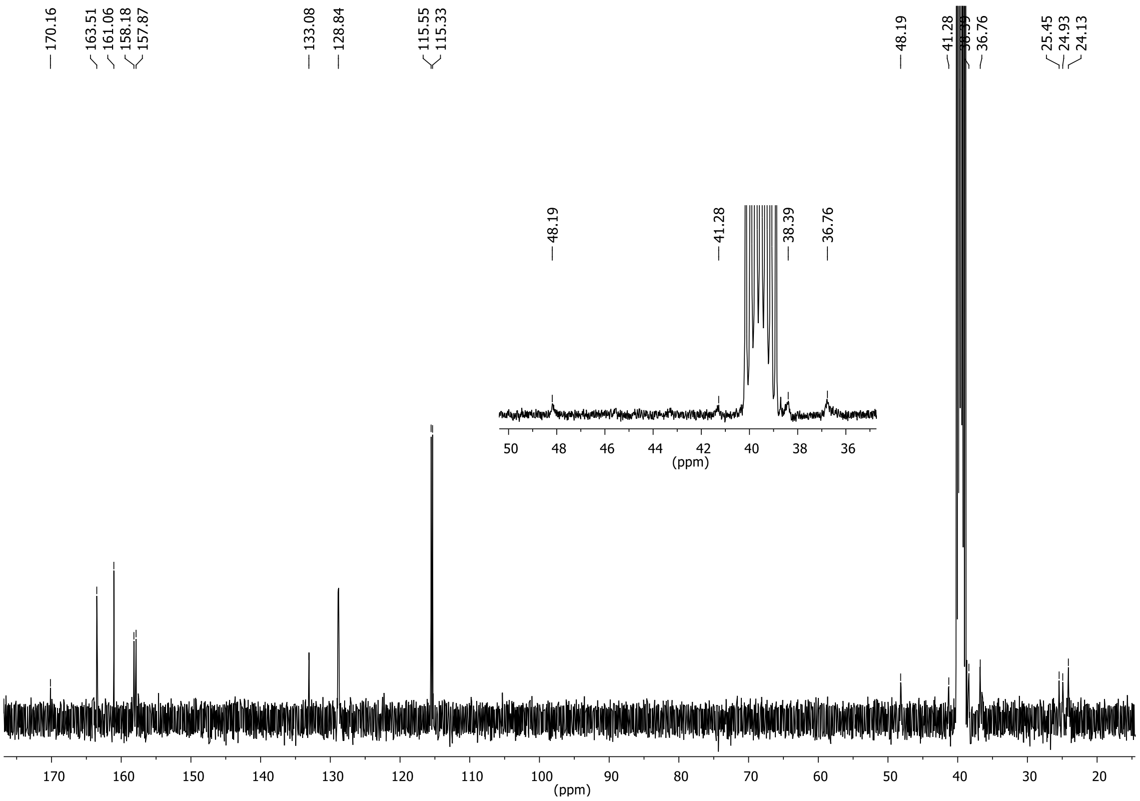

Figure S24: ^{1}H NMR spectrum of N^8-(4-fluorobenzoyl)-spermidine×2TFA (**11**)

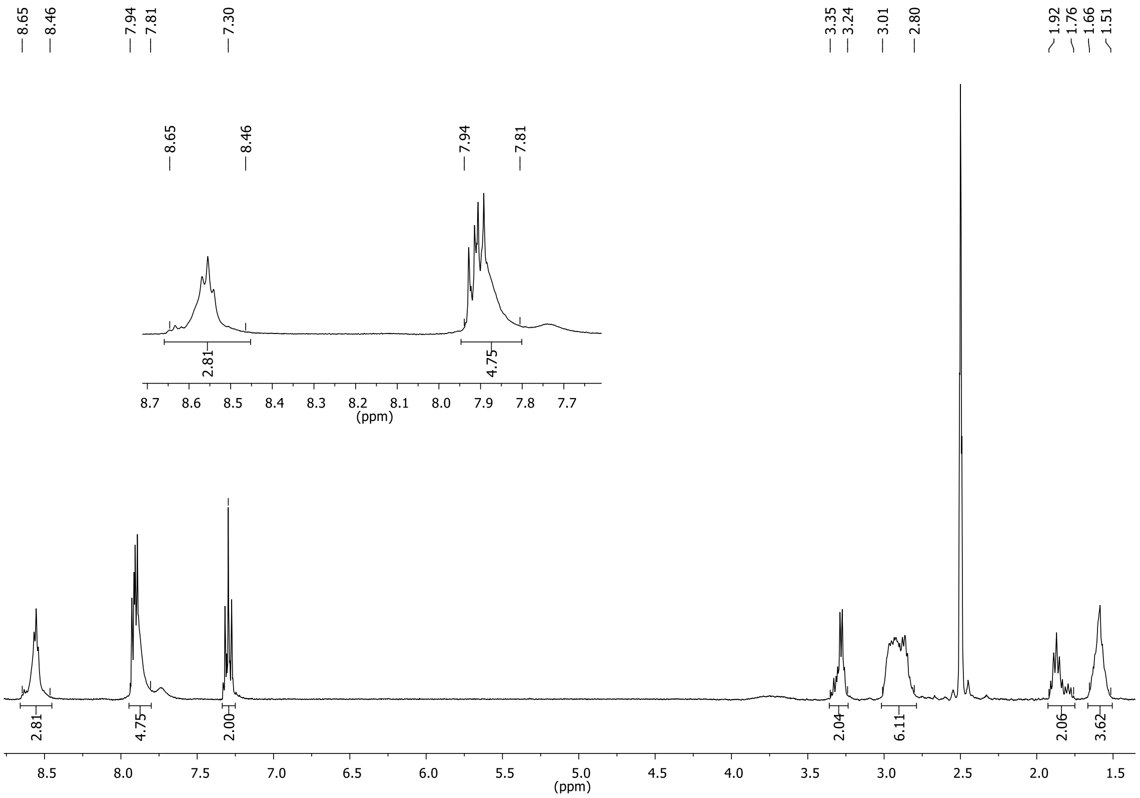

Figure S25: ^{13}C NMR spectrum of N^8-(4-fluorobenzoyl)-spermidine×2TFA (**11**)

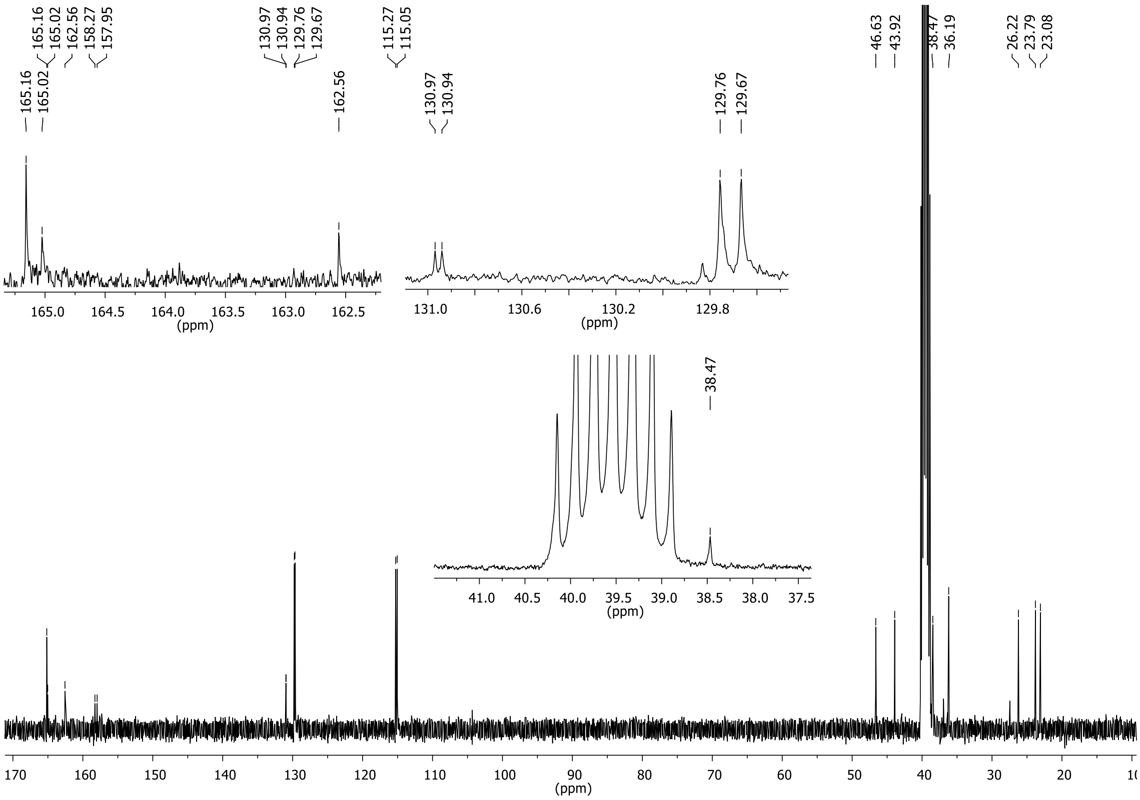

Figure S26: ^{1}H NMR spectrum of *N*[1]-(4-fluorobenzoyl)-spermine×3TFA (**12**)

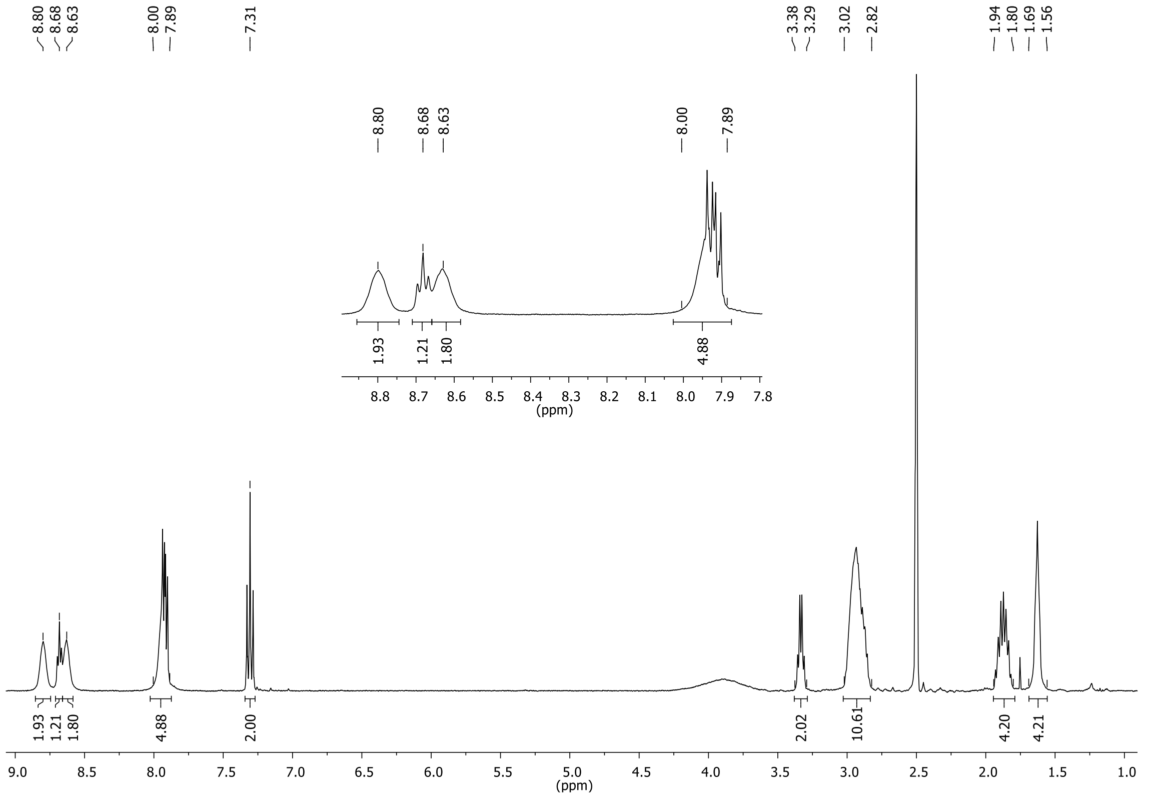

Figure S27: ^{13}C NMR spectrum of *N*[1]-(4-fluorobenzoyl)-spermine×3TFA (**12**)

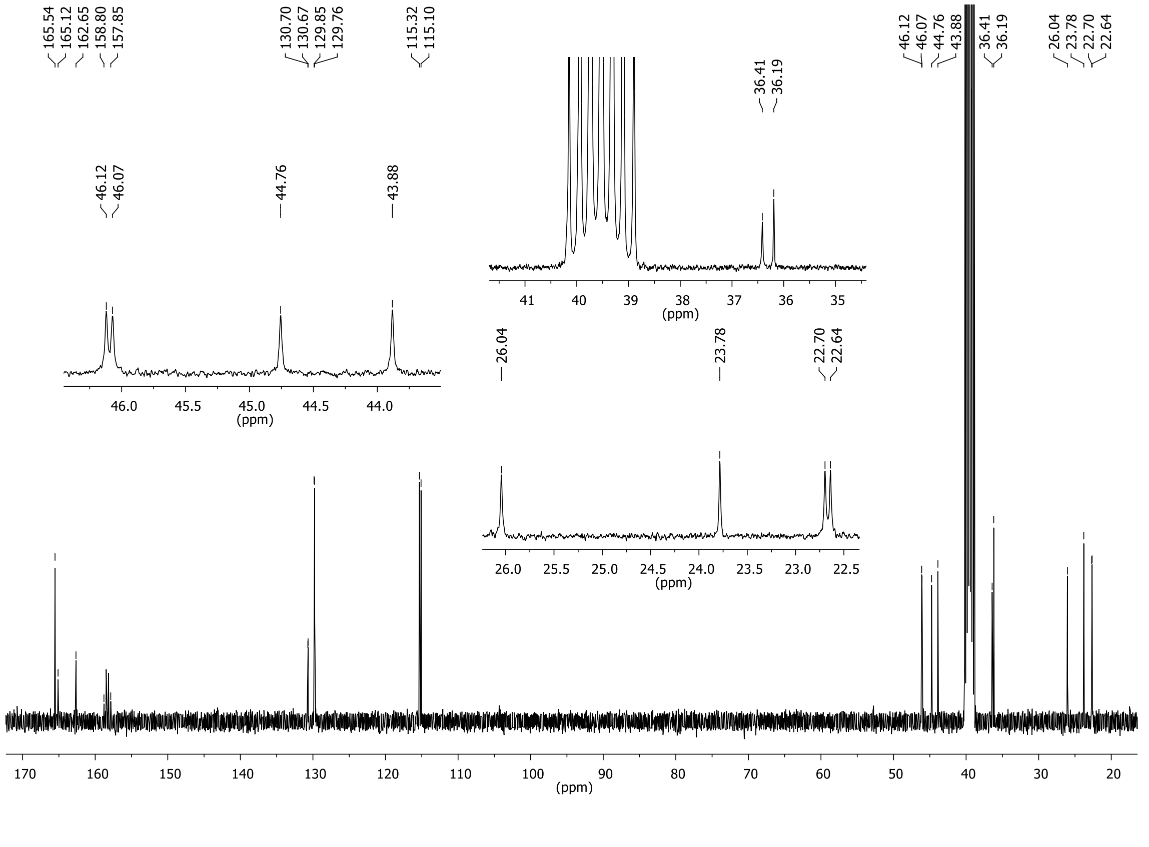

Figure S28: ^{1}H NMR spectrum of *N*[1]-(4-fluorobenzyl)-putrescine×2TFA (**13**)

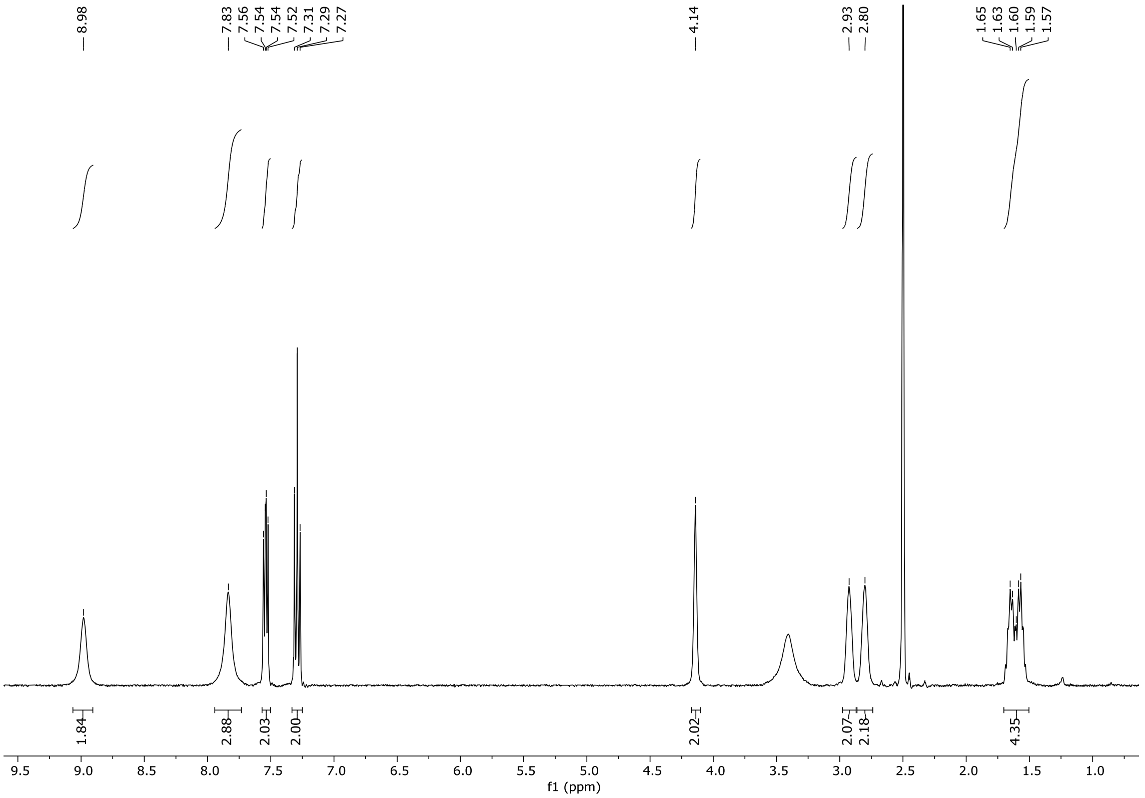

Figure S29: ^{13}C NMR spectrum of *N*[1]-(4-fluorobenzyl)-putrescine×2TFA (**13**)

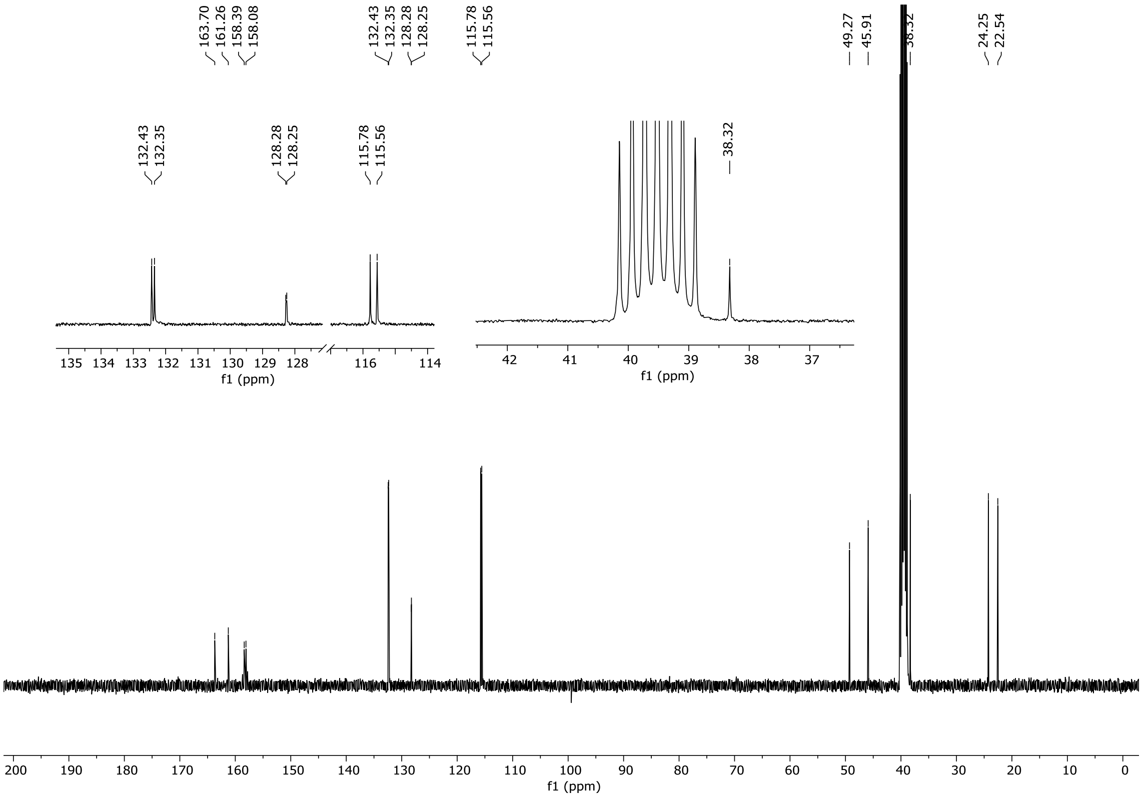

Figure S30: ^{1}H NMR spectrum of *N*^{1}-(4-fluorobenzyl)-cadaverine×2TFA (**14**)

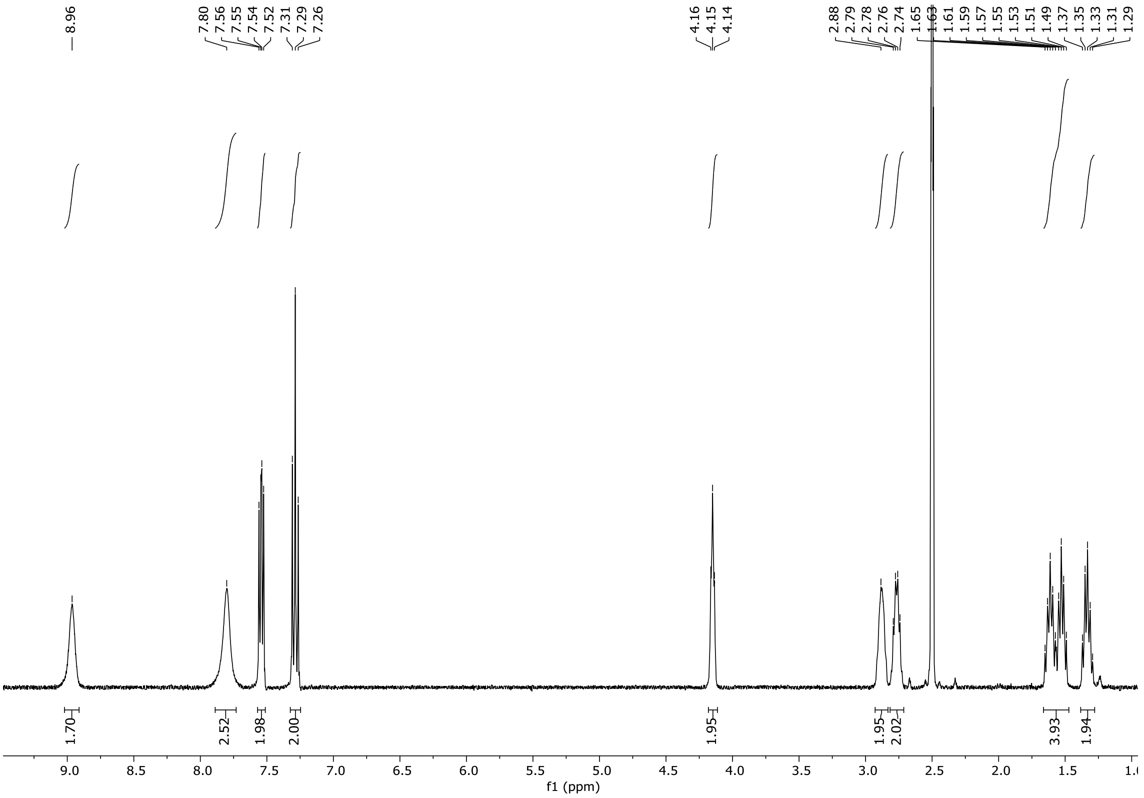

Figure S31: ^{13}C NMR spectrum of *N*^{1}-(4-fluorobenzyl)-cadaverine×2TFA (**14**)

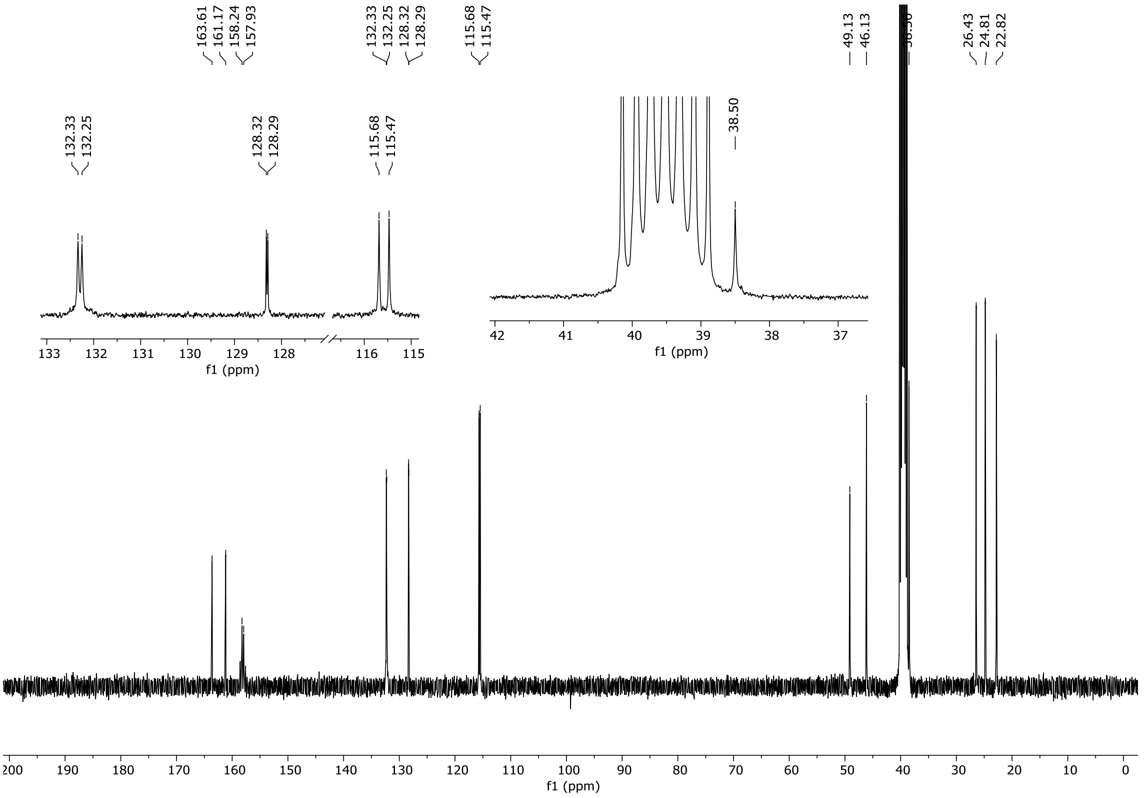

Figure S32: ^{1}H NMR spectrum of *N*[1]-(4-fluorobenzyl)-1,6-diaminohexane×2TFA (**15**)

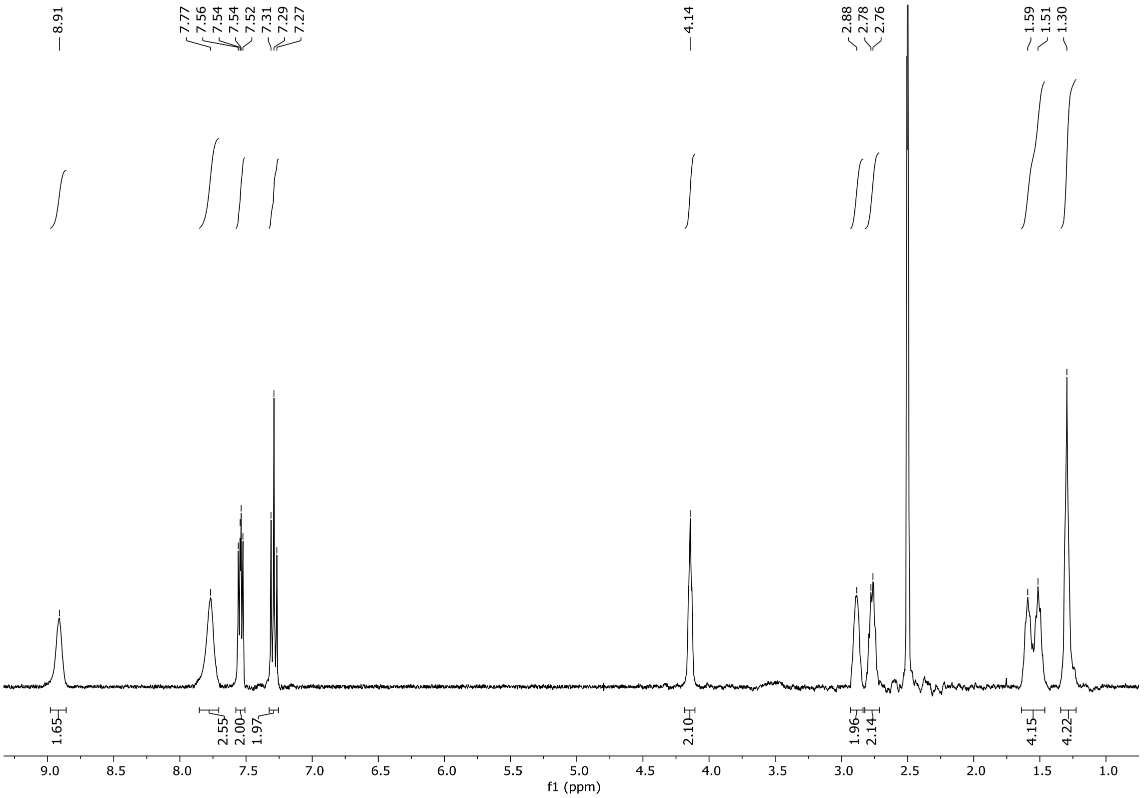

Figure S33: ^{13}C NMR spectrum of *N*[1]-(4-fluorobenzyl)-1,6-diaminohexane×2TFA (**15**)

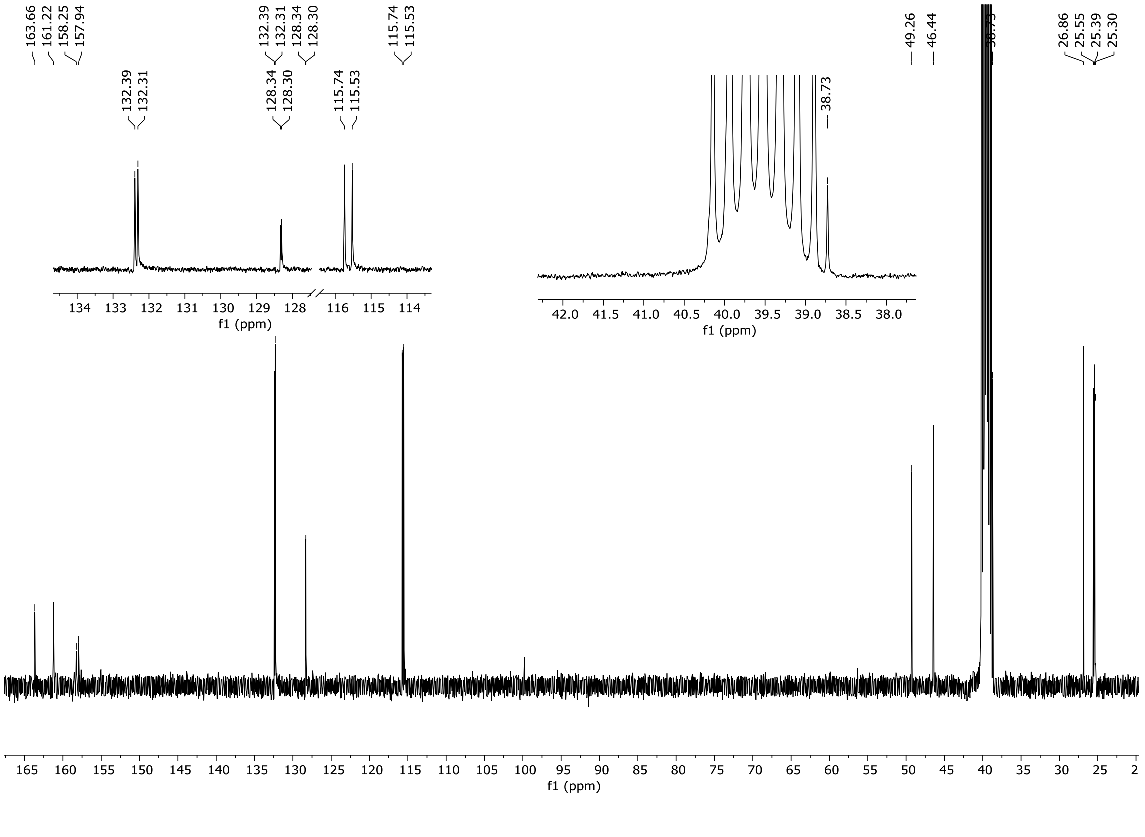

Figure S34: ^{1}H NMR spectrum of *N*^{1}-(4-fluorobenzyl)-1,7-diaminoheptane×2TFA (**16**)

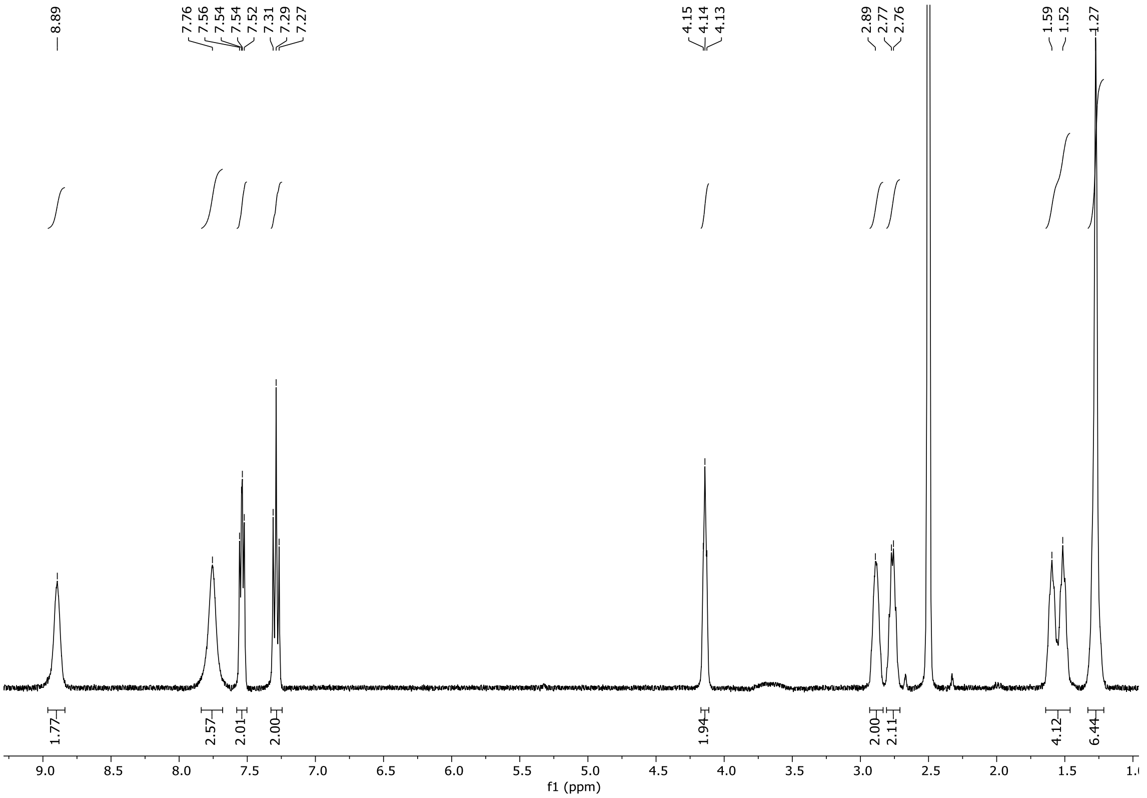

Figure S35: ^{13}C NMR spectrum of *N*^{1}-(4-fluorobenzyl)-1,7-diaminoheptane×2TFA (**16**)

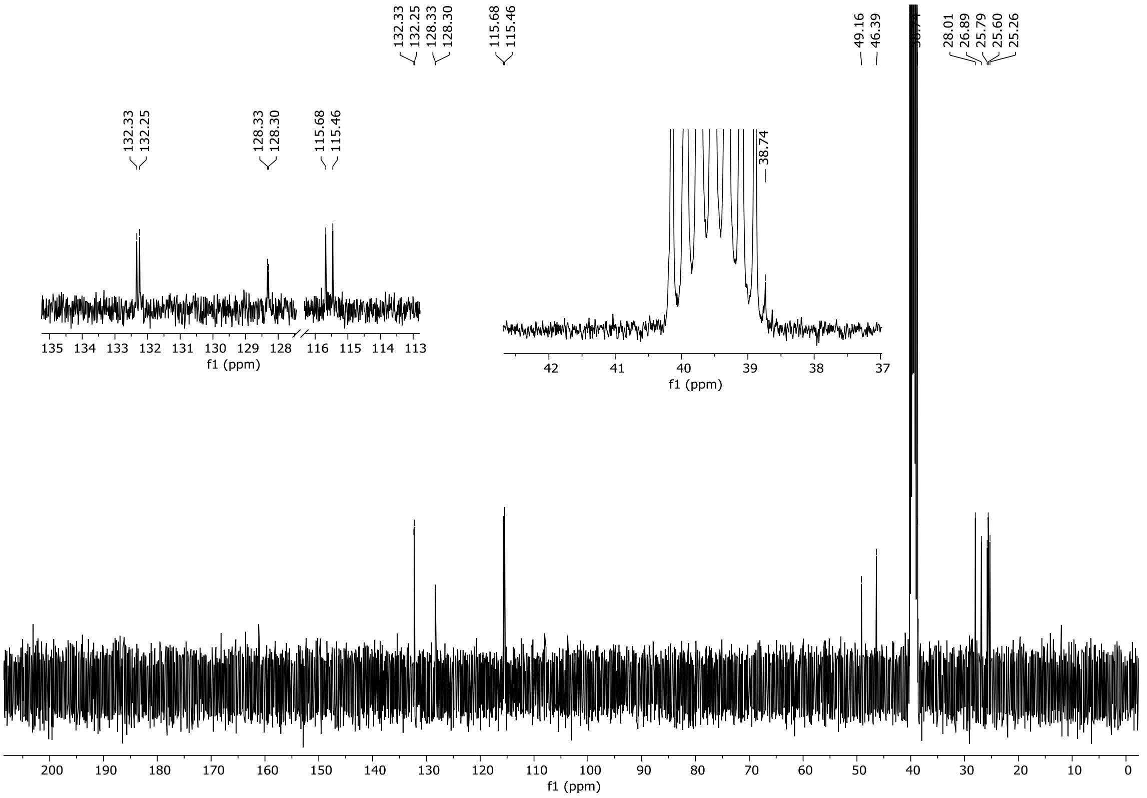

Figure S36: ^{1}H NMR spectrum of *N*[1]-(4-fluorobenzyl)-1,8-diaminooctane×2TFA (**17**)

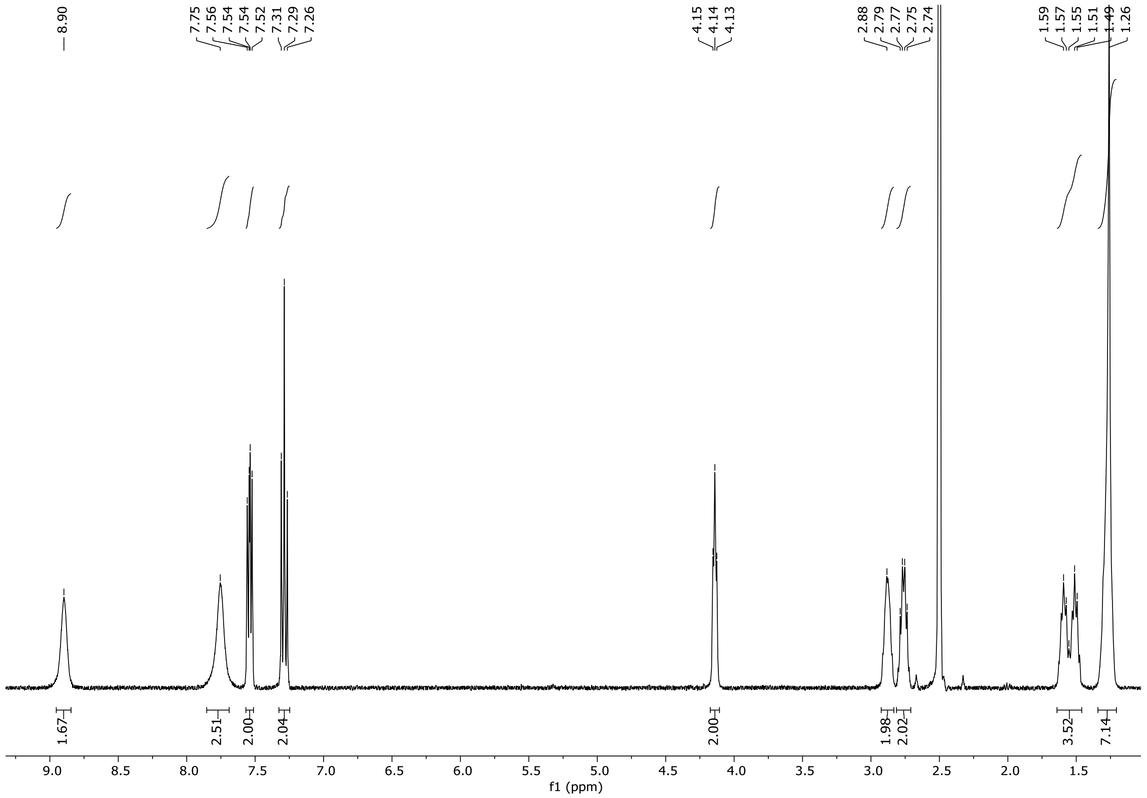

Figure S37: ^{13}C NMR spectrum of *N*[1]-(4-fluorobenzyl)-1,8-diaminooctane×2TFA (**17**)

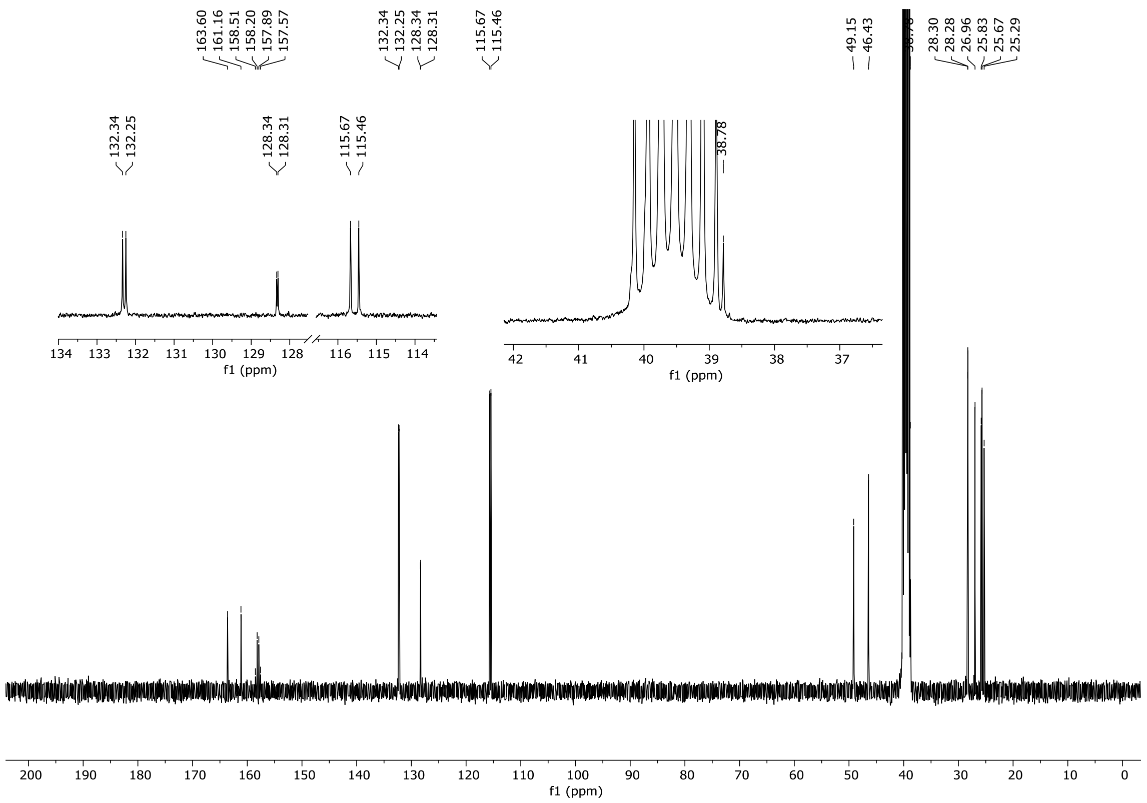

Figure S38: ^{1}H NMR spectrum of *N*^{1}-(4-fluorobenzyl)-spermine×4TFA (**18**)

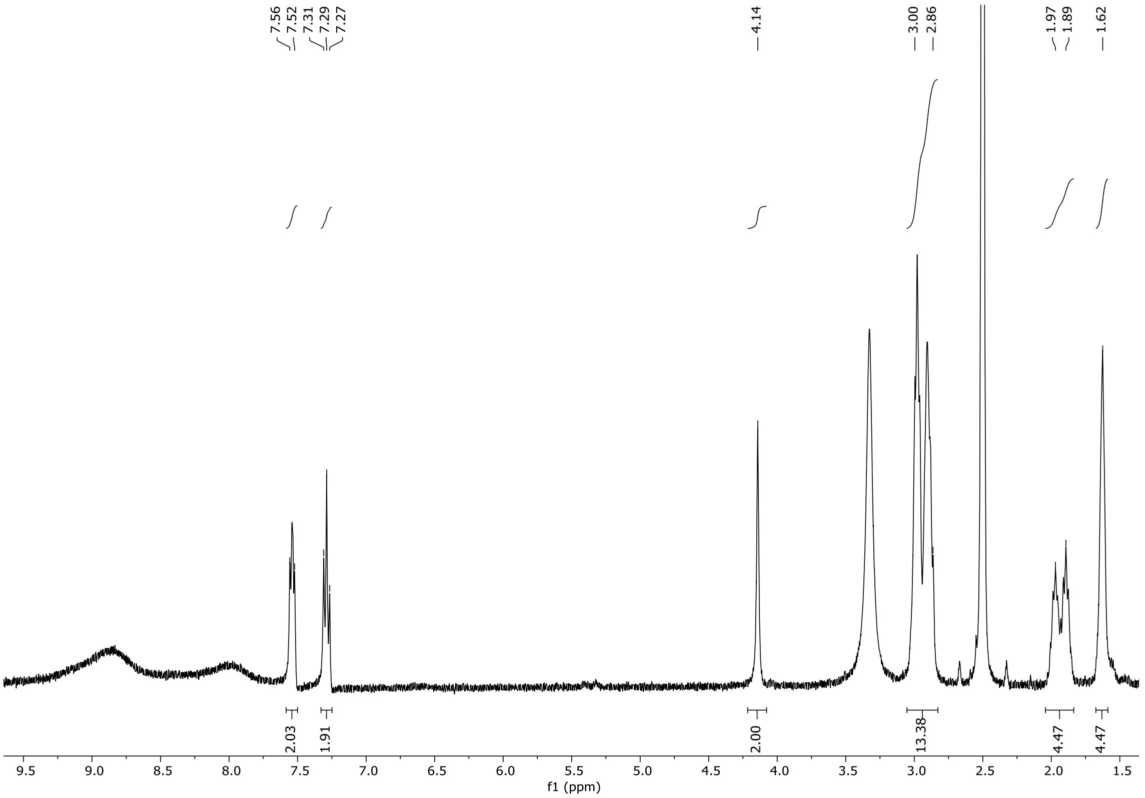

Figure S39: ^{13}C NMR spectrum of *N*^{1}-(4-fluorobenzyl)-spermine×4TFA (**18**)

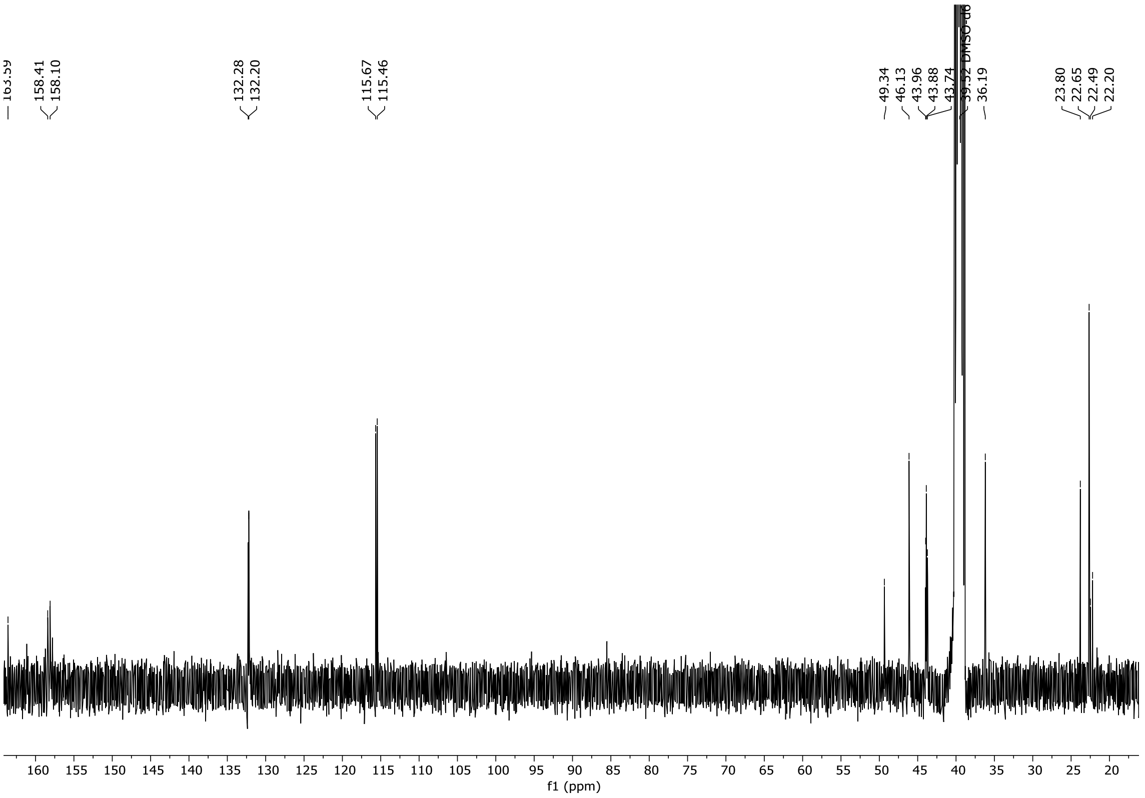

Supplement: Supplementary file 1 [file molecules-26-07012-s001.zip › molecules-1421976-supplementary.pdf]
